# Multiple CaMKII Binding Modes to the Actin Cytoskeleton Revealed by Single-Molecule Imaging

**DOI:** 10.1016/j.bpj.2016.06.007

**Published:** 2016-07-26

**Authors:** Shahid Khan, Ianina Conte, Tom Carter, K. Ulrich Bayer, Justin E. Molloy

**Affiliations:** 1 Molecular Biology Consortium, Lawrence Berkeley National Laboratory, Berkeley, California; 2 Cardiovascular and Cell Science Research Institute, St. George's University of London, London, UK; 3 Cell Biology and Genetics, St. George's University of London, London, UK; 4 Department of Pharmacology, University of Colorado Denver, Aurora, Colorado; 5 The Francis Crick Institute, Mill Hill Laboratory, London, UK

## Abstract

Localization of the Ca^2+^/calmodulin-dependent protein kinase II (CaMKII) to dendritic spine synapses is determined in part by the actin cytoskeleton. We determined binding of GFP-tagged CaMKII to tag-RFP-labeled actin cytoskeleton within live cells using total internal reflection fluorescence microscopy and single-molecule tracking. Stepwise photobleaching showed that CaMKII formed oligomeric complexes. Photoactivation experiments demonstrated that diffusion out of the evanescent field determined the track lifetimes. Latrunculin treatment triggered a coupled loss of actin stress fibers and the colocalized, long-lived CaMKII tracks. The CaMKII*α* (*α*) isoform, which was previously thought to lack F-actin interactions, also showed binding, but this was threefold weaker than that observed for CaMKII*β* (*β*). The *β*E′ splice variant bound more weakly than *α*, showing that binding by *β* depends critically on the interdomain linker. The mutations *β*T287D and *α*T286D, which mimic autophosphorylation states, also abolished F-actin binding. Autophosphorylation triggers autonomous CaMKII activity, but does not impair GluN2B binding, another important synaptic protein interaction of CaMKII. The CaMKII inhibitor tatCN21 or CaMKII mutations that inhibit GluN2B association by blocking binding of ATP (*β*K43R and *α*K42M) or Ca^2+^/calmodulin (*β*A303R) had no effect on the interaction with F-actin. These results provide the first rationale for the reduced synaptic spine localization of the *α*T286D mutant, indicating that transient F-actin binding contributes to the synaptic localization of the CaMKII*α* isoform. The track lifetime distributions had a stretched exponential form consistent with a heterogeneously diffusing population. This heterogeneity suggests that CaMKII adopts different F-actin binding modes, which is most easily rationalized by multiple subunit contacts between the CaMKII dodecamer and the F-actin cytoskeleton that stabilize the initial weak (micromolar) monovalent interaction.

## Introduction

The calcium calmodulin-dependent kinase (CaMKII) is a multifunctional kinase that has a prominent role in long-term potentiation (LTP) (1, 2, 3). The four major isoforms of vertebrate CaMKII have ∼40 splice variants and are expressed in diverse tissues (3). Two isoforms, CaMKII*α* (<*α*>) and CaMKII*β* (<*β*>), are dominant in the brain and their relative expression levels vary among different regions of the brain as well as during development (4). Their relative levels also vary within individual neurons between the cell body and dendritic/axonal processes (2). CaMKII has a prominent structural role in hippocampal dendritic spines, the postsynaptic computational units for LTP. CaMKII concentrations in spines are high (5), consistent with its structural role. The <*β*> isoform targets *αβ* hetero-oligomers to dendritic spines by binding to the spine actin cytoskeleton (6). Synaptic stimulation triggers CaMKII sequestration to dendritic spines and the postsynaptic density (PSD) within a few seconds of stimulation (7, 8, 9, 10). This rapid sequestration is coupled to actin polymerization and expansion of the stimulated spine (11). Expansion is due to the direct effects of CaMKII on the actin cytoskeleton (12, 13) as well as to indirect effects mediated by the activation of other kinases (14). The increase in spine size persists after termination of the stimulus-induced calcium transient. CaMKII levels in stimulated spines are also increased due to association with the PSD, in particular, the NMDA receptor GluN2B subunit (15) and the enlarged actin cytoskeleton (16). In the longer term, CaMKII promotes axonal branching and outgrowth (17).

The neuronal isoforms have highly homologous kinase and association domains, but the linker that connects these two domains is variable in sequence and length (1). The individual subunits assemble into homo- or hetero-oligomers of variable isoform compositions, and the atomic structure of the dodecameric enzyme has been previously described (18). The <*α*> and <*β*> isoforms form 12 subunit homo-oligomers of similar size, with one study reporting a slightly smaller <*β*> oligomer (19). Calmodulin binding to the regulatory segment relieves inhibition, and transphosphorylation activates the enzyme at <*α*>T286 (T287 in the other isoforms), which confers autonomous activity to the enzyme.

Binding of <*β*>, but not <*α*>, to the actin cytoskeleton has been shown by various approaches, including colocalization, fluorescence photobleaching, and pharmacological manipulations in neuronal and non-neuronal cell cultures (6, 20, 21, 22, 23). In vitro sedimentation assays and electron microscopy have demonstrated the <*β*>-dependent formation of F-actin bundles (12, 22, 23, 24). Activation of <*β*> by both autophosphorylation and the phosphomimetic T287D mutation (22) abolishes actin bundling activity. Furthermore, an alternative splice variant, <*β*E′>, which has a short linker similar to that of <*α*>, does not bind or bundle F-actin in pull-down assays. The differences observed with the mutants in pull-down assays are consistent with colocalization in neuronal cell cultures. Pyrene fluorescence measurements (12) have shown that both <*α*> and <*β*> isoforms bind globular (G-) actin, and <*β*> binds with 2.4 *μ*M affinity and a stoichiometry of 12 actin monomers per oligomer (24). However, quantitative estimates of the affinity of <*β*> or <*α*> for F-actin, or of modulation via activation through stimulation or mutation, are not available.

Here, we characterized the association of CaMKII with labeled F-actin in live human umbilical vein endothelial cells (HUVECs) (25) by using total internal reflection fluorescence microscopy (TIRFM) to image and track single molecules (26, 27). We previously exploited this approach to study motor proteins, ion channels, and G-protein-coupled receptors (27, 28, 29, 30). Here, we extended the method to measure the association of enhanced green fluorescent protein (eGFP)-tagged CaMKII native and mutant proteins with red fluorescent protein (RFP)-tagged actin to mark the cytoskeletal structures. Single-molecule tracking experiments have shown that actin depolymerization increases CaMKII mobility in dendritic spines, and revealed different, heterogeneous mobility distributions for stimulated versus unstimulated states (16). We used HUVECs as a model system because they are ideal for TIRF imaging, have a defined cytoskeletal architecture, and are amenable to transient transfection methods. Our measurements show that both neuronal CaMKII isoforms bind cytoskeletal actin, but with affinities that differ by threefold over the first decade range of a log-normal binding curve. Our results explain why association of <*α*> may have been overlooked in earlier studies, and have implications for CaMKII transport and cytoskeletal remodeling within neurons.

## Materials and Methods

All biochemicals were sourced from Sigma-Aldrich (Poole Dorset, UK) unless noted otherwise.

### TIRFM

**Figure 1:**
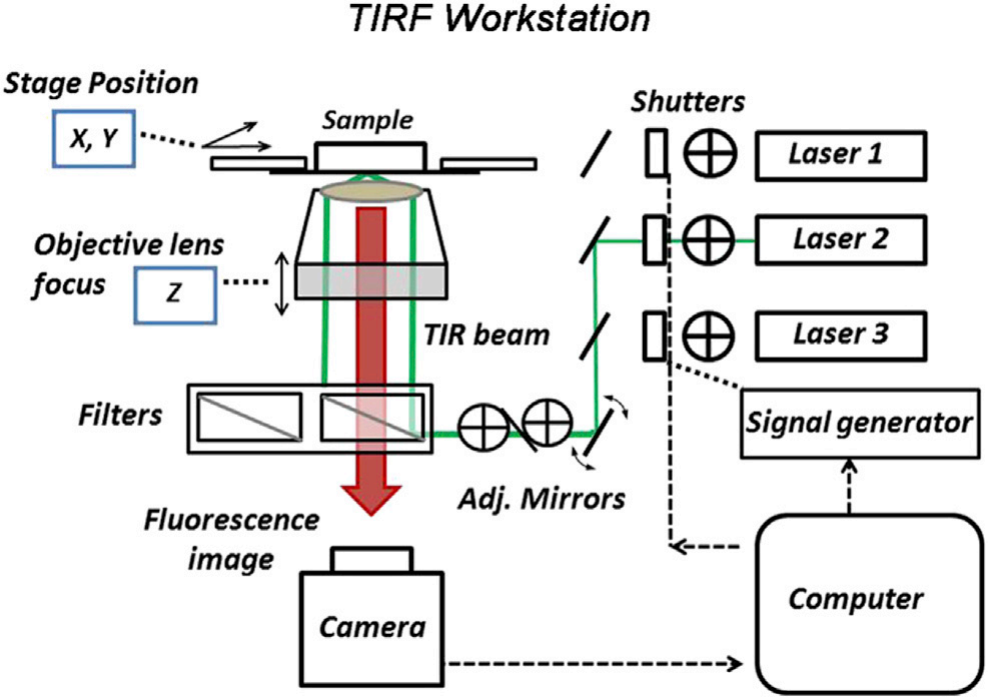
TIRF workstation. The choice of the laser excitation wavelength (laser 1 = 561 nm; laser 2 = 488 nm) was computer controlled; excitation (*green line*) and fluorescence emission (*red arrow*) light paths are shown. The TIRF incident angle was adjusted by an external mirror. The microscope stage and objective lens employed piezo-positioners to control specimen position and image focus. Images were acquired with an EMCCD camera. A waveform generator set the duration, delay, and frequency of photoactivation pulses (laser 3 = 405 nm), also in TIRF mode. Separate, exchangeable filter cassettes were used for GFP and tRFP fluorescence.

We used a custom-built TIRF microscope workstation based on an inverted microscope (Nikon Eclipse, TE 2000U; Nikon, Kingston-upon-Thames, UK) (Fig. 1). Complete details are provided in Supporting Materials and Methods in the Supporting Material.

### In vitro assays

For use as a single fluorophore calibration specimen, we immobilized GFP molecules on the surface of a microscope flow cell with a GFP antibody by first filling the flow cell with a phosphate-buffered saline (PBS) solution (pH 7.4) containing 5 *μ*g/mL (3 nM) polyclonal anti-GFP antibody (Abcam, Cambridge, UK) as described previously (27). This solution was left to incubate in the flow cell for 5 min and then washed with PBS supplemented with 0.5 mg/mL bovine serum albumin to block regions of bare coverglass. The solution was then replaced with PBS containing 10 ng/mL (0.37 nM) GFP (Clontech, Palo Alto, CA) for 5 min, and unbound protein was washed out of the flow cell by several washes with assay buffer (AB^−^ (20 mM imidazole (pH 7.4), 50 mM KCl, 2 mM EGTA, 4 mM MgCl_2_)) before it was viewed by TIRFM. The molecules were imaged in degassed and argon-purged AB^−^ supplemented with an oxygen-scavenger system consisting of 3 mg/mL glucose, 0.5 mg/mL catalase, 0.2 mg/mL glucose oxidase, and 20 mM dithiothreitol. Using the antibody-immobilized GFP molecules as a control sample, we measured the single fluorophore intensity as a function of excitation power. The average value measured over several hundred fluorophores was linear with the laser power. The mean single fluorophore intensity could therefore be used as an independent internal check of excitation power in subsequent experiments.

### Cell culture

CaMKII fusion proteins tagged with monomeric eGFP (GFP) or photoactivatable eGFP (PaGFP) carrying the A206K mutation have been described previously (22, 31, 32, 33, 34). The GFP tag does not interfere with kinase activity or holoenzyme assembly (24), and immunoelectron microscopy has shown that native CaMKII sequesters to the PSD of dendritic spines (35) with kinetics similar to those reported by the tagged proteins (22, 36). We studied the following tagged actin fusion proteins: mCherry-actin (37), tagRFP-actin (38), and mTurquoise2-actin (39). We chose tagRFP-actin (tRFP-actin) for its brightness, photostability, and expression level (40). The plasmids encoding GFP-CaMKII and tRFP-actin constructs were mixed and cotransfected into primary HUVECs or Cos7 cells at 70–80% confluence, primarily by nucleofection (Nucleofector Model 2b; Lonza, Blackley, UK). Alternatively, Lipofectamine-2000 (Life Technologies, Paisley, UK) transfection was used as previously described (41). With either method, the transfection efficiency was typically >50%. The cells were plated on poly-lysine-coated dishes (Lab-Tek chambered borosilicate, #1 coverglass; Nunc, Rochester, NY) in Dulbecco’s modified Eagle’s medium with added 10% fetal bovine serum and streptomycin (50 *μ*g/mL). Cell culture dishes were removed from the CO_2_ incubator (Galaxy R; Scientific Laboratory Supplies, East Riding of Yorkshire, UK) 24–36 h after transfection. These incubation times were optimal for visualizing single GFP-CaMKII molecules. TIRF imaging was conducted at 25°C within an hour after the samples were removed from the incubator.

The HUVECs we chose as a model system for most of our TIRF imaging experiments attach firmly to the culture dish substrate and have long ventral stress fibers (42) that form oriented arrays. Other cytoskeletal substructures (i.e., arcs (43) and filopodia) are also present. Although HUVECs express a variant CaMKII*δ*6 isoform (44), they do not natively express the <*α*> or <*β*> isoforms found in neurons. Expression was monitored by epifluorescence, and cell morphology was determined by phase contrast. In addition to morphology, we checked the integrity of the physiological state by noting an absence of CaMKII aggregation caused by high pH or calcium (36).

### Single-molecule image analysis

A typical experiment involved a set of cotransfections of the plasmid encoding tRFP-actin with a plasmid encoding a GFP-CaMKII fusion (two dishes per CaMKII construct; up to four constructs per experiment). Control dishes cotransfected with plasmids encoding tRFP-actin and GFPCaMKII*β* were included in each experiment to assess the viability of the primary culture. First, tRFP-actin fluorescence was used to identify transfected cells, and then GFP-fluorescence was recorded. Many thousands of single-particle tracks were obtained for each construct using >12 cells from four different culture dishes and two separate experiments. Details regarding the single-particle tracking algorithm and the analytical measures used are provided in Supporting Materials and Methods.

Multiple analysis of variance (ANOVA) and simultaneous pairwise *t*-tests were conducted in R (https://www.r-project.org/) as detailed in (45). The variance was the sum of the variation within and between groups normalized by their degrees of freedom. The probability (*p*-value) that differences between populations were significant was then computed from the *F*-value (*F*). Significant differences reported by ANOVA were then tested by means of simultaneous, pairwise *t*-tests with default Holm correction for multiple testing.

## Results

Our experimental study consisted of two parts, First, we used dual-color TIRFM to visualize and track (27) individual GFP-tagged <*α*> and <*β*> isoforms in HUVECS, and derived their properties from population statistics and spatial colocalization with F-actin cytoskeletal structures. We then studied different mutants and pharmacological agents to understand the structural basis of CaMKII association with F-actin.

### Assay development

#### Visualization of GFP molecules in control specimens and live cells

**Figure 2:**
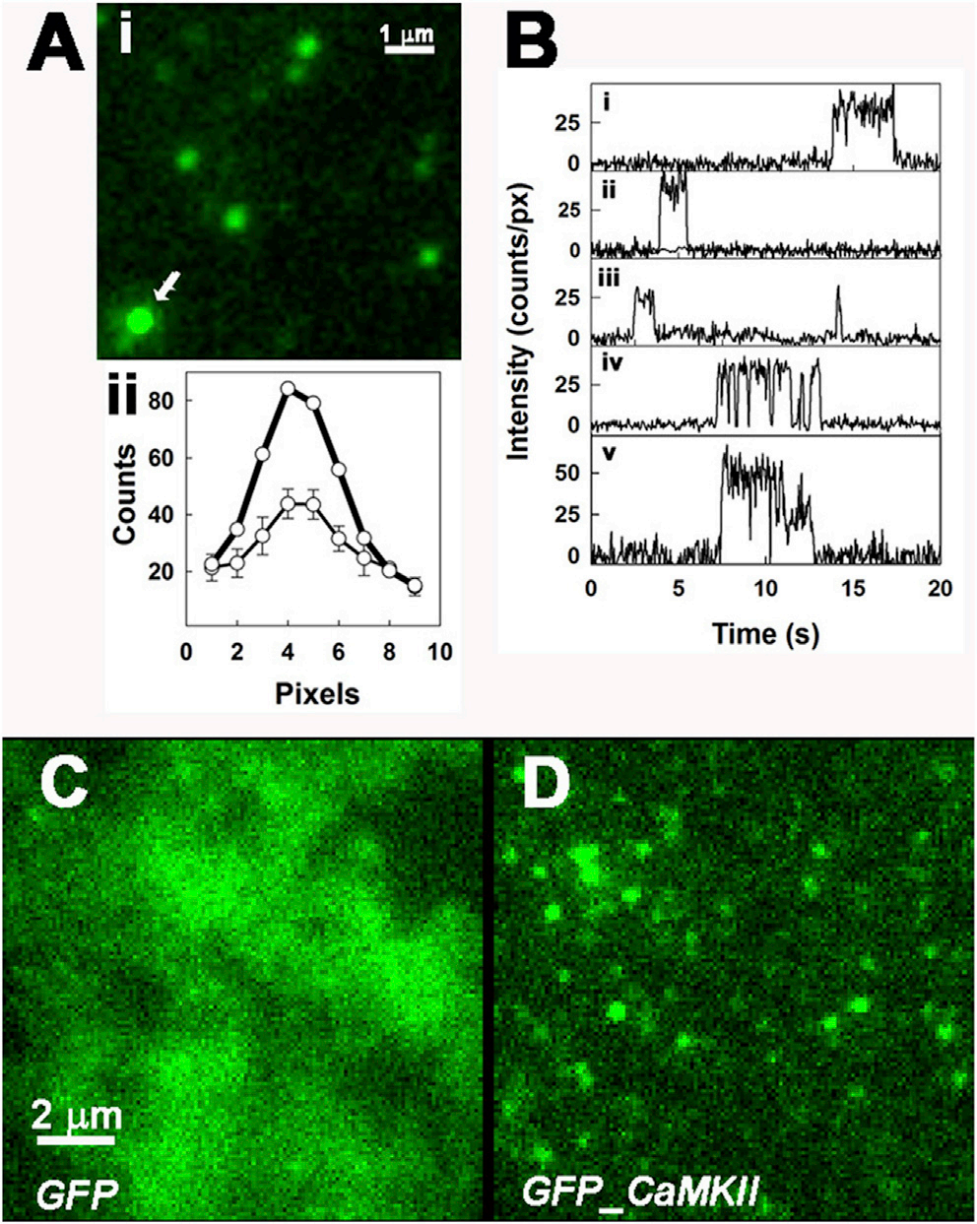
TIRFM visualization of GFP in vitro and in living cells versus GFP-CaMKII. (*A*) *i*: Antibody-immobilized GFP molecules (10-frame averaged image)). *ii*: Line intensity profiles of the four spots in field center, top and right (± standard error (SE), *thin line*), and of the brighter spot (*arrow*) show the diffraction-limited size. (*B*) Intensity-versus-time records of spots shown in *A*(*i*), illustrating single-step photobleaching (*i–iii*), blinking behavior (*iv*), and double-step photobleaching of the brighter spot (*v*).The single-step modal value was 27.5 ± 2.5 counts/pixel (doubling and tripling occurs when fluorophore PSFs overlap). (*C*) Single video frame (50 ms exposure) of a HUVEC expressing GFP alone, showing that motion blurring prevents single fluorophore observation. (*D*) Single video frame of a HUVEC expressing GFP-CaMKII*β*, showing that discrete fluorescent spots are now visible.

We visualized antibody-immobilized GFPs at low surface density (<1 *μ*m^−2^) using TIRFM to establish the emission intensity of individual GFP molecules under our standard imaging conditions. Individual GFPs were readily identified as discrete fluorescent spots that had a diffraction-limited point spread function (PSF) with a characteristic spot intensity (Fig. 2 *A*). The spots had a mean duration of 2.0 ± 0.4 s and exhibited single-step photobleaching with a unitary intensity of 27.4 ± 2.2 counts/pixel. Brighter spots with twofold greater intensity exhibited two-step photobleaching (Fig. 2 *B*). Next, we obtained TIRFM video recordings of cultured HUVECs and Cos7 cells that were expressing GFP. In contrast to the video recordings of antibody-immobilized GFP molecules, the GFP fluorophores within cells could not be resolved (Fig. 2 *C*). This was because rapid diffusive motion within the cytosol caused image blurring during the frame acquisition period, as explained below.

#### Visualization of homomeric GFP-CaMKII*β* complexes in the cellular cortex

In marked contrast to cell cultures expressing GFP molecules alone (see above), single fluorescent particles were visualized by TIRFM in cell cortices when GFP-tagged <*β*> (henceforth termed *β*) was expressed (Fig. 2 *D*). This discrepancy can be explained by attenuation of the spot intensity by motion blurring during the 50 ms frame acquisition period (*δt*). The attenuation factor of the computed centroid is given by the ratio of the area covered by the diffusing particle during a single video frame = *π*(*δx*)^2^ (where *δx* = (4*D*.*δt*)^1/2^, and *D* is the diffusion coefficient) and the area that captures 90% of the object’s PSF (here a 3 × 3 pixel region on the camera) = 0.9 *μ*m^2^.

The expected lateral diffusion coefficients, *D*_Stokes_, for the relevant species were computed from the diffusion equation:

(1) $$D_{Stokes}=\frac{k_{b}T}{6\pi \eta a_{s}},$$

where Stokes radius *a*_s_ = (3M/4*π*A*σ*)^1/3^, M is the molecular mass (kDa), A is Avogadro’s number, *σ* is the protein density (1300 kg/m^3^) (46), and *η* is the cortical viscosity (0.0032 Pa.s) (47, 48). This gives an estimated *D*_Stokes_ for GFP (M = 27 kDa; *a*_s_ = ∼2 nm) of ∼30 *μ*m^2^s^−1^ and diffusive motion blurring during a 50 ms video frame of ∼20 *μ*m^2^. Therefore, the expected reduction in fluorescence intensity (per pixel) is 20/0.9, ∼23-fold. This explains why freely diffusing GFP molecules were not resolved at the video imaging rates. Since *D*_Stokes_ varies inversely as the cube root of the molecular mass, we were also unable to resolve the GFP-<*a*> (henceforth *α*) mutant, which is monomeric due to deletion of the association domain (*α*Δ316) (M = 62 kDa) and tRFP-G-actin (M = 70 kDa), as both exhibit a >15-fold estimated attenuation of spot intensity due to motion blurring. We were able to satisfactorily visualize *β* molecules because they form dodecameric complexes (M = 87 × 12 = 1044 kDa) (18, 49). Thus, the intensity attenuation by motion blurring (∼7-fold) is more than compensated for by the 12-fold increase in intensity due to the increased number of GFPs.

#### Decoration of actin stress fibers with single GFP-CaMKII*β* holoenzymes

We used two-color TIRFM to image *β* (excited at 488 nm) and tRFP-actin (excited at 561 nm) to characterize *β* complexes interacting with F-actin cortical structures. TIRFM of HUVECs transfected with t-RFP actin revealed long linear fibers in the actin cortex. The morphology was consistent with ventral stress fibers (42), and these structures will henceforth be referred to as such. Approximately 100 video frames were averaged to enhance the relatively static fibers above the background of rapidly diffusing G-actin monomers. The averaged tRFP-actin image was then overlaid onto TIRFM video recordings of *β* molecules to reveal their movement within the cytosol and their association/dissociation with the tRFP-tagged F-actin structures (Movie S1).

Individual GFP-fluorescent spots were identified and tracked in the video sequences to yield spatiotemporal trajectories (300–3000 per record) of individual objects. Tracks were generated by linking centroids for successive frames. Apparent diffusion coefficients (*D*_lat_) were computed from the centroid frame-to-frame displacements (Δ*x*):

(2) $$D_{lat}={\left(\Delta x\right)}^{2}/\left(4t^{c}\right).$$

**Figure 3:**
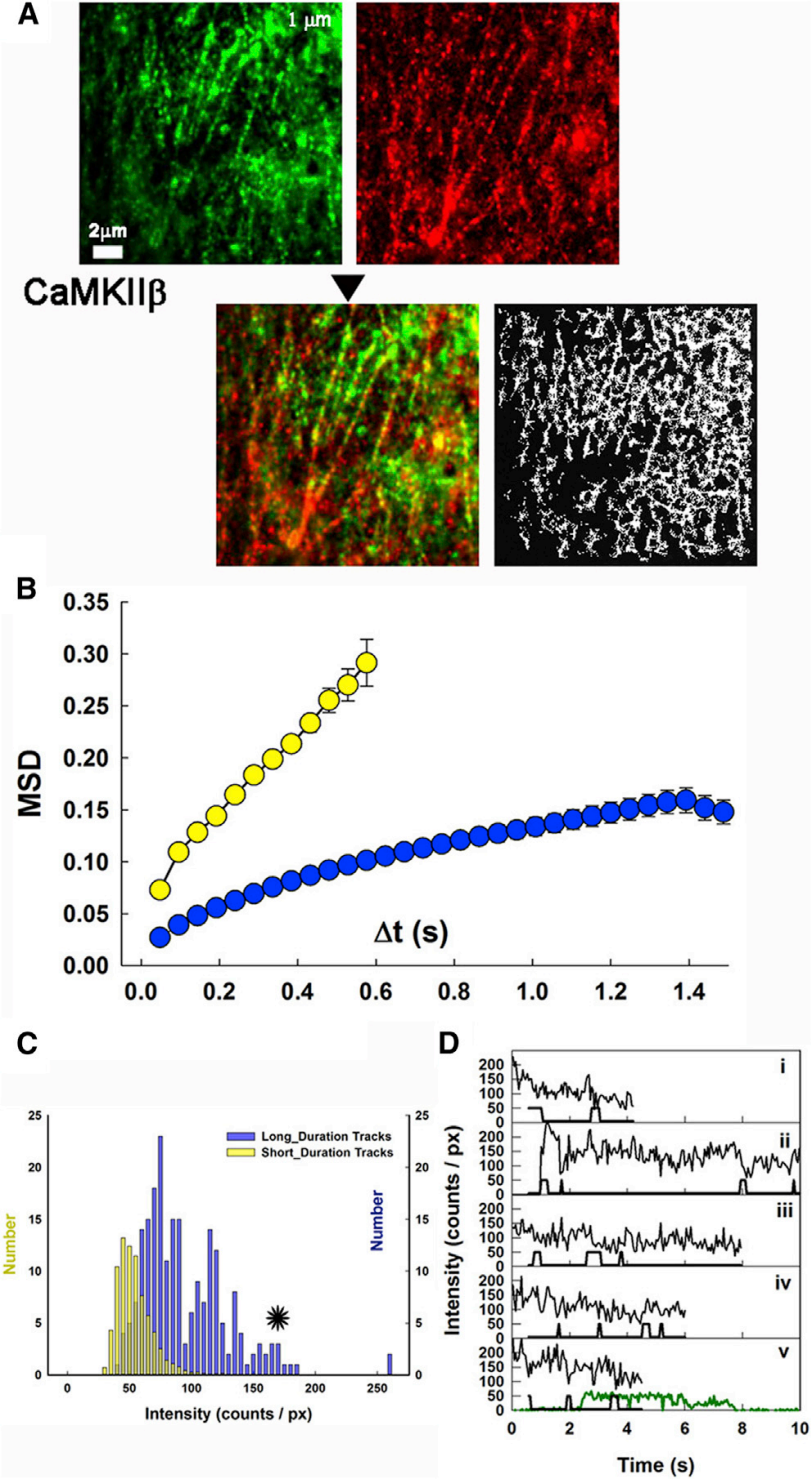
CaMKII*β* decoration of the actin cytoskeleton visualized by two-color TIRFM. (*A*) Averaged images of *β* (*left panel*, *green*, 400 frames) and tRFP-actin (*right panel*, *red*, 100 frames). Mean tRFP intensity = 119 ± 9 counts/pixel. The bottom panels (*arrow*) show the two frames superimposed (*P*_pix_ = 0.27, *P*_rand_ = 0.09 ± 0.07) (*left*) and the single-particle tracks (*right*) accumulated over 10 s of video (Movie S1). (*B*) MSD-versus-time interval (Δ*t*) for the total population of tracks (*white circles*) and short-lived (*yellow circles*) and long-lived (*blue circles*) track subpopulations (± standard deviation (*σ*)). The initial gradient of the short-lived track data gives *D*_lat_ = 0.28 *μ*m^2^/s, whereas that of the long-lived tracks gives 0.04 *μ*m^2^/s. Total number of tracks, *n* = 12,723. (*C*) Intensity histograms for the short-lived track subpopulation (*yellow bars*) and long-lived track subpopulation (*blue bars*). The asterisk (*black*) marks the region of the histogram that was used to analyze photobleaching. (*D*) Sample intensity-versus-time plots for some of the objects from the asterisk-marked region. Stepwise intensity changes as detected by Student’s *t*-test (Fig. S1) are marked immediately below each trace to indicate sudden intensity transitions. The starting intensity for each spot was >170 counts/pixel, which is ∼8-fold greater than the unitary GFP intensity. The green line (in the *lowest panel*), is the two-step immobilized GFP photobleaching, redrawn from Fig. 2*B*(*v*), shown for reference.

For free diffusion, *c* = 1. For confined diffusion, *c* = >1 and the denominator preexponent = <4. Individual tracks terminated when the object intensity dropped below the detection threshold due to diffusion from the excitation region (evanescent field), photobleaching, or tracking errors (considered below). Superposition of the image showing all of the particle tracks obtained over one video recording (lasting 25 s) onto the averaged tRFP-actin image provided a measure of colocalization (Pearson’s correlation coefficient, *P*_*pix*_), as described in Supporting Materials and Methods. It was clear that *β* associated with the cortical actin fibers (Fig. 3 *A*).

#### Dynamics of the interaction between CaMKII*β* and the actin cytoskeleton

Automated single-particle tracking (27) was used to identify and track individual *β* complexes. The object tracks were characterized with the measures defined in Materials and Methods. Short-lived particle trajectories (*t* < 0.58 s; Fig. 3 *B*, *yellow symbols*) closely approximated Brownian motion. In contrast, the plot for longer-lived trajectories (*t* > 2.5 s; Fig. 3 *B*, *blue symbols*) was nonlinear, with little increase in the mean-square deviation (MSD) beyond Δ*t* > 1.2 s. Further analysis showed that the binned subpopulation of short-lived tracks had a unimodal intensity distribution with a lower mean relative to the parent population, whereas the subpopulation of longer-lived tracks had higher intensity relative to the parent population and the intensity distribution was greatly skewed toward higher values (Fig. 3 *C*). The different subpopulation characteristics are consistent with the notion that tracks from weakly bound, more mobile molecules have a short duration and dominate the <0.5 s subpopulation. In contrast, more strongly bound molecules dominate the >2.5 s subpopulation, with lower average *D*_lat_. The modal intensities for both subpopulations are lower than expected for the multimeric (10–12 subunits) tagged *β* holoenzymes. Thus, although at first it may seem that the correlation between intensity and mobility differences is simply due to a difference in aggregate size, it is better explained by an intensity attenuation due to motion blurring (see above). To ascertain whether this was the case, we examined single spots and tracks.

To test for multisubunit states, we measured stepwise changes in fluorescence intensity. Spots immobilized on actin stress fibers had the highest intensities, but they exhibited PSF-limited spatial profiles similar to those obtained for single GFP fluorophores (Fig. 2 *A*). A small subset of such spots was analyzed (Fig. 3 *D*). The average initial intensity was ∼8-fold greater (171.6 ± 11.5 counts/pixel) than that measured for individual GFP molecules in vitro (∼27 counts/pixel). We used a running Student’s *t*-test to detect significant jumps in local mean intensity over adjacent sections of data (Fig. S1 in the Supporting Material). The mean intensity drop for each stepwise change in intensity was 22.1 ± 2.0 counts/pixel and the mean step duration was 2.6 ± 0.4 s, which are similar to the values obtained for single GFP molecules immobilized in vitro (27.4 ± 2.2 counts/pixel and 2.0 ± 0.4 s). Many of these spots showed a severalfold greater final intensity drop (e.g., 90–0 counts/pixel (spot *v*)) relative to the 27.4 ± 2.2 counts/pixel drops obtained for single GFP photobleaching. Simultaneous photobleaching of multiple (three for spot *v*) GFP fluorophores is not likely. Instead, the final intensity drops presumably report the dissociation of *β* holoenzymes from the fibers and diffusion out of the evanescent field before all their fluorophores have bleached.

Sample tracks and their MSD versus Δ*t* plots were analyzed next (Fig. S2). In addition to high intensities, the long-lived tracks had highly nonlinear MSD versus Δ*t* plots, and the MSD and Δ*t* correlation was abolished for intervals greater than a few frames, consistent with immobilization as validated by an examination of the single tracks. Centroid intensity inversely correlated with mobility in the short-lived tracks of diffusing spots, with values consistent with the motion-induced sevenfold attenuation relative to the intensity of immobilized holoenzymes. The slopes (MSD versus Δ*t*) of these short tracks correlated with the fraction of time during which they were mobile. Analyses of the single-spot photobleaching and single tracks show that motion blurring is responsible for the observed mobility-intensity correlations in the subpopulation distributions. We conclude that the rapid decrease in the track population with time is governed predominantly by the diffusion of unbound molecules out of the evanescent field.

#### Filopodia kymographs support the tracking analysis

Cultured HUVECs exhibit numerous filopodia, which are actin-rich tubular extensions >2 *μ*m long and ∼150 nm in diameter. Some of the filopodia protruded close to the coverslip and were visualized in our video recordings by the evanescent field excitation. This gave us the opportunity to track GFP-tagged molecules that were essentially constrained to a single dimension independently of the depth, *z*, of the evanescent field. The molecule movements were suitable for kymograph analysis. We straightened the image data by using spline fits to the overall filopodial shape, and then extracted a linear strip of image pixels to form the abscissa in the kymograph time-series image (Fig. S3).

The *β* complexes produced punctate images on each video frame, and their motion within the filopodium then created a pattern of vertical trajectories (i.e., along the ordinate, time axis). The trajectories consisted of linear, bright segments that were tilted slightly toward the cell body (at ∼1.5 *μ*m/min), consistent with complexes binding tightly to actin and reporting the slow rearward flow of the central F-actin bundle of the filopodium (50). These events were interspersed with haphazard, dim trajectories as the particles dissociated from actin and diffused within the body of the filopodium. Both types of trajectories were observed for closely adjacent objects within the same filopodium over the same time window, indicating that dim trajectories result from mobility of the *β* complexes within the filopodium rather than movement of the filopodium relative to the glass coverslip.

Our initial goal was to achieve a time-resolved characterization of bound and free episodes of *β* molecules constrained within the evanescent field by the filopodia. However, to our surprise, the kymographs also revealed that both *α* and *β* associated with filopodial F-actin.

#### F-actin dependence of CaMKII *α* and *β* lifetime distributions by evanescent field fluorescence photoactivation microscopy

**Figure 4:**
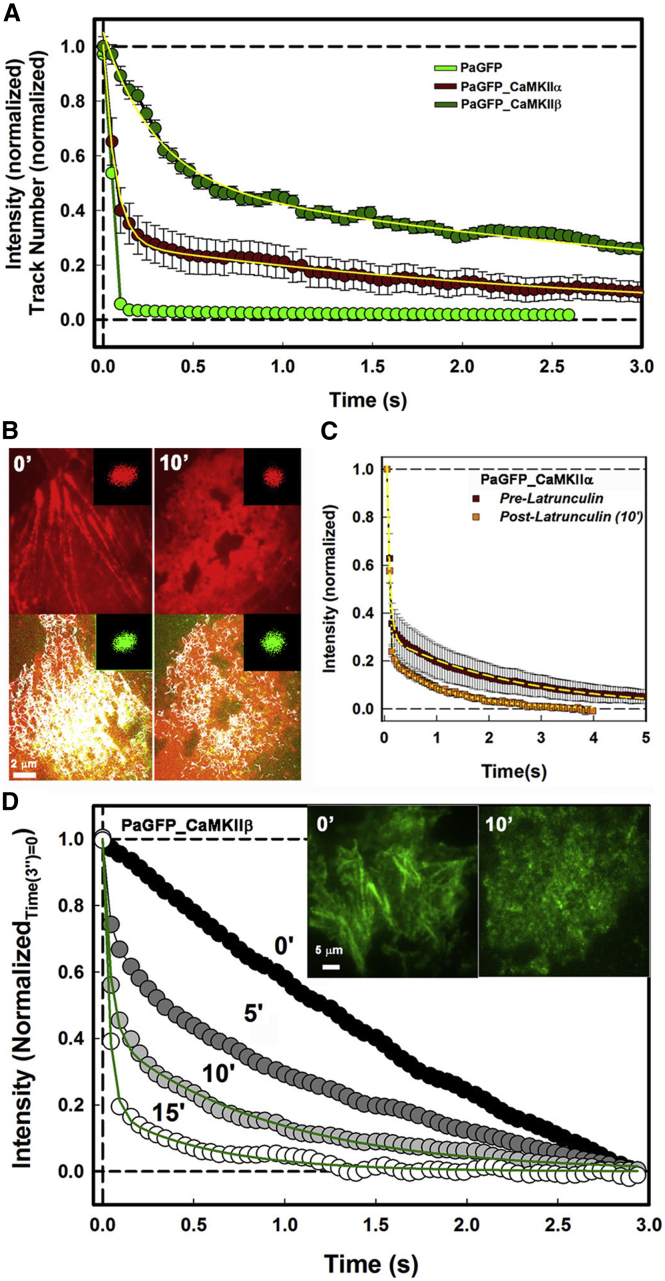
Results from photoactivated localization microscopy TIRFM and latrunculin treatment show that both CaMKII isoforms associate with the actin cytoskeleton. (*A*) Normalized fluorescence decay curves of PaGFP and PaGFP-CaMKII fusion proteins after photoactivation by a 405 nm laser (at *t* = 0). Decay was measured as single-molecule track lifetimes. Time 0 is the time required to exceed the five-frame track duration threshold (0.24 s). The data were least-square fitted to two exponentials (*yellow lines*): PaGFP-*α* = 0.72 ± 0.01(e^(−15.2±0.5*t*)^) + 0.28 ± 0.01(e^(−0.35±0.07*t*)^), *n* = 4306; PaGFP-*β* = 0.51 ± 0.01(e^(−3.56±0.08*t*)^) + 0.49 ± 0.01(e^(−0.24±0.07*t*)^), *n* = 11,160. In contrast, photoactivated PaGFP fluorescence intensity measured over the image field decayed by >50% within 0.1 s (two frames). (*B*) HUVEC stress fibers after 10 min (10’) treatment with latrunculin (5 *μ*M). The top panels (*red*) show the averaged tRFP-actin images: although there is little change in the total fluorescence (97 ± 17 counts/pixel (before latrunculin treatment); 100 ± 23 counts/pixel (after latrunculin treatment)), the fibers disappear after treatment. The bottom panels show PaGFP-CaMKII*α* (*green*) and single-particle tracks (*white lines* (*n* = 3777 (0’) and 1573 (10’)) superimposed on actin (*red*). Insets: FT spectra (tRFP-actin (*red*); GFP (*green*)). (*C*) PaGFP-CaMKII*α* fluorescence decay before and after latrunculin treatment. Dual exponential fits: 0.71 ± 0.01(e^(−18.1±0.5*t*)^) + 0.29 ± 0.01(e^(−0.39±0.01*t*)^) (*dashed yellow line*) (0’); 0.8 ± 0.01(e^(−19.9±0.8*t*)^) + 0.21 ± 0.01(e^(−0.92±0.04*t*)^) (*dotted yellow line*) (10’). (*D*) Fluorescence intensity decay curves of PaGFP-*β* at various times (in minutes) after addition of latrunculin (5 *μ*M) to a Cos7 cell culture. Intensity was normalized to unity at *t* = 0 s (*t*_0_) and zero at *t* = 3 s (*t*_3_); (*t*_3_/*t*_0_) ∼50%. Control fit (unnormalized): (0.26 ± 0.01) + (0.74 ± 0.01(e^(−0.28±0.01t)^). Fits after latrunculin treatment (*green lines*): 0.58(e^(−24.3*t*)^) + 0.42(e^(−1.2)^) (10’); 0.81(e^(−29.3*t*)^) + 0.19(e^(−1.8)^) (15’). Inset: Filamentous structures visualized when PaGFP-*β* was photoactivated in the absence of latrunculin (0’) were not observed (10’) after addition of latrunculin. Correlation coefficient R^2^ > 0.99 for all fits.

To follow up the finding that both *α* and *β* isoforms bind actin within filopodia, we examined the kinetics of fluorescence decay after photoactivation of PaGFP fusion constructs within the cell cortex. A brief flash of TIR laser light at 405 nm was used to activate PaGFP, and continuous illumination at 488 nm allowed the activated fluorescence to be visualized. The fluorescence of PaGFP alone decayed rapidly, reaching half its initial value within a single video frame (<50 ms; Fig. 4 *A*). The decay was two orders of magnitude more rapid than the photobleaching rate estimated from photobleaching of immobilized GFP molecules or photoactivation of fixed cells (see Materials and Methods). Therefore, the decay must reflect diffusion of the photoactivated PaGFP molecules out of the evanescent field.

The PaGFP-CaMKII fusion constructs (PaGFP-*α* and PaGFP-*β*) showed slower and more complex kinetics (Fig. 4 *A*), although it was still rapid relative to photobleaching. Their fluorescence decay could be followed by single-molecule tracking. The decay profiles were approximated by dual-exponential fits with a 0.24 s offset relative to the PaGFP intensity decay due to the five-frame lifetime tracking filter. Direct image field intensity measurements, analogous to those used for PaGFP but corrected for the offset, showed a twofold difference in the fast-component, but not the slow-component, decay. Tracks may terminate for reasons other than fluorescence loss, specifically crossover of tracks of unbound particles and imperfections of the tracking algorithm (Supporting Materials and Methods), that could account for the modest discrepancy.

The slow components for PaGFP-*α* (1.9 s) and PaGFP-*β* (3.0 s) were incompatible with free diffusion. Therefore, we used latrunculin B (latrunculin) (51) to test whether disruption of the actin cytoskeleton affected the mobility of PaGFP-CaMKII fusion proteins. The effect of latrunculin on HUVEC stress fibers was evident within a few minutes (Fig. 4 *B*). Before latrunculin treatment, PaGFP-*α* colocalized weakly, generating an anisotropic pattern that aligned with the stress-fiber arrays as revealed by the elliptical Fourier transform (FT) spectra of the red/green images (red FT (R_(maj/min)_ (major/minor axial ratio)) = 1.35, angle = 16° ± 5°); green FT (R_(maj/min)_ = 1.33, angle = 24° ± 5°). After incubation (10 min) with latrunculin, the pattern had disappeared (FT R_(maj/min)_ = ∼1 for both channels; Fig. 4 *B*, *insets*). We measured the photoactivated fluorescence decay kinetics at 0 and 10 min after latrunculin treatment. Dual-exponential fits to the fluorescence decrease showed that the amplitude and rate of the fast-decay component increased with time after treatment, consistent with a reduced F-actin-immobilized fraction (Fig. 4 *C*). We repeated the experiment with PaGFP-*β*. Photoactivated PaGFP-*β* formed brightly fluorescent filamentous substructures that disappeared after latrunculin treatment. The kinetics of PaGFP-*β* fluorescence decay also changed (Fig. 4 *D*) concomitantly with the observed structural changes. The fluorescence decay after photoactivation revealed a substantial fast-decay component for pulses applied 5 min after latrunculin treatment. The fast component increased with incubation time, so for photoactivation pulses 15 min after latrunculin treatment, the decay was similar to that seen for photoactivated PaGFP-*α* 10 min after latrunculin treatment. The fast-component decay was consistent with the formation of a PaGFP-like inert species.

### Structural determinants of the CaMKII F-actin interaction

**Figure 5:**
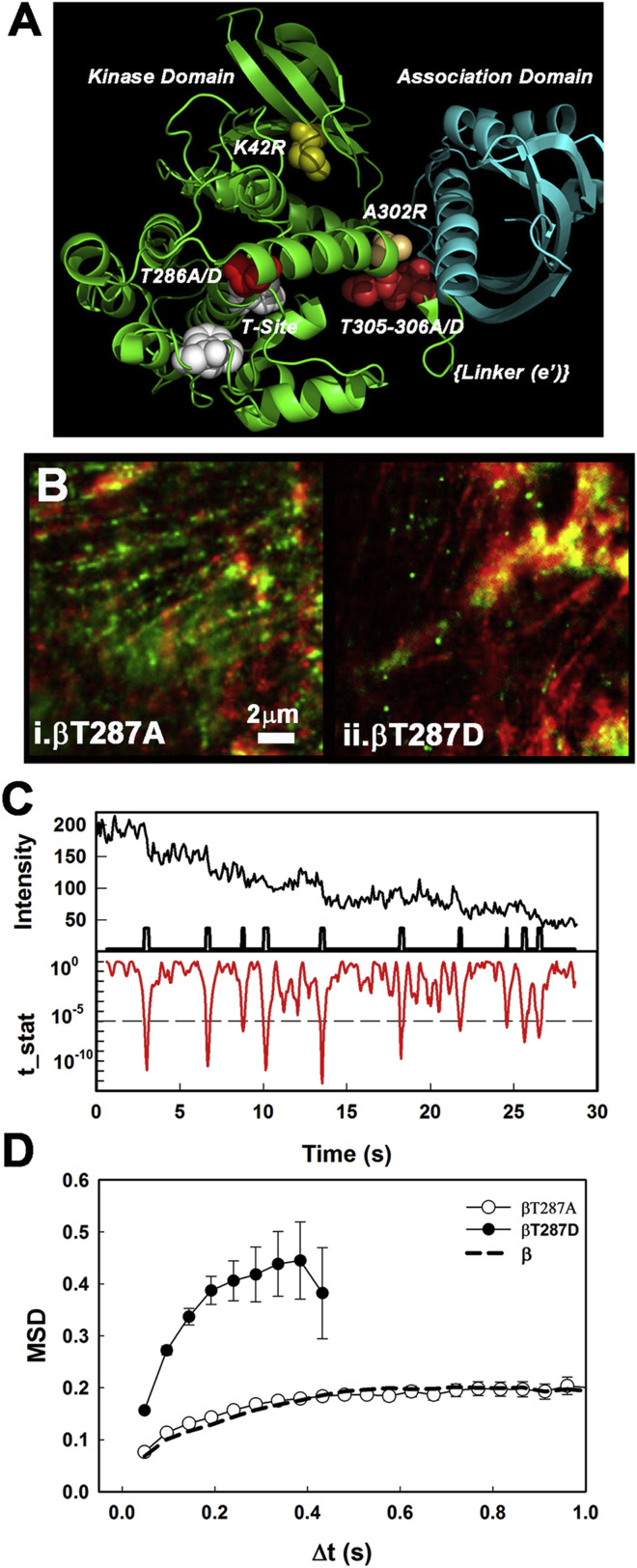
The T287D point mutation downregulates association with the actin cytoskeleton. (*A*) Atomic structure (PDB: 3SOA) of rat CaMKII (18). The positions of the mutated sites (K42 (*yellow*), T286 (*red*), A302 (*peach*), and T305/T306 (*magenta*)) studied; the junction of kinase (*green*) and association (*blue*) domains where the splice E′ linker segment would be located; and the substrate-binding T-site (*white*) are shown in relation to the secondary-structure elements (cartoon representation). (*B*) Superimposed averaged images, processed as in Fig. 3*A*, show localization of single molecules (Fig. S4; Movies S2 and S3) of (*i*) the dephosphorylated mimic, *β*287A (550 frames, *P*_pix_ = 0.29, *P*_rand_ = 0.16 ± 0.07, *n* = 6511) and (*ii*) the phosphomimic, *β*287D (475 frames (*P*_pix_ = 0.08, *P*_rand_ = 0.0 ± 0.08, *n* = 1020) with tRFP-actin (*red*, 100 frames). The mean tRFP intensities were 105 ± 6 (*β*T287A) and 213 ± 75 (*β*T287D) counts/pixel. (*C*) Photobleaching profile of a long-lived *β*287A-GFP track (*upper black line plot*) with the corresponding *t*-test statistic (*t*-stat) based on a rolling, nonoverlapping 12-frame window (*lower red line plot*). The *t*-stat axis denotes the probability that successive 12-frame segments have the same mean. The probability threshold was set to 10^−5^ for detection of a step change. The bars on the time axis of the upper plot mark the 10 steps identified by the *t*-test. The mean lifetime and intensity decrease per step were 2.6 ± 0.4 s and 15.5 counts/pixel, respectively. (*D*) Average track MSD-versus-Δ*t* plots for the *β*287D and *β*287A populations. The dashed line is the plot for the native *β* population redrawn from Fig. 3*C*.

Having established single-molecule imaging techniques using native *α* and *β* isoforms, we next examined the GFP fusions of a panel of functionally significant CaMKII mutants. The mutations are mapped onto the CaMKII structure in Fig. 5 *A* (residue positions are incremented by one in the corresponding *β* sequence). The primary phosphorylation site, *α*T286, is important for long-term depression (LTD) as well as LTP since these functions are impaired in <*α*T286A> mutant mice (52, 53) and are affected or abolished, respectively, by overexpression of a constitutively active<*α*T286D> (54). To explore its role in single-molecule binding to cytoskeletal actin, we studied the homologous *β*T287A and *β*T287D mutants (1). Phosphorylation of the secondary sites *α*T305 and *α*T306 is known to inhibit kinase activity (33). We compared differences among the *α*T286D/T305/T306 triple mutants with both secondary sites mutated to either aspartate or alanine. Other mutations/lesions of interest were *α*K42M, which blocks ATP binding necessary for CaMKII activation, LTP, and spine enlargement (55); *α*A302R, which disrupts calmodulin binding and translocation to the PSD (9); and the *β*E′ splice variant, which lacks linker sequences encoded by exons I and IV (56). Finally, we used the tatCN21 inhibitor, which competes with the NR2B NMDA receptor subunit for binding to the T-site (57), to see whether CaMKII binding targets elicit structural changes (58) that affect F-actin association.

#### The primary phosphorylation site mutants have dramatically different effects on F-actin binding

Averaged images show that the phosphorylation-incompetent *β*T287A mutant decorates cortical actin structures (Figs. 5 *B*(*i*) and S4). In contrast, the *β*T287D videos (Movies S2 and S3) show an isotropic distribution of fast-moving spots in the cell cortex that did not map onto the stress fibers (Figs. 5 *B*(*ii*) and S4). As for *β*, the tracks of immobilized *β*T287A spots have initial intensities that are several multiples of individual GFP fluorophores and show multistep photobleaching time courses. A rare example of a long-lived track reveals 10 steps (Fig. 5 *C*), consistent with the intensity ratio of the immobilized *β*T287A spot relative to single GFP fluorophores. In contrast, averaged images of the phosphomimetic *β*T287D mutant show no evidence of actin colocalization.

The difference between the two mutant proteins was emphasized by an analysis of MSD versus Δ*t* plots (Fig. 5 *D*). For *β*T287A, the initial slope and subsequent behavior were superimposable with results obtained using native CaMKII*β*. The addition of tatCN21 (1 *μ*M) had no effect on the association of *β*T287A with F-actin (two different cultures, >10,000 tracks). In contrast, the initial (MSD versus Δ*t*) slope for *β*T287D was much greater than that for *β* and *β*T287A, with virtually no (<7%) tracks of duration longer than 0.4s (+0.24 s offset), consistent with fast-moving objects that diffused rapidly out of the evanescent field.

#### All of the mutant isoforms showed similar single-object intensities but formed two distinct mobility groups

**Figure 6:**
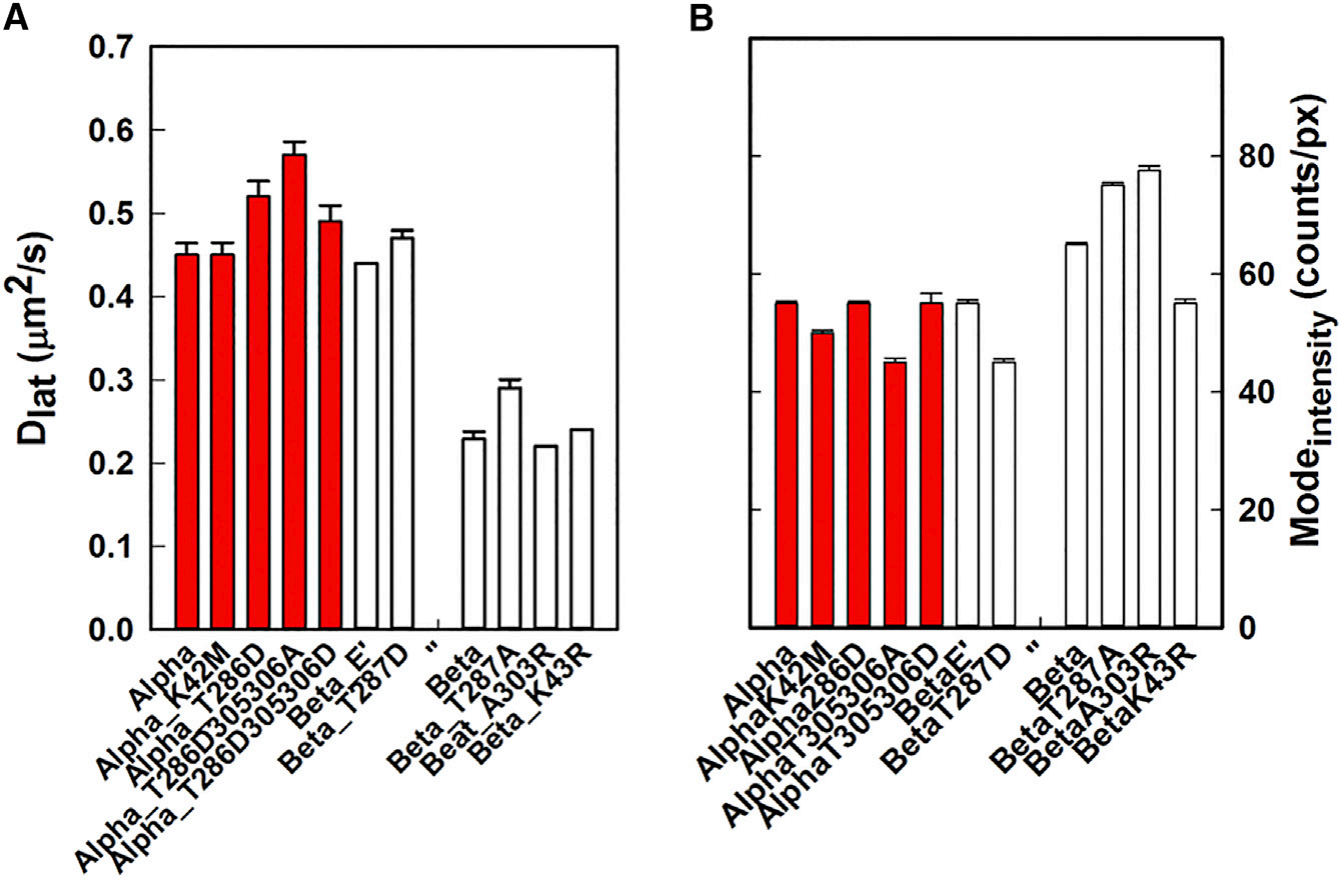
Characterization of the mutant proteins. (*A*) Mobility (*D*_lat_ (mean ± SE)) values for the protein populations. Red bars indicate *α*-isoforms; white bars indicate *β*-isoforms. *β*T287D and *β*E′ have mobility similar to that of the *α* proteins. (*B*) Mode (± SE) intensities for the native and mutant GFP-CaMKII fusion protein populations. The bar colors indicate isoforms as in (*A*).

ANOVA was used to test for significant differences in $D_{lat}$ values for the panel of CaMKII mutants (Fig. 6 *A*) based on estimates of variance within and between data sets (Table S1). Variances were normalized for different degrees of freedom, and the probability, *p*, that differences between populations were significant (*p* < 0.05) was computed. Consistent with a visual inspection of the data (Fig. 6 *A*), the results showed two distinct groups: a low-mobility group comprised of the *β* proteins (native *β*, *β*K43R, *β*A303R, and *β*T287A) and a high-mobility group comprised of all the *α* isoform mutants together with *β*T287D and *β*E′. A similar pattern was obtained when instantaneous velocities were compared (Fig. S5). The modal, single-spot intensity values (Fig. 6 *B*) obtained across all proteins are similar and vary between 50 and 80 counts/pixel, which is four- to sixfold lower than the anticipated value for the CaMKII holoenzyme and twofold greater than for single GFP fluorophores. The similar values rule out oligomer aggregation as a possible cause of the mobility differences between species. The brighter spots seen decorating stress fibers in some video frames are due to PSF overlap of closely opposed spots; however, their tracks can be separated provided the spots are not stationary (27). Spot intensity measurements suggest that the expression level affects only the holoenzyme number and not the subunit stoichiometry (Fig. S5 *B*). Disassembly is also not the cause of the interspecies mobility differences, since the distributions lack peaks for the single GFP intensity and monomeric *α* could not be tracked (see “Visualization of homomeric GFP-CaMKII*β* complexes in the cellular cortex” above). The intensity histograms of *β*, *β*T287D, and *α* (Fig. S5 *C*) are differentiated by their skewness rather than their modes. The skewness reflects long-lived track lifetimes and results from oligomer immobilization on actin stress fibers (as shown in Fig. 3, *A* and *B*).

#### CaMKII dissociation from cytoskeletal actin

Thus far, our analysis indicates that the track lifetimes for both *β* and *α* are biphasic, with MSD versus Δ*t* plots of the short-lived population being consistent with diffusion out of the evanescent field (e.g., Figs. 3 *B* and 5 *D*). The photoactivation experiments in the presence and absence of latrunculin demonstrate that the population track lifetime is dramatically reduced coincidently with stress-fiber disassembly. The reduction is mainly due to loss of the long-lived population, implying that these population lifetimes are limited by dissociation from the actin cytoskeleton (Fig. 4). With this in mind, we used track lifetime histograms to estimate the bound fraction and the F-actin dissociation rate for different CaMKII mutants.

The lifetime of the phosphomimic *β*T287D was taken as representative of unbound molecules, based on *β*T287D’s failure to decorate cytoskeletal structures (Fig. 5 *B*; Movie S3) and its high mobility (Fig. 6 *A*). Consistent with this idea, the *β*T287D track lifetime data were also fairly monotonic with single exponential decay (rate constant = 6.85 s^−1^ (1–0.05, R^2^ = 0.99); Fig. S6). We then fitted all of the other track distributions over this range to a function that assumed there was a nonbinding fraction (i.e., like *β*T287D) and another longer-lived fraction (A_o_) that represented actin-binding complexes with an unknown but slower dissociation rate (*k*):

(3) $$A_{t}=A_{0}\left(e^{-kt}\right)+\left(1-A_{0}\right)\left(e^{-6.85t}\right).$$

The cytoskeletal actin content was assumed to be the same for all experiments, consistent with the modest variation (127 ± 49 counts/pixel) in the mean tRFP-actin intensities in the images (Figs. 3, 4, and 5). The additional information obtained from Eq. 3 is the estimate of the bound ($A_{0}$) to freely diffusing pools ($1-A_{0}$) of molecules and of the dissociation rate, k, of molecules from the actin cytoskeleton. The bound fraction, *A*_o_, was 0.22 ± 0.02 for all strong-binding *β* fusion proteins (minus *β*E′). *A*_0_ was ∼2-fold lower for (*α*) proteins. The overall group pattern was similar to the pattern observed in the *D*_lat_ analysis. The k-values were 2.9 s^−1^ and 1.3 s^−1^, respectively, for native (*α*) and (*β*) (Fig. S6).

Equation 3 would be valid over the complete (1–0) range only for homogeneous populations that follow single-parameter Poisson probability time distributions. This is not the case for the two populations. For the unbound population, as represented by the *β*T287D proteins, the *D*_lat_ value for the most mobile among them (∼0.5 *μ*m^2^/s; Fig. 6 *A*) was ∼18-fold lower than the *D*_Stokes_ value calculated for free-diffusing (*β*) holoenzymes (∼10 *μ*m^2^/s; Eq. 1). This discrepancy, as well as the deviation of the *β*T287D distribution from the single exponential fit (Fig. S4), indicates hindered diffusion, although bias introduced by exclusion of rapidly diffusing objects by the five-frame (0.24 s) track filter would also contribute. Power-law distributions due to hindered diffusion have been characterized for F-actin gels in vitro (59), as well as in vivo for membrane proteins confined by the actin cortex (60, 61). The tRFP-actin labeling does not resolve F-actin single filaments in the dense cortex or F-actin spacing in stress fibers, but limits on physical entrapment may be estimated (Supporting Materials and Methods) to rule out this scenario for stress-fiber decoration. For the bound population, a single k will obtain only if the dissociation of CaMKII from F-actin subunits does not depend on neighboring subunits. This is not the case, since the detachment probability of a subunit will be lower if neighboring subunits participate in binding together the CaMKII holoenzymes and F-actin.

**Figure 7:**
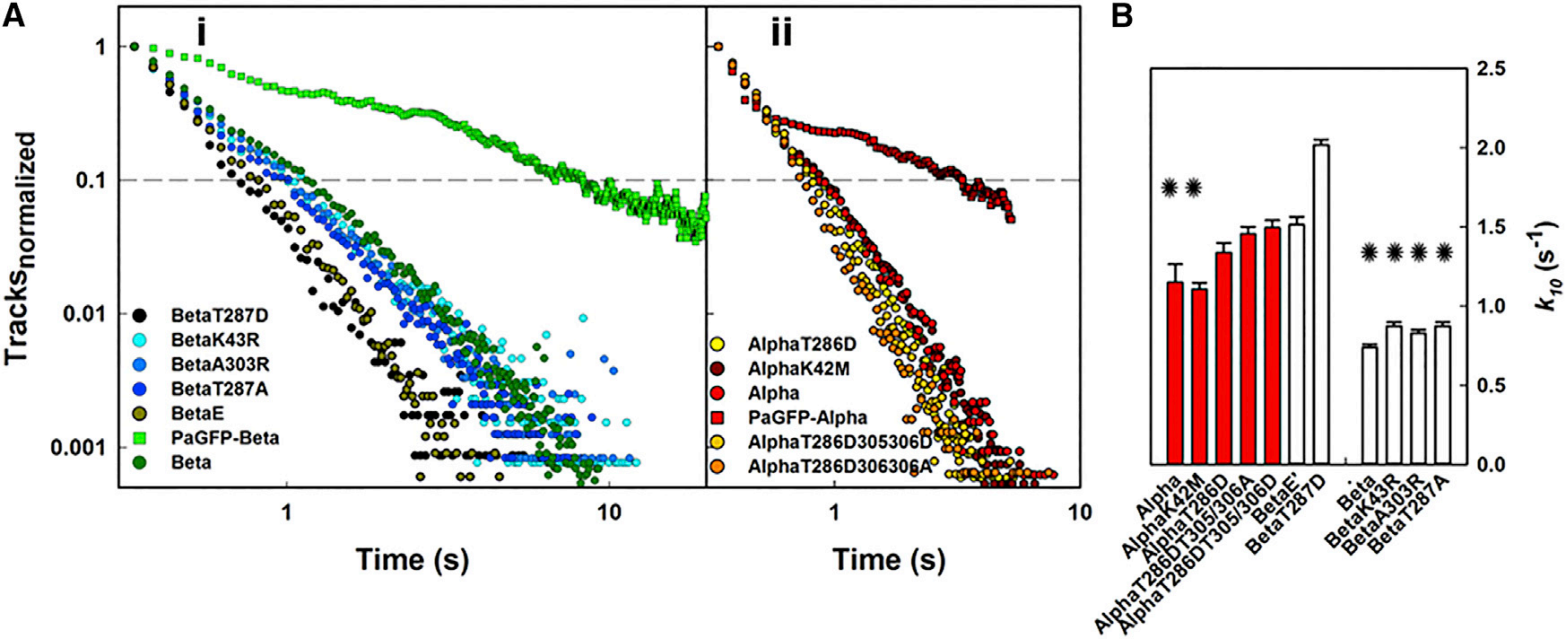
Track lifetimes show log-normal decay. (*A*) Log-log plots of the CaMKII track lifetime distributions deviate from dual exponential fits and show a downward curvature that is most evident at longer times (*i*, *β* proteins; *ii*, *α* proteins). (*B*) Histogram of rates (*k*_10_) computed from decay times to 10% amplitude. Asterisks mark species that associate with F-actin.

Therefore, we replotted all of our data on log-log axes. We found that they deviated markedly from a dual-exponential process once the population fraction was <5% (Fig. 7 *A*). All distributions showed the same convex log-log relation, consistent with a multiexponential, log-normal distribution of dissociation times. The initial phase of the log plots over which Eq. 3 is valid provides important estimates of the major binding modes. Nevertheless, it was clear that at longer times the data deviated from a single-parameter, two-population model, and this observation was consistent across all data sets.

We compared the times required to reach 10% of the initial amplitude (*t*_1/10_) between data sets (Fig. 7 *B*) to better represent the log relations. We found the same grouping of different mutants as observed in the *D*_lat_ analysis. ANOVA (Table S2.I) did not reveal significant differences between the grouped (*α*) proteins, but did so when these were grouped with *β*T287D, *β*E′.

As expected, *t*_1/10_ was lowest for *β*T287D (1.14 + 0.02 s), the reference unbound state, and highest for native *β* (3.1 ± 0.08 s). We further analyzed differences between the data sets by conducting pairwise *t*-tests against the *β*T287D reference (Table S2.II) to parse out differences between group members that were not revealed by the ANOVA. The *t*-tests revealed *α* and *α*K2M as outliers within the weak-binding group, whereas the *t*_1/10_ values measured for *β*E′ and the *α*T286D proteins with and without secondary phosphorylation site mutations were not significantly different from those obtained for *β*T287D (Fig. 7 *B*).

We used the photoactivation data to estimate the dissociation constants (K_D_)^app^ of CaMKII for actin. These data provide a more valid estimate of the actin dissociation rate, *k*_off_, since locally activated PaGFP-tagged molecules essentially only leave the evanescent field, whereas GFP-tagged molecules can both exit and enter from the bulk cytoplasm (Supporting Materials and Methods), resulting in a sevenfold difference in the observed decay (Fig. 7). Our *t*_1/10_ decay rates for PaGFP-*α* (3.4 ± 0.4 s) and PaGFP-*β* (9.4 + 0.2 s) give *k*_off_ (= *k*_10_ ((log (10))/*t*_1/10_) estimates of 0.68 s^−1^ and 0.23 s^−1^, respectively. If we assume that the rate of actin association (*k*_on_) is in the middle (5× 10^5^ M^−1^s^−1^) of the narrow (10^5^–10^6^ M^−1^s^−1^) diffusion-controlled range (62) applicable to high-ionic strength media such as cell cytoplasm (63), (K_D_)^app^ (= *k*_off_/*k*_on_) is 0.5 *μ*M for *β* and 1.4 *μ*M for *α*. The estimate for *β* is comparable to its measured 2.4 *μ*M affinity for G-actin (24). It is consistent with the simplest scenario of a common binding surface for both G- and F-actin, although more complex scenarios are possible (64, 65).

## Discussion

In this work, we used TIRFM-based single-molecule imaging experiments, based on dual-color and photoactivation techniques, to study the dynamics of the interactions of CaMKII isotypes with F-actin within live cultured cells. Our ability to detect micromolar-affinity, weak-binding interactions at subsecond resolution provides information that complements classical sedimentation and gel chromatography assays, and leads to important new, to our knowledge, insights.

### CaMKII binding to cytoskeletal actin

We conducted mutant analyses to characterize CaMKII binding to cytoskeletal actin. Substitution of the primary phosphorylated threonine residue by aspartate (*β*T287D and *α*T286D) abolished F-actin association for both isoforms, whereas substitution with alanine had no effect. The *α*T286D mutation abrogated affinity for actin and this effect was independent of mutations at the secondary phosphorylation sites. The *β*T287A and *β*T287D data are consistent with the idea that primary-site phosphorylation acts as a single-stage toggle switch, in line with some activation scenarios (66), to control F-actin binding affinity. Consistent with this idea, the *β*K43R and *α*K42M mutations that abolish ATP binding had no effect on F-actin association. Both *β* and *α* isoforms should bind ATP in HUVECs, since the CaMKII Michaelis constant for ATP is ∼40 *μ*M (58) and the cytoplasmic ATP concentration is typically 2–5 mM (67). In addition, the *β*K43R/*α*K42M data show that, in contrast to association with the receptor subunit GluN2B (68), the association with CaMKII F-actin is insensitive to ATP binding and subsequent hydrolysis per se. Elimination of calmodulin binding by the *β*A303R mutation or use of the peptide inhibitor tatCN21 had no effect on F-actin association. These observations are most simply consistent with low basal Ca^2+^ and CaMKII activity within HUVECs. Finally, the splice segment that is absent in *β*E′ is essential for actin binding by the *β* isoform, consistent with sedimentation assays (22).

Using single-molecule live-cell imaging, we built upon the initial report of stress-fiber decoration in fixed cells (20), which established the CaMKII-F-actin interaction. Our direct observation of actin stress-fiber decoration by immobilized GFP-CaMKII holoenzymes in the presence of a mobile background fraction is consistent with specific binding to F-actin and is incompatible with nonspecific entrapment based on the known stress-fiber architecture. This is also the case for mobility distributions in other actin-rich regions of the cell, based on the known cortical F-actin density and calculated filament mesh size (Supporting Materials and Methods). We found no evidence for higher-order clustering of holoenzymes into larger aggregates that could become either entrapped within or excluded from the actin cortical network or stress fibers. The multistep photobleaching behavior of static spots, along with our histogram analysis of single-object fluorescence intensities, establishes that holoenzymes of CaMKII were the predominant species analyzed in our assays. The presence of larger aggregates is further ruled out by the fact that mutations that abolish Ca^2+^/CaM binding (A303R) or nucleotide binding (K42M/K43R) required for aggregate formation (36, 69) did not alter the native *α*/*β* mobility and lifetime distributions. Thus, the mobility differences between the *β*287D/*α*T286D proteins and other CaMKII species, as well as the differences between weak- and strong-binding groups analyzed in this study, can only be explained by differences in F-actin binding affinity.

Although the possibility of CaMKII association with other stress-fiber actin-binding proteins (ABPs) cannot be eliminated, three considerations argue for direct association with F-actin. First, the ABP would need to be abundantly and uniformly distributed along the fibers to be consistent with our images (Figs. 3 *A* and 4 *A*; Movies S1 and S2). Other stress-fiber structural ABPs (*α*-actinin and nonmuscle myosin II) display periodic banding (42). CaMKII binds to *α*-actinin (70), but this binding is not affected by primary-site phosphorylation (71) and thus may be ruled out. Second, although activated CaMKII has multiple binding targets, there are few binding partners for inactive CaMKII (2), which, as argued above, may be the dominant form in our HUVEC cultures. Third, the relative binding strengths of the CaMKII*β* mutant proteins in our measurements correlate well with results obtained with synthetic F-actin filaments in bundling assays (22).

In neuronal cultures, differences in dendritic arborization (31) and mobility (32) between native and mutant (A303R and K43R) *β* GFP fusion proteins have been reported, but these differences are probably due to spontaneous neuronal activity that triggers Ca^2+^.CAM binding for CaMKII activation. Hence, although we do not think that multiple binding partners play a role in our assays, they likely do so in dendritic spines. The reported multiple CaMKII kinetic spine subpopulations (16) may also be due, in part, to multimodal interactions with the F-actin network documented in this study.

### Mechanisms for the log-normal bound lifetime distribution

The estimated dissociation constants, (K_D_)^app^, for actin are on the order of micromolar for both isoforms. The weak (micromolar) binding of the major *β* mode is in the ballpark of its reported G-actin affinity. The log-normal distribution of track lifetimes indicates that stronger binding modes exist in addition to the dominant initial mode. These modes may arise from engagement of a variable number of CaMKII subunits with one or more actin filaments (Fig. S7). The fact that the log-normal relation holds for both isoforms and their mutant variants is consistent with the notion that both have a common F-actin binding determinant that is more accessible in *β* due to its longer, more flexible linker. The alternatively spliced linker region encoded by exons I and IV may increase flexibility between subunits comprising the multimeric complex, thereby ameliorating the geometrical mismatch between CaMKII subunits (72) and binding sites on actin (73). The increased flexibility would optimize contact at the CaMKII and F-actin binding interface and facilitate simultaneous binding at two or more sites, thereby increasing binding avidity. This flexibility could also contribute to heterogeneous binding kinetics, as single-molecule studies indicate that proteins may exist as fluctuating conformational ensembles that lead to power-law distributions in enzyme-turnover experiments over the 10^−3^ to 10 s timescale (74). Phosphorylation or substitution of serine/threonine residues within the linker may also attenuate flexibility, based on differences in residue size and charge, to regulate persistent CaMKII association with F-actin (13). The possibility that the two isoforms have distinct binding determinants for F-actin cannot be ruled out, but we favor the idea of a common determinant as an explanation based on linker length, which would also account for the difference seen between *β* and *β*E′.

### Physiological implications of the log-normal binding curve

The log-normal binding curve extends the concentration range for interaction with the actin cytoskeleton. It has two consequences, as described below:

First, it explains why the temporal resolution of the assay determines the detection sensitivity (Fig. S7). Our ability to detect weak-binding species will decrease as the time resolution of the assay increases. The likely explanation for the inability of classical assays to resolve the weak F-actin binding of <*α*> is that they only detect long-lived, tight-binding states. The binding of <*α*> to F-actin, thus established, must have a physiological rationale. Variations in isoform expression ratios occur as neurons develop. The binding ensures that CaMKII holoenzymes, predominantly composed of *α* subunits, can also target the actin cytoskeleton. Although the noted functional effects of *α*T286D mutation are thought to be mediated by *α* kinase activity, F-actin association may also play a role. <*α*T286D> has reduced synaptic localization (75, 76) even though it binds GluN2B in vitro, and loss of F-actin association could account for this effect.

Second, the curve has implications for the transport of <*β*> down neuronal processes. in contrast to <*α*>, <*β*> is expressed only in neuronal cell bodies. Increased avidity due to binding of multiple subunits to F-actin would depend on both the concentration and geometry of F-actin, as well as the multimeric state and flexibility of CaMKII. We estimate expression levels of 0.2–0.4 *μ*M in our assays, based on the density of fluorescent spots in the videos (2000/20 *μ*m^2^ image field area) and the 100 nm effective depth of the evanescent field. CaMKII concentrations in neurons are severalfold higher: above ∼10 *μ*M holoenzyme in dendritic spines and ∼2–5 *μ*M holoenzyme in dendritic processes (5). In regions of the cell where the actin cytoskeleton is sparse (i.e., dendritic and axonal branches (77)), the low (micromolar) affinity of monovalent <*β*> binding would minimize its association with cytoskeletal actin during transport along the long neuronal processes (78). However, in regions where the actin cytoskeleton forms a dense three-dimensional meshwork (i.e., dendritic spines), binding via multiple subunits would be favored and <*β*> would be immobilized. Thus, the extended binding range would facilitate unhindered transport of CaMKII along neuronal processes and sequestration at dendritic spines.

Within dendritic spines, the avidity difference between the two isoforms and between <*β*> and <*β*E′> would increase, with qualitatively different effects on the spine actin cytoskeleton. High avidity mediated by a few *β* subunits in the *αβ* hetero-oligomers might be sufficient to stabilize the dynamic actin cytoskeleton. The <*β*E′> splice variant is expressed in immature neurons (21, 31) when affinity for F-actin, which is not required for structural remodeling of synaptic sites, would only hinder the transport needed for targeted kinase activity. Our results provide a quantitative rationale for the fact that the expression of <*β*E′> has different physiological effects compared with that of <*β*>.

In conclusion, using single-molecule assays, we were able to resolve CaMKII F-actin binding events on the subsecond-to-second timescale in live mammalian cells. We documented the binding of both neuronal CaMKII isoforms and measured the effect of mutations that act at different points in the CaMKII activation cycle. The behavior of the mutants establishes that binding of CaMKII to actin only occurs when CaMKII is inactive (specifically, when it is not phosphorylated at the primary phosphorylation site). This is in contrast to binding of CaMKII to GluNB, which is triggered only when the kinase is active. This new, to our knowledge, information should be valuable for modeling the role of the actin cytoskeleton in CaMKII transport and synaptic localization.

## Author Contributions

S.K. designed and performed research, analyzed data, and wrote the manuscript. I.C. performed research. T.C. contributed reagents. K.U.B. designed research, contributed reagents, and wrote the manuscript. J.E.M. designed research, analyzed data, and wrote the manuscript.

## Supporting Citations

References (79, 80, 81, 82, 83, 84, 85) appear in the Supporting Material.

## Supporting Material

Document S1. Supporting Materials and Methods, Figs. S1–S7, and Tables S1 and S2

Movie S1. Video Recording for Fig. 3 *A* The first section of the video shows tRFP-actin (*red*) and gfp-CaMKII-*β* (*green*), played back in real time; the middle section shows the averaged intensity for both channels overlaid as a static image; and the final section shows a section of the separate averaged images after local background subtraction. Colocalization of *β* and F-actin is seen at actin stress fibers (*long white arrows*) as well as in regions that are expected to be rich in F-actin (membrane ruffles, *short yellow arrows*). CaMKII/stress-fiber colocalization was quantified.

Movie S2. Video Recording for Fig. 5 *B*(*i*) The first section of the video shows tRFP-actin (*red*) and βT287A (*green*), played back in real time, and the middle section shows the averaged intensity data for both channels overlaid as a static image. The single-particle tracks are overlaid and finally the averaged tRFP-actin image is shown.

Movie S3.Video Recording for Fig. 5 *B*(*ii*) The first section of the video shows tRFP-actin (*red*) and βT287D (*green*), played back in real time, and the middle section shows the averaged intensity data for both channels overlaid as a static image. The single-particle tracks are overlaid and finally the averaged tRFP-actin image is shown.

Document S2. Article plus Supporting Material
