# Supplementary material for: Multiple CaMKII Binding Modes to the Actin Cytoskeleton Revealed by Single-Molecule Imaging
Source: Biophys J. 2016 Jul 26;111(2):395–408. doi: 10.1016/j.bpj.2016.06.007 (PMC4968397; doi:10.1016/j.bpj.2016.06.007)
Supplement: Document S1. Supporting Materials and Methods, Figs. S1–S7, and Tables S1 and S2 [file mmc1.pdf]

Biophysical Journal, Volume *111*

Supplemental Information

Multiple CaMKII Binding Modes to the Actin Cytoskeleton Revealed by Single-Molecule Imaging

Shahid Khan, Ianina Conte, Tom Carter, K. Ulrich Bayer, and Justin E. Molloy

SUPPORTING MATERIAL

A. Assay Development

(1) Total Internal Reflection Fluorescence Microscopy (TIRFM) – The microscope workstation was mounted on a vibration isolation table (*Isostation*, Newport Spectra-Physics Ltd., Didcot, UK). Laboratory and instrument temperature was maintained at 23.5^{o}C.

Stage movement in x,y planes and objective focus in the z-direction was effected with nanometer precision by piezoelectric positioners (*MCS-3D*, SmarAct GmbH, Oldenburg, Germany and *PIfoc P-721.10*, Physik Instrumente Ltd, Bedford, UK, respectively). The workstation was equipped with four lasers (*Crystal Laser CL-2000* (50mW @ 405nm) Reno NV, USA; *Protera A488-15*, (30mW @ 488nm) Laser2000, Huntingdon, Cambridge, UK; *DPGL-2200*, (200mW @ 532nm) Suwtech, Shanghai, China; *MGL-FN-561*, (200mW @ 561nm) Changchun New Industries Co. Ltd., Changchun, China). The laser beams were co-aligned and bought into total internal reflection (TIR) mode by a system of mirrors and lenses mounted on a combination of translation mounts and kinematic mounts (Thorlabs Inc., Newton NJ, USA). Laser intensities were modulated by variable neutral density filters (*NDC50C2M*, Thorlabs Inc.) or by varying the laser supply current. Power output was measured and calibrated using a laser power meter (*LaserMate-Q*, Coherent, Santa Clara, CA, USA). Laser powers were measured at the point where the laser beam exited the final objective lens. Lasers were blocked either by TTL modulation of the laser power supplies or by electronic shutter (Uniblitz D122, Vincent Associates, Rochester NY, USA).

Evanescent field excitation was achieved by focusing the laser beams at the extreme edge of the back aperture of a high-numerical aperture objective lens (*PlanApo 100x, 1.49NA*, Olympus, Southend-on-Sea, UK) to produce a collimated beam of light that emerged from the objective front face at an angle that exceeded the critical angle (>63°). Index-matched immersion oil (*Immersol 518*, Zeiss, Cambridge, UK) coupled the objective lens to the glass-bottomed culture dish (Lab-Tek, Nunc, Rochester, NY, USA). Filter cubes, mounted in the microscope's epifluorescence filter wheel, separated the eGFP fluorescence (Excite 488nm Laser, dichroic: Di02-R488; Emission filter: FF02-520/28, Semrock, Rochester, NY, USA) from the tagRFP fluorescence (Excite 561nm laser, dichroic: Di02-R561; Emission filter: FF01-609/54, Semrock).

For photo-activation studies, cells were co-transfected with tRFP-actin and a PaGFP-CAMKII fusion protein. Control cells were transfected with PaGFP. Transfected cells were first located by visualization based on their tRFP-actin fluorescence. Photo-activation occurred within one video frame and then increased during the duration of the flash, determined by a balance between the rate at which fluorophores were generated by photo-activation and lost by diffusion from the evanescent field and by photo-bleaching. Fluorophore excitation in the z-axis is determined by the evanescent field decay distance (1/e ~ 100 nm). Consistent with this interpretation, termination of the photo-activation flashes resulted in a rapid decay of fluorescence signal back to the starting level. Fluorescence changes were averaged over several cycles to obtain the rise and decay kinetics. For each genetic construct the mean decay profile was obtained by pooling video-records from different experiments (n >= 2). Photo-bleaching rate determined from photo-activation of a cell culture fixed with 1% paraformaldehyde was < 10% in 10 seconds. PaGFP and GFP photo-bleaching rates are similar (1).

The sample fluorescence was imaged by an electron-multiplying, EMCCD, camera (iXon-897bv, Andor, Belfast, UK) camera at 96 nm/pixel magnification and 20 fps readout of the full image field (512x512 pixels). Two-fold higher frame rates were achieved when desired by reducing the pixel readout area. Input to the laser shutters and image acquisition were controlled by a personal computer with custom software. Video data was stored on computer hard disc. Images were subsequently analyzed using either GMimPro (2) or ImageJ (3).

Live cells are more challenging than fixed cells for co-localization analysis. In fixed cells movements of cellular sub-structures or loosely-attached membrane relative to the glass coverslip

are not issues. Loss of soluble contents during fixation eliminates the high background in the red channel due to G-actin and labile F-actin microfilaments. In the TIRFM images, spatial differences due to cell body versus periphery, inhomogeneous cell attachment to the cover-glass and inexact coincidence of the red / green laser beam profiles will additionally be aggravated by the high camera gain required for single-molecule tracking. In addition, stress fiber labeling by RFP-actin (red channel) is punctate due to its low expression level compared to endogenous actin, while single molecule imaging (green channel) is inherently stochastic. Co-localization of red / green channel pixel intensities from selected quadrants, reported as the Pearson's correlation coefficient (P_{pix}), was computed using the Image J Manders coefficient plugin. The images were background subtracted and quadrants chosen to maximize the signal from the stress fiber array and minimize noise due to spatial inhomogeneity. Scatter plots gave an objective sense of co-localized pixel frequencies relative to non-overlapping pixels, as described (4). To assess significance the green (GFP) image was scrambled by shuffling pixel blocks to generate 25 randomized images. P_{pix} was then compared against the mean ($\pm\sigma$) correlation (P_{rand}) of the randomized images with the red (tRFP-actin) image (5).

(2) Single Molecule Image Analysis - The point spread function (PSF) of a single fluorophore in the object plane (here termed the x,y-plane), has a full-width at half-maximum (FWHM) of ~ 0.3 µm. At the 0.1 µm / pixel magnification of our imaging system, 95% of the PSF intensity is encompassed by a 3x3 square pixel region. An automatic single particle tracking (ASPT) algorithm, described previously (2) was used to detect fluorescent spots in successive video images based on convolution of a moving kernel of an idealized 2-dimensional PSF. Fluorescent spots, identified on the basis of their match to the idealized PSF are reinforced relative to other regions of the image. Following spot detection, the intensity of the spot was computed by taking the mean intensity of the pixels within the 3x3 pixel region surrounding the spot centroid less the intensity of the adjacent ring of pixels. The latter operation gave a local back-ground subtraction. The detected spot x-y coordinates were then determined with sub-pixel resolution by fitting the PSF to a 2-D Gaussian function. Spot locations were then tracked in successive video frames by linking object coordinates using a nearest-neighbor algorithm, to give object "tracks" consisting of a series of x-y coordinates, time and intensity. An intensity cut-off eliminated spots whose brightness was 20X that expected for single GFP fluorophores. A one-frame look up function minimized fragmentation of a trajectory of a fluorescent spot into multiple tracks. Fragmentation could be due to frame-by-frame displacements larger than allowed by the search area chosen to assign centroids in adjacent frames or to electronic camera noise. The object tracks were then further analyzed using either custom software, Excel (Microsoft Corp, Redmond, WA), IgorPro (Wavemetrics, Lake Oswego, OR, USA) or MatLab (Mathworks, Cambridge, Cambs, UK). Least squares fits based on the Kolmogorov-Smirnov normality test were performed in Sigmaplot 12.0 (Systat software, San Jose, CA, USA).

Our estimation of the lateral diffusion coefficient, D_{lat}, is derived from the initial gradient of mean squared displacement (MSD) versus time interval (Δt) plots. This assumes MSD increases linearly with Δt (i.e. the objects exhibit "free-diffusion", characterized by a Brownian walk). Although this assumption is not strictly valid, the estimated D_{lat} has comparative value as long as any anomalous behavior scales linearly, which seems a fair approximation. Although mobility at the plasma membrane is essentially constrained in two-dimensions, diffusion within the cytosol is three-dimensional and can be estimated from the two-dimensional projection of the object's motion, applying a linear, $2/3^{rds}$ scaling factor. The averaged particle speeds, V_{avg}, are normally distributed (as expected, from the central limit theorem).

(3) In situ photo-bleaching of immobilized tracks – The ability to record bound lifetimes of single GFP-CaMKII holoenzymes rests on the fact that photo-bleaching of multiple fluorophores takes many seconds. T-tests (**Fig. S1**) show that GFP-CaMKII intensity jumps (Figure 3D) have similar frequency

but smaller fractional intensity drops per step (Results Section A4) in comparison to tracks of immobilized GFP molecules (Figure 2B),.

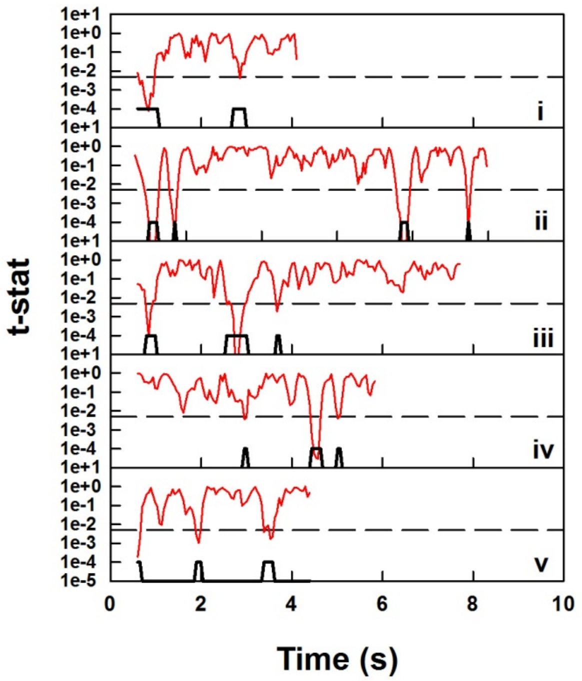

Fig. S1: Step photo-bleaching - *T-test statistics (red lines) for the track intensity profiles in Figure 3D. The t-test statistic records the probability that the mean intensities of adjacent twelve frame segments were the same. Intensity jumps (black markers) were identified when the probability was <* $10^{-2.5}$ *(dashed lines).*

(4) The relation between intensity, D_{lat} and track lifetimes – The intensity, duration and mobility of six single tracks, selected from the population distribution studied in Figure 3, were analysed (**Fig. S2A**). The frame centroid intensity determined from the tracking algorithm, is computed by local background subtraction. It varies inversely with the frame-to-frame displacement being lower when molecules are unbound and their fluorescence is distributed over a larger area due to diffusion. The form of the population-averaged MSD-Δt plot reconstructed from plots of the individual tracks is governed by differences in track lifetimes. At low Δt values it is governed by a mean D_{lat} representative of the mobility of all spots. At high Δt values, its form is governed by the decrease in population size as the more mobile spots exit the evanescent field and the immobile, long-lived spots dominate as illustrated by the six-track population plot.

The complete, population distribution is shown (**Fig. S2B**) to identify how the sub-populations (Figures 3B, C) were selected based on their lifetimes. There will be a continuum between the mobility of unbound versus tightly bound particles if population size is sufficiently large (> 10^3). The population curves (Figures 3B, 5D) for the short lived tracks (Fig. S2B yellow bar) and the complete population may be reconstructed based on simple equations, to a good approximation, to sharpen intuition.

For short tracks,

$$\overline{MSD_{t=1}^{n}} = 4\{\textstyle\sum_{t=1}^{n}(Tracks.D_{lat})/\sum_{t=1}^{n}Tracks\}.t \qquad \textbf{A}$$

For the complete population

$$\overline{MSD_{t=1}^{n}} = 4\{(\sum Tracks).\overline{D_{lat}} - \sum_{t=0}^{n-1}(Tracks.D_{lat})\}/\{(\sum Tracks) - \sum_{t=0}^{n-1} Tracks\}. \quad \mathbf{B}$$

The population curve for the short tracks generated by equation-A mirrors the curve shown in Figure 3B well as the plots of the individual tracks are close to the linear MSD versus Δt relation assumed in the equations. This is not the case for the long-lived tracks as seen from tracks 2 and 4 (Fig. S2A). The deviation from the assumption results in a discrepancy in the rise and decay amplitudes between the real curve and the curves generated by equation-B. Nevertheless the basic characteristics of the initial rise, peak and subsequent decay are reproduced.

Kymographs of molecules confined in filopodia (**Fig. S3**) provide visual illustration of the relation between intensity and D_{lat} for single tracks constrained to move in the (x, y) plane. The mean intensity of the kymograph tracks $Q = \Sigma q/N$ where N is the number of frames.

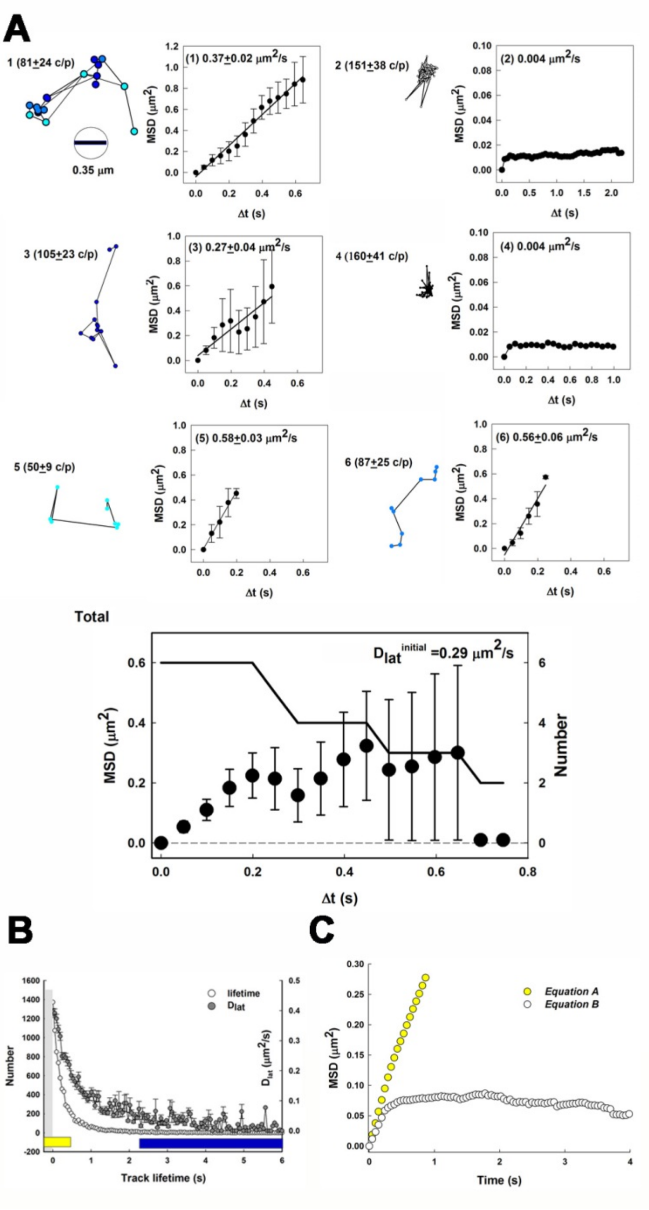

Fig. S2: Properties of single tracks and their population MSD-Δt plot –A. *Panels show single tracks linking centroids in successive frames (left) and the resulting MSD plots (right). Centroid colour reflects intensity (cyan (40-65 counts / pixel), blue (70 – 95 counts / pixel), dark-blue (100-125 counts / pixel), black (140-165 counts / pixel)). Mean track intensities and derived D_{lat} values are listed. Error*

bars for single track MSD plots were computed following (6). Error bars for the population plot (black symbols) reflect the variation in population size. Dips in the plot mirror the decreases in the number of tracks (solid line). At $\Delta t = 0.7$ s, MSD drops to the value (0.004 μm^2) for the 2 immobilized tracks. The value reflects the positional uncertainty in centroid determination. ***B.*** *The decrease in the size of the complete population (Figure 3) with time. Associated changes in D_{lat} ($\pm$ σ) values are shown as a histogram, binned according to lifetime. Grey vertical bar is 5-frame lifetime track filter The Figure 3B plots for the selected sub-populations (yellow (short tracks); blue (long-tracks)) illustrate the D_{lat} changes.* ***C.*** *MSD-Δt plots generated from the population histogram (B) by equation A (short-lived tracks (yellow)) and equation B (complete population (white))*

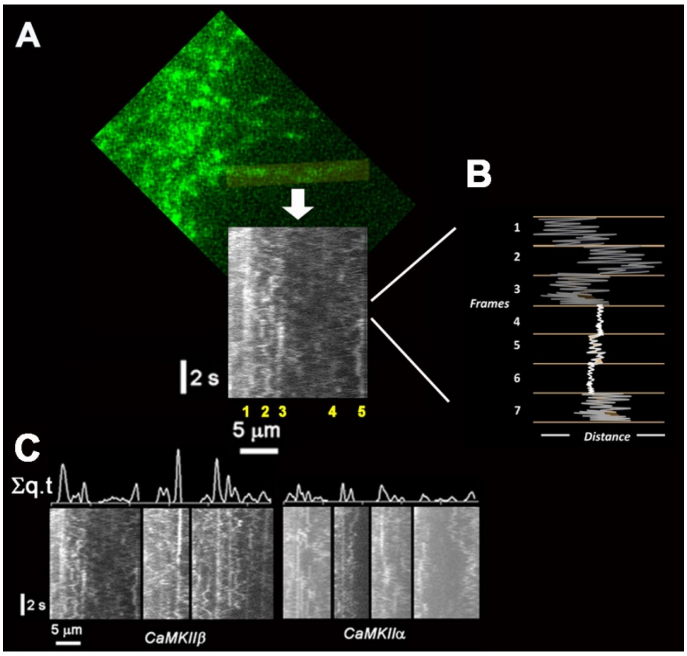

Fig.S3: CaMKII molecules in filopodia -A*. Creation of a kymograph. A single frame image of a filopodium (highlighted) was skeletonized, the skeletonized outline padded (5 pixels), then computationally straightened. The intensity along the length of the filopodium was measured with ImageJ. The stack of intensity profiles obtained from several frames formed a kymograph (length (abscissa), time (ordinate)). Bound or weakly bound molecules persist at one position along the filopodium generating a track in the stack. 5 tracks (yellow numerals) are seen in the kymograph. Tracks 3 and 5 have bright linear segments indicative of bound states and dim wavy segments indicative of unbound, diffusive states. Track 1 is predominantly bound. Track 4 is predominantly unbound.* ***B.*** *Schematic over seven frames of a segment from track 5 shows displacements within frames ($\delta t = 50$ms). The fluorescence image of a freely diffusing unbound molecule will be spread over an area greater than that occupied by its PSF when bound and immobilized. Colour (white = high, black = low) denotes intensity, q, of the computed centroid for each frame.* ***C.*** *Kymographs of native GFP-α and GFP-β homomeric complexes visualised within filopodia of HUVECs. The intensity profiles were summed over frames (white lines above the kymographs). The summed pixel intensities $(\Sigma qt)_x$ were fitted to Gaussian curves of form $(\Sigma qt)_x = a(exp(0.5((x-x_0)/b)^2))$, where "a" is the peak amplitude (counts/pixel), "x_0" the position of the peak along the filopodium, and "b" the distance x - x_0 (pixels) where the intensity is 1/e of the peak value. β and α have average (a / b) values of 23.5 / 4.4 and 10.7 / 3.8 respectively.*

B. Mutant Analyses

(1) The GFP-CaMKII fusion proteins form 2 distinct groups based on mobility – Co-localization of the proteins βT287A, βK43R and βA303R with stress fibres is comparable to β (β_{strong} group). In contrast, βT287D does not co-localize with the fibres (**Fig. S4**). ANOVA shows that α and β_{strong} form two distinct groups (**Table S1**). Mobility, as measured by V_{avg} has similar trend as measured from D_{lat} values. The intensity distributions are distinguished by their skewness. The skewness is due to long-lived tracks (Fig. S2). Control β populations have comparable mobility, independent of tRFP-actin (D_{lat} = 0.23±0.02 μm^2/s (Figure 6A) (+), 0.21 μm^2/s (-)) (**Fig. S5**).

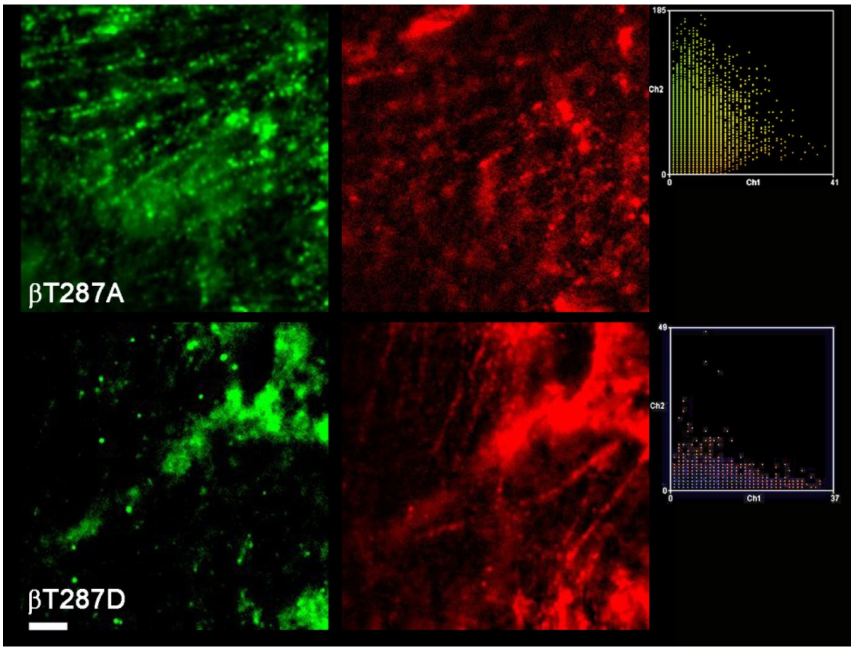

Fig. S4: Co-localization of βT286 point mutant proteins – *Left: Selected quadrants from the full image fields seen in Movies S2 (βT287A) and S3 (βT287D). The red and green images were merged for composite images in Figure 5B. Right: Scatter plots display pixel intensities with values along the diagonal indicating overlap, and non-diagonal values adjacent to the ordinate (ch 1: red) or abscissa (ch2: green) non-overlap. The plots give a visual representation of the overlap measured by the P_{pix} coefficients (Figure 5B).*

Data-Set	Df_B | Df_T	***F-Value***	***p-Value***
D_{lat}(Total)	10|101	21.24	2.2xe^{-16}
D_{lat}(α)	3|27	1.98	0.125
D_{lat}(β)	5|74	17.28	2.7xe^{-11}
D_{lat}(β_{strong})	3|78	1.31	0.276

Table S1: Mobility statistics. *Datasets drawn from different cultures for each GFP-CaMKII fusion were grouped (Total = all GFP-CaMKII proteins,* α *= GFP-CaMKII*α *proteins,* β *= GFP-CaMKII*β *proteins,* β_{strong} *=* β *–* β*T287D,* β*E'). F =* σ^2_B/σ^2_T*, where* σ^2_B *is the normalized variance between groups and* σ^2_T *is the normalized pooled variance.* Df_B *and* Df_T *are the degrees of freedom for* σ^2_B *and* σ^2_T *respectively. F = 1 in absence of any difference (null hypothesis). The p value is the probability that the null hypothesis is correct.*

(2) The mobility difference is not due to physical size - The magnitude of hindered diffusion has been measured for F-actin gels (7). Diffusion of 70 nm particles, twice the Stokes diameter, d of CaMKII holoenzymes, was not affected at 2 mg/ml F-actin. Mean mesh size, ε, scales with $c^{(-3/4)}$, where c is F-actin concentration, with the proportionality constant (=0.9) obtained from the diffusion of latex spheres (7). The relation between these parameters in cross-linked gels, derived by de Gennes as cited in (7), is

$$D_{lat} = D'_{Stokes}\left(exp\left(-\beta\left({}^{d}/_{\varepsilon}\right)^{2.5}\right)\right), \qquad \textbf{C}$$

where D'_{Stokes} is the Stokes diffusion coefficient in water times a correction factor for non-spherical shape. The dimensionless coefficient β is close to one. For stress fibres, the F-actin spacing determined by the α-actinin crosslinks (8) is 30 nm (9). Physical entrapment of the 25 nm CaMKII holoenzymes within fibres would decrease D'_{Stokes} 2.7 fold to 3.7 μm^2/ s. The predicted D_{lat} would have the decorating particles diffuse 10 μm^2 in 4 seconds in contrast to observation (**Movie S1**). Given an actin mesh size equivalent to an F-actin concentration of 1 mM (42 mg/ml), twice the value measured within the actin-rich lamellipodium (10); the diffusion coefficient of the particles would still be 80% of the value measured in pure water. The D_{lat} after retardation by the cortical actin gel is predicted to be at least 15 fold and 7.5 fold greater than the D_{lat} values for the strong and weak binding groups (**Figure 6A**) respectively. The low group D_{lat}s can only be due to binding interactions.

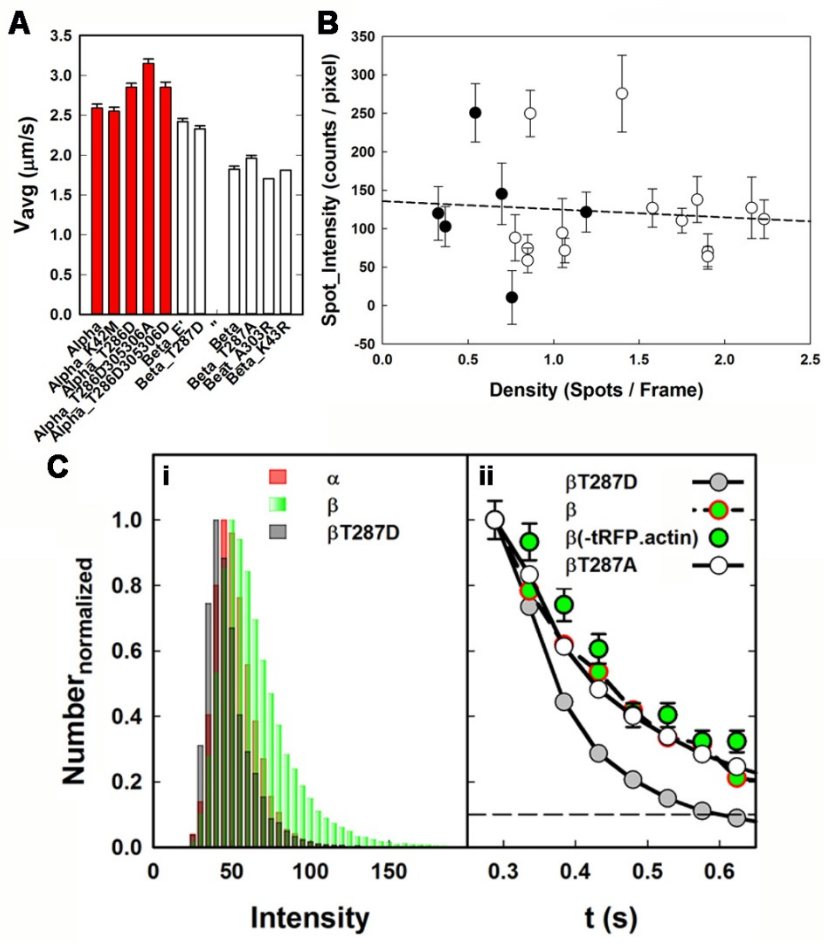

Fig. S5: Mobility differences are not due to aggregation / disassembly. A. *V_{avg} (mean $\pm$ se) values for the protein populations Red and open bars indicate different isoforms as in Figure 6.* ***B.*** *Spot intensity of αT287DT305306A variation with spot density (per 10 μm^2 evanescent field). Spot intensities were measured with the single fluorophore detection algorithm (SFDA) (2) that separates spots with PSF overlap, provided peak separation exceeds 1σ. Same culture imaged 24 hrs (closed symbols) and 38 hrs (open symbols) after transfection. Since αT287DT305306A does not bind F-actin, density in the evanescent field is an indicator of intracellular concentration. The concentrations estimated from the spot densities are in the nano-molar range but probably 10-fold higher since spots must be immobilized over 2 frames for detection. Linear regression (dashed line).* ***C. i.*** *Track population intensity histograms, with each normalized by the corresponding mode values.* ***ii.*** *βT287A and βT287D population track lifetime distributions. Control β populations with (green symbols, red edges) and without co-expressed tRFP-actin (green symbols, black edges. The distributions are normalized by the number of tracks in the first bin after the 5 frame (0.24 s) lifetime threshold. Horizontal dashed line indicates 10% of the initial population fraction.*

(3) Estimation of bound fraction and bound lifetimes – Track populations were divided into unbound and bound fractions based on a two-exponential best-fit (**Fig. S6**). The dwell-time in the evanescent field for the unbound population was set to the value obtained from the fit to the βT287D distribution. The dwell-time for the bound fraction was taken as the time for dissociation from F-actin. One bound species accounts for > 95% of the α and β populations. Bound fractions computed based on these majority species fall into two groups. Deviation from the two-exponential fits in the residual 0.05 population fraction reveals heterogeneity. A more refined statistical analysis (**Table S2**) based on a decade population decrease was not successful in establishing differences between βT287D and βE' or between βT287D and the three αT286D species.

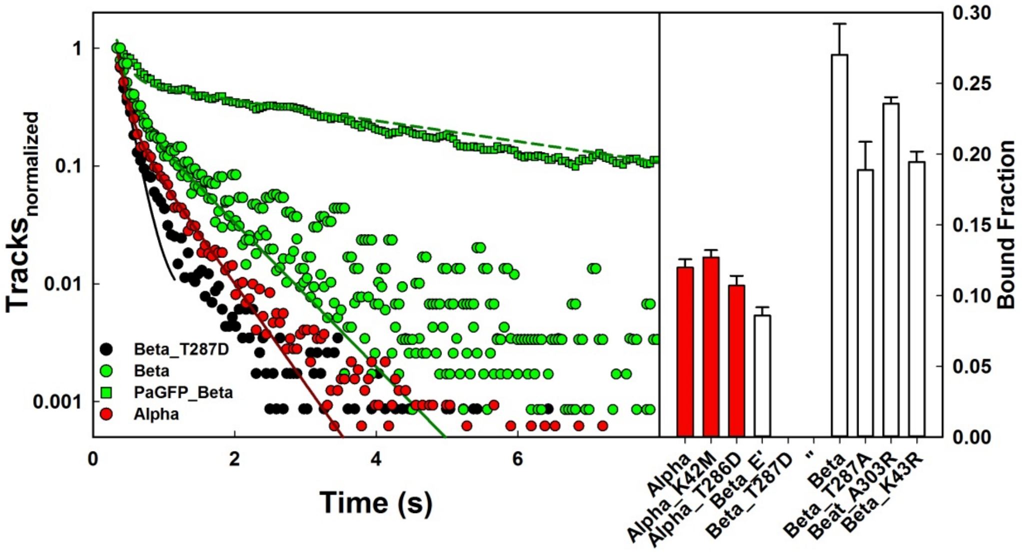

Fig. S6: Characterization of major binding modes. A. *The βT287D distribution was fit with a single exponential (black line). Two exponential best fits for the (β), (α) and PaGFP-β track distributions (coloured lines).* ***B.*** *Bound fractions ($\pm$ standard error) for the major bound CaMKII protein species estimated from two exponential fits with one exponent term fixed to the value determined from the βT287D fit (Equation 3) (k = 2.9 s^{-1} (α); 1.3 s^{-1} (β)). Cytoskeletal actin content was assumed to be the same.*

I. ANOVA

Data-Set	*Df|Residuals*	*F-Value*	*p-Value*
$Plot_{log}(\alpha)$	3|27	2.08	0.125
$Plot_{log}(\beta)$	5|29	18.46	$3.14xe^{-8}$
$Plot_{log}(\beta_{Strong})$	5|17	8.24	0.001
$Plot_{log}(\alpha+\beta_{weak})$	5|38	3.85	0.004

II. t-test (*wβT287D*)

CaMKII	*p-Value*	*CaMKII*	*p-Value.*
β	1.70E-07	α	0.0206
βA303R	1.20E-05	αK42M	0.0046
βK43R	1.80E-07	αT286D	0.2405
βT287A	0.00075	$\alpha T286D_{305306D}$	0.5471
βE'	1	$\alpha T286D_{305306A}$	1

Table S2: Track lifetime statistics. *The video-records used for the analysis of mobility were re-analysed for track lifetime differences. Df_B, Df_T, F-value, p-value and groups (α, β and β_{strong}) are as in Table S1. ($\alpha + \beta_{weak}$) = α + (βT287D, βE').* ***I.*** *ANOVA demonstrates that the GFP-CaMKII species partition into two groups based on bound life-times as well as mobility.* ***II.*** *Simultaneous pairwise t-tests conducted against βT287D, the reference for unbound species, dissect the basis for the group*

differences. The p-values indicate that the (α) and (β) isoforms as well as mutant species (βA303R), (βK43R), (βT287A) and (αK42M) bind; while absence of the spliced linker segment (βE') reduces binding to undetectable levels. T-D substitution at the primary phosphorylation site reduces binding in the β-isoform independent of the modification state of the secondary phosphorylation sites.

(4) Decay kinetics from PaGFP-fusion and GFP-fusion experiments - The difference in the track lifetime distributions measured from PALM experiments (PaGFP-fusions) and the GFP-fusion experiments (**Fig. S6**) is due to the different number of free (X) and bound (Y) fluorescent molecules imaged per unit area of the evanescent field in the two types of experiments. The number of fluorescent molecules N = (X+Y). At equilibrium; $\left[\frac{Y}{X}\right] = e^{-\Delta G/kT}$, where ΔG is is the free energy change upon binding. ka^{+} and ka^{-} are the rates, for the X -> Y, Y -> X transitions respectively.

In PALM experiments, the number of photo-activated, fluorescent molecules *N* will have the equilibrium $\left[\frac{Y}{X}\right]$ ratio. The fluorescence decay will be proportional to the loss dN/dt over time, *t*. It will have a fast component due to diffusion (D = diffusion coefficient) of unbound molecules from the evanescent field (depth *l*) and a slow component due to dissociation of bound molecules.

$$dN/dt = Xe^{-4Dt/l^2} + Ye^{-ka^{-}t},$$

In the absence of tracking errors, *N* tracks whose duration is determined by their lifetimes in the evanescent field will be generated from the *N* molecules. The comparison between the fluorescence intensity decay and the track lifetime distributions (Results A6) shows that tracking errors, like photo-bleaching, have a small (~10%) effect.

For GFP-fusion experiments, track lifetime distributions will, in addition, to GFP-CaMKII molecules present at the start of the video record contain contributions from molecules (N_F) entering the evanescent field during the video record. N_F is negligible in PALM experiments since the incoming molecules will predominantly be non-fluorescent PaGFP-CaMKII. The N_F molecules effectively available to bind F-actin will be:

$$\frac{ka^{+}}{\left(\left(4D/l^2\right) + ka^{+}\right)}$$

Therefore:

$$\frac{Y_N}{X_N} < \frac{Y}{X} \text{ given } \left(4D/l^2\right)$$

is comparable or greater than ka^{+}. Hence, the fast component of the decay rate will be over-estimated, and the bound fraction under-estimated.

$$dN/dt = (X + X_N)e^{-4Dt/l^2} + (Y + Y_N)e^{-ka^{-}t},$$

The difference would increase if additional binding states are generated from the initial binding state, consistent with the log-normal lifetime distributions, as the apparent ka^{+} will decrease. Tracking errors due to the greater density of fluorescent spots will also increase for the GFP-fusion experiments.

C. The Log-Normal Relation

Simulated log-normal plots (**Figure S5**) illustrate that the discrimination between the (β) and (α) isoforms depends on the time window accessed by the assay. The plots have the form "Log(F) = C_1(Log(1+C_2t))", where F = fraction of the initial bound population, t = time, C_1 is slope. The scaling factor C_2 = 10. The log-normal relation is a consequence of the fact that both binding partners are multi-subunit assemblies that permit multiple binding reactions. Conformational flexibility of both CaMKII and F-actin will facilitate multi-subunit engagement.

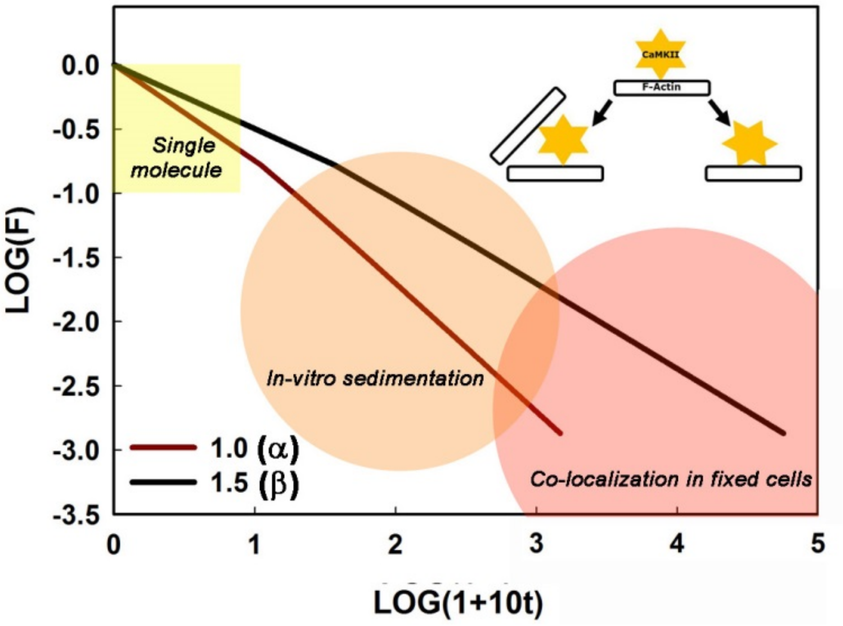

Figure S7: Simulated log-normal plots. *C_1 was taken as 1 for α and 1.5 for β to match the experimentally observed slope difference in the track lifetime relations between the two isoforms (Figure 7A). Single molecule assays monitor dissociation kinetics over a few seconds with sub-second resolution. The time for the first ten-fold population decrease (F (Log (F)) = 1(0) to 0.1(-1)) complete within 1 ((Log (1+10t)) ~ 1) second, differs by a factor of 4.7 fold for the two isoforms. In vitro bundling assays, limited by sedimentation times, capture interactions that are stable for one to many minutes. They monitor the decrease of the bound population over the second F decade (0.1 to 0.01). The β isoform takes 15.7 times as long as the α isoform over this range (1-100 seconds) for a ten-fold population decrease. Fixation times, typically minutes, limit co-localization assays in fixed cells. These assays visualize stably-bound complexes. For t>100 ((Log(1+10t)) ~ 3) seconds, < 0.1% of the bound α isoform population would remain, but probably would not be detected, as opposed to > 5% of the β population.* ***Inset:*** *Complex multi-subunit interactions between CaMKII (stars) and F-actin (rods) (schematized) can form utilizing combinations of reactions involving 2 F-actin or 2 CaMKII subunits.*

D. Supporting Movies

Movie SR1: Video-record for Figure 3A. The first section of the video shows tRFP-actin (red) and gfp-CaMKII-beta (green), played back in real time; the middle section shows the averaged intensity for both channels overlaid as a static image; the final section shows a section of the separate averaged images after local background subtraction). Co-localisation of β and F-actin is seen at actin stress fibres (long white arrows) as well as regions (membrane ruffles (short yellow arrows) expected to be rich in F-actin. CaMKII / stress fibre co-localization was quantified.

Movie S2: Video-record for Figure 5Bi. The first section of the video shows tRFP-actin (red) and βT287A (green), played back in real time; the middle section shows the averaged intensity data for both channels overlaid as a static image; then the single particle tracks are overlaid and finally the averaged tRFP-actin image is shown.

Movie S3: Video-record for Figure 5Bii. The first section of the video shows tRFP-actin (red) and βT287D (green), played back in real time; the middle section shows the averaged intensity data for both channels overlaid as a static image; then the single particle tracks are overlaid and finally the averaged tRFP-actin image is shown.

SUPPORTING REFERENCES

1. **Patterson, G. H. 2007. Photobleaching and photoactivation of fluorescent proteins for studies in cell biology. Micros Microanal 13.**
2. **Mashanov, G. I., and J. E. Molloy. 2007. Automatic detection of single fluorophores in live cells. Biophys. J. 92:2199-2211.**
3. **Schneider, C. A., W. S. Rasband, and K. W. Eliceiri. 2012. NIH Image to ImageJ: 25 years of image analysis. Nature methods 9:671-675.**
4. **Manders, E. M., Verbeek, F.J and Aten, J.A. 1993. Measurement of co-localization of objects in dual-colour confocal images. J. Microscopy 169:375-382.**
5. **Costes, S. V., D. Daelemans, E. H. Cho, Z. Dobbin, G. Pavlakis, and S. Lockett. 2004. Automatic and quantitative measurement of protein-protein colocalization in live cells. Biophys. J. 86:3993-4003.**
6. **Qian, H., M. P. Sheetz, and E. L. Elson. 1991. Single particle tracking. Analysis of diffusion and flow in two-dimensional systems. Biophys. J. 60:910-921.**
7. **Schmidt, C. F., Barmann, M., Isenberg, G., and Sackmann, E. 1989. Chain dynamics, mesh size and diffusive transport in networks of polymerized actin. A quasielastic light scattering and microfluorescence study. Macromolecules 22:3638-3649.**
8. **Pellegrin, S., and H. Mellor. 2007. Actin stress fibres. J. Cell Sci. 120:3491-3499.**
9. **Meyer, R. K., and U. Aebi. 1990. Bundling of actin filaments by alpha-actinin depends on its molecular length. J. Cell Biol. 110:2013-2024.**
10. **Koestler, S. A., K. Rottner, F. Lai, J. Block, M. Vinzenz, and J. V. Small. 2009. F- and G-actin concentrations in lamellipodia of moving cells. PloS one 4:e4810.**
