# Supplementary figures and images for: Multiple CaMKII Binding Modes to the Actin Cytoskeleton Revealed by Single-Molecule Imaging
Source: Biophys J. 2016 Jul 26;111(2):395–408. doi: 10.1016/j.bpj.2016.06.007 (PMC4968397; doi:10.1016/j.bpj.2016.06.007)

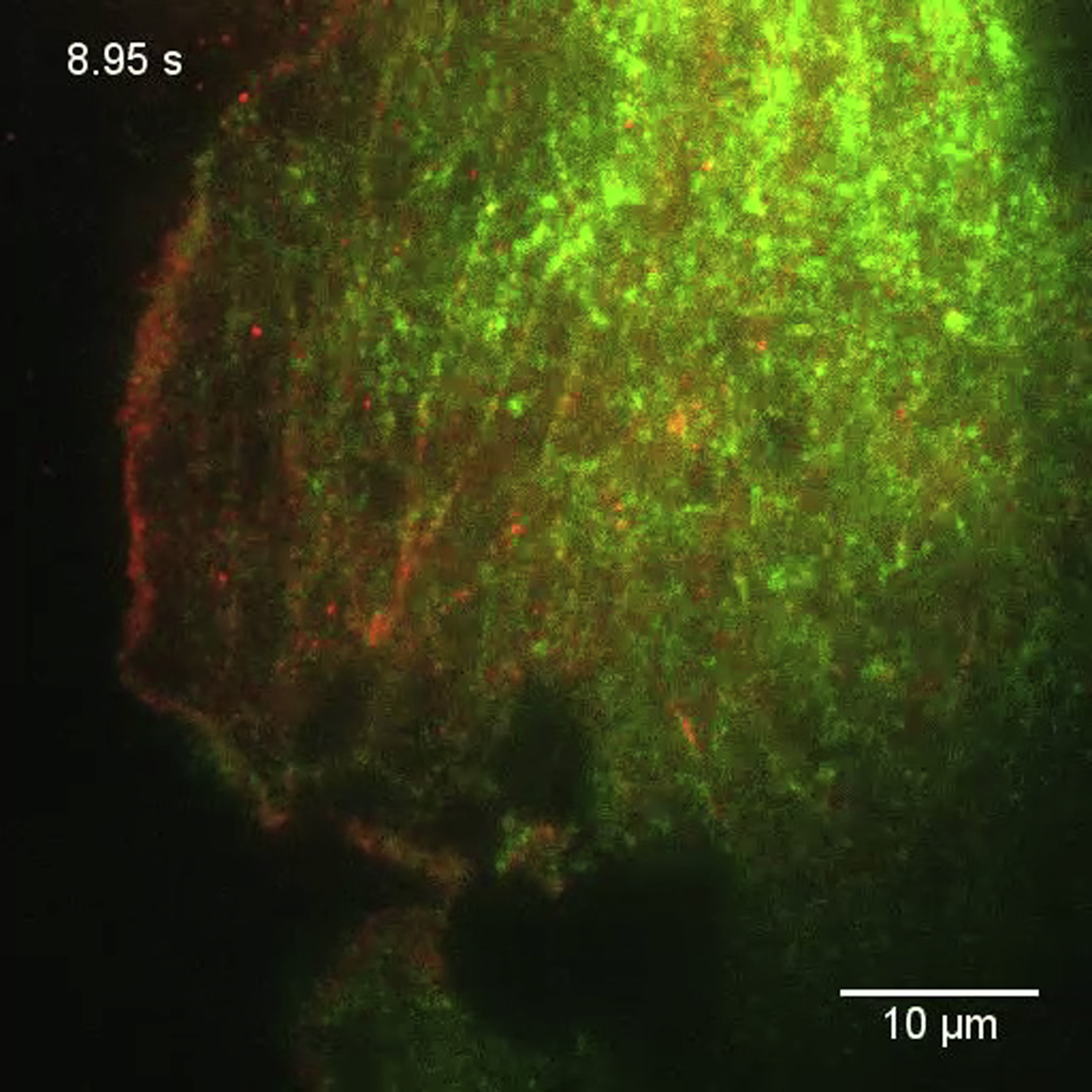

Supplement: Movie S1. Video Recording for Fig. 3 A — The first section of the video shows tRFP-actin (red) and gfp-CaMKII-β (green), played back in real time; the middle section shows the averaged intensity for both channels overlaid as a static image; and the final section shows a section of the separate averaged images after local background subtraction. Colocalization of β and F-actin is seen at actin stress fibers (long white arrows) as well as in regions that are expected to be rich in F-actin (membrane ruffles, short yellow arrows). CaMKII/stress-fiber colocalization was quantified. [file mmc2.jpg]

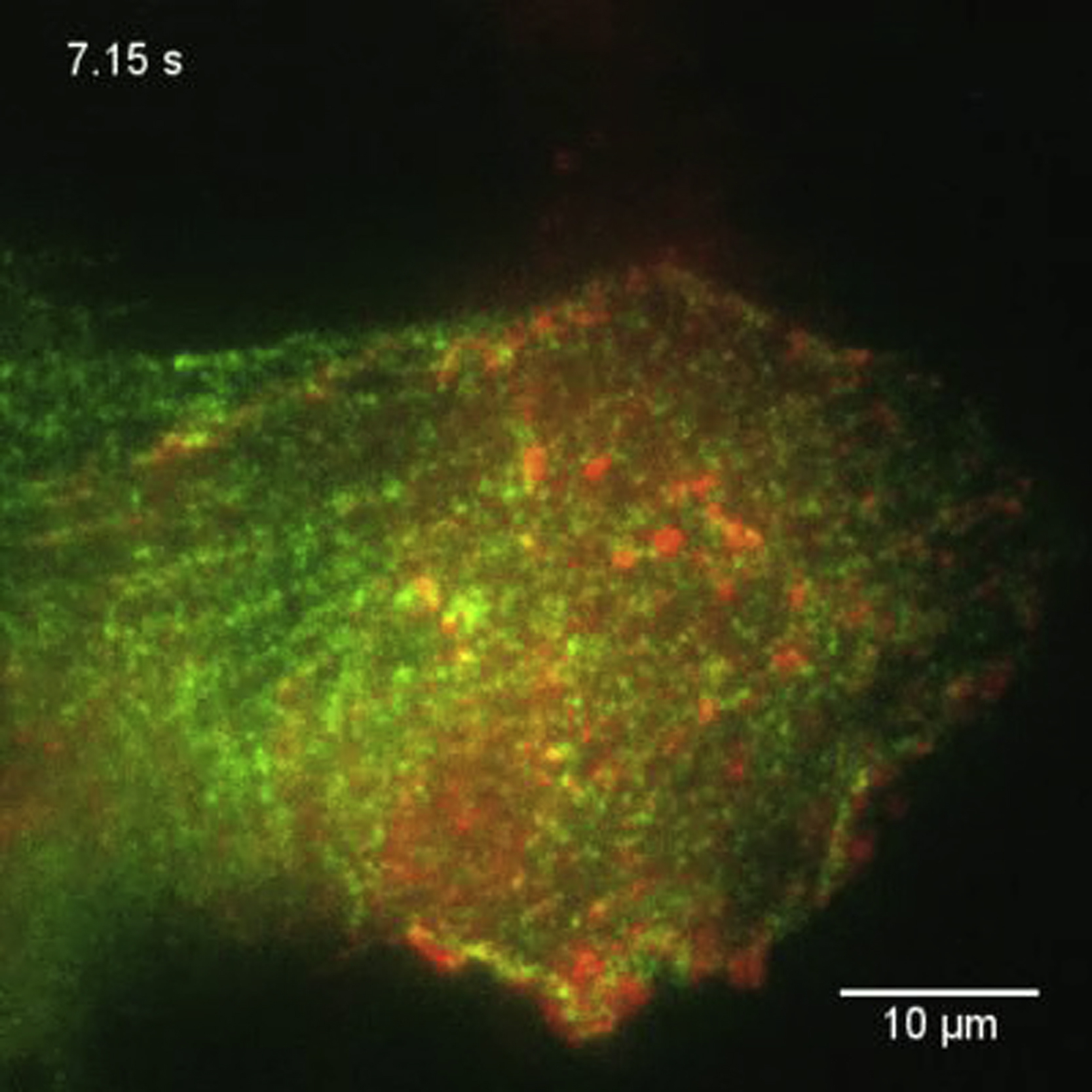

Supplement: Movie S2. Video Recording for Fig. 5 B(i) — The first section of the video shows tRFP-actin (red) and βT287A (green), played back in real time, and the middle section shows the averaged intensity data for both channels overlaid as a static image. The single-particle tracks are overlaid and finally the averaged tRFP-actin image is shown. [file mmc3.jpg]

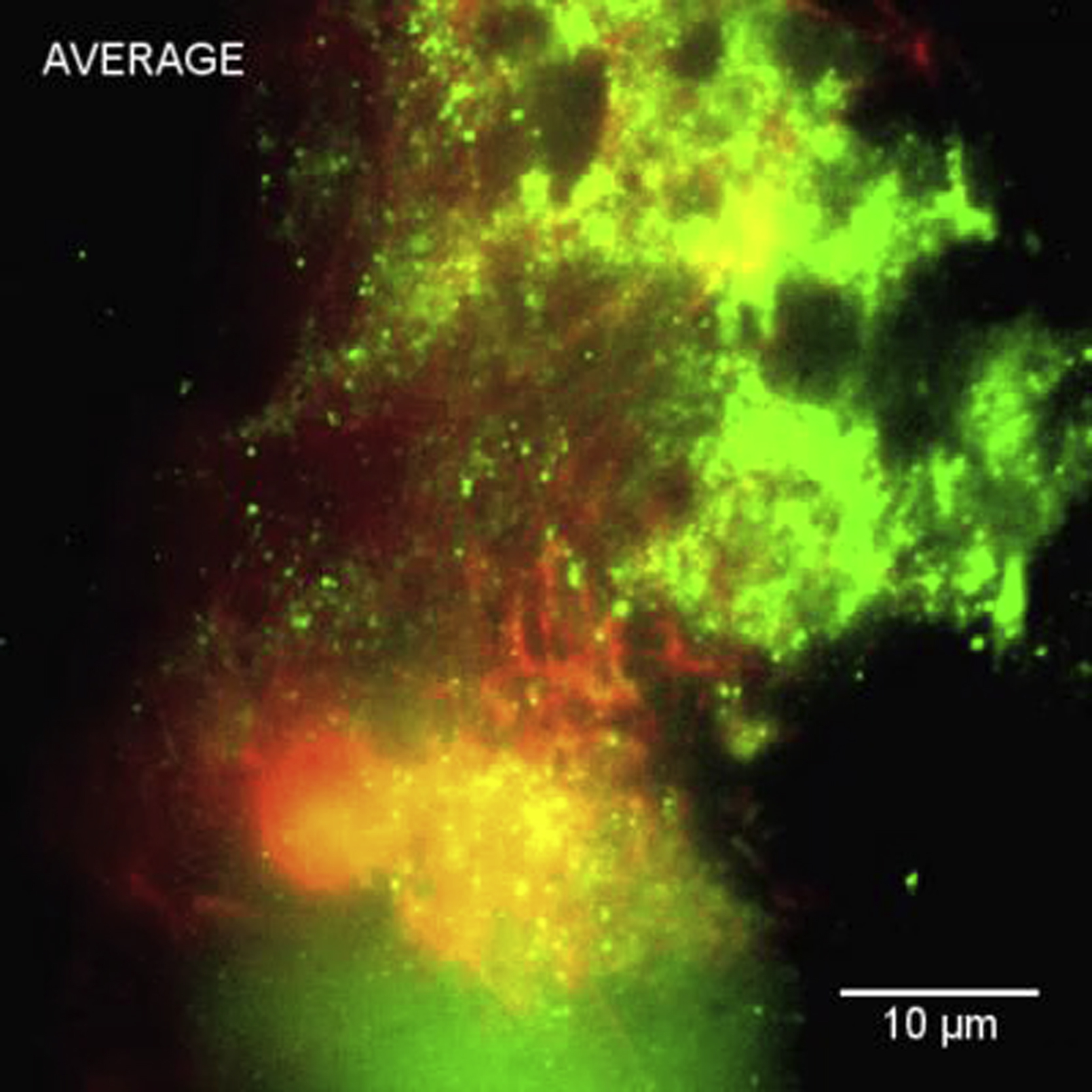

Supplement: Movie S3.Video Recording for Fig. 5 B(ii) — The first section of the video shows tRFP-actin (red) and βT287D (green), played back in real time, and the middle section shows the averaged intensity data for both channels overlaid as a static image. The single-particle tracks are overlaid and finally the averaged tRFP-actin image is shown. [file mmc4.jpg]
