# Supplementary material for: Multiple CaMKII Binding Modes to the Actin Cytoskeleton Revealed by Single-Molecule Imaging
Source: Biophys J. 2016 Jul 26;111(2):395–408. doi: 10.1016/j.bpj.2016.06.007 (PMC4968397; doi:10.1016/j.bpj.2016.06.007)
Supplement: Document S2. Article plus Supporting Material [file mmc5.pdf]

Biophysical Journal
Article

Biophysical Society

Multiple CaMKII Binding Modes to the Actin Cytoskeleton Revealed by Single-Molecule Imaging

Shahid Khan,[1,*] Ianina Conte,[2] Tom Carter,[3] K. Ulrich Bayer,[4] and Justin E. Molloy[5]
[1]Molecular Biology Consortium, Lawrence Berkeley National Laboratory, Berkeley, California; [2]Cardiovascular and Cell Science Research Institute and [3]Cell Biology and Genetics, St. George's University of London, London, UK; [4]Department of Pharmacology, University of Colorado Denver, Aurora, Colorado; and [5]The Francis Crick Institute, Mill Hill Laboratory, London, UK

ABSTRACT Localization of the Ca^{2+}/calmodulin-dependent protein kinase II (CaMKII) to dendritic spine synapses is determined in part by the actin cytoskeleton. We determined binding of GFP-tagged CaMKII to tag-RFP-labeled actin cytoskeleton within live cells using total internal reflection fluorescence microscopy and single-molecule tracking. Stepwise photobleaching showed that CaMKII formed oligomeric complexes. Photoactivation experiments demonstrated that diffusion out of the evanescent field determined the track lifetimes. Latrunculin treatment triggered a coupled loss of actin stress fibers and the colocalized, long-lived CaMKII tracks. The CaMKIIα (α) isoform, which was previously thought to lack F-actin interactions, also showed binding, but this was threefold weaker than that observed for CaMKIIβ (β). The βE′ splice variant bound more weakly than α, showing that binding by β depends critically on the interdomain linker. The mutations βT287D and αT286D, which mimic autophosphorylation states, also abolished F-actin binding. Autophosphorylation triggers autonomous CaMKII activity, but does not impair GluN2B binding, another important synaptic protein interaction of CaMKII. The CaMKII inhibitor tatCN21 or CaMKII mutations that inhibit GluN2B association by blocking binding of ATP (βK43R and αK42M) or Ca^{2+}/calmodulin (βA303R) had no effect on the interaction with F-actin. These results provide the first rationale for the reduced synaptic spine localization of the αT286D mutant, indicating that transient F-actin binding contributes to the synaptic localization of the CaMKIIα isoform. The track lifetime distributions had a stretched exponential form consistent with a heterogeneously diffusing population. This heterogeneity suggests that CaMKII adopts different F-actin binding modes, which is most easily rationalized by multiple subunit contacts between the CaMKII dodecamer and the F-actin cytoskeleton that stabilize the initial weak (micromolar) monovalent interaction.

INTRODUCTION

The calcium calmodulin-dependent kinase (CaMKII) is a multifunctional kinase that has a prominent role in long-term potentiation (LTP) (1–3). The four major isoforms of vertebrate CaMKII have ~40 splice variants and are expressed in diverse tissues (3). Two isoforms, CaMKIIα ($<\alpha>$) and CaMKIIβ ($<\beta>$), are dominant in the brain and their relative expression levels vary among different regions of the brain as well as during development (4). Their relative levels also vary within individual neurons between the cell body and dendritic/axonal processes (2). CaMKII has a prominent structural role in hippocampal dendritic spines, the postsynaptic computational units for LTP. CaMKII concentrations in spines are high (5), consistent with its structural role. The $<\beta>$ isoform targets $\alpha\beta$ hetero-oligomers to dendritic spines by binding to the spine actin cytoskeleton (6). Synaptic stimulation triggers CaMKII sequestration to dendritic spines and the postsynaptic density (PSD) within a few seconds of stimulation (7–10). This rapid sequestration is coupled to actin polymerization and expansion of the stimulated spine (11). Expansion is due to the direct effects of CaMKII on the actin cytoskeleton (12,13) as well as to indirect effects mediated by the activation of other kinases (14). The increase in spine size persists after termination of the stimulus-induced calcium transient. CaMKII levels in stimulated spines are also increased due to association with the PSD, in particular, the NMDA receptor GluN2B subunit (15) and the enlarged actin cytoskeleton (16). In the longer term, CaMKII promotes axonal branching and outgrowth (17).

The neuronal isoforms have highly homologous kinase and association domains, but the linker that connects these two domains is variable in sequence and length (1). The individual subunits assemble into homo- or hetero-oligomers of variable isoform compositions, and the atomic structure of the dodecameric enzyme has been previously described

Submitted December 16, 2015, and accepted for publication June 9, 2016.
*Correspondence: khan@mbc-als.org
Editor: Cecile Sykes.
http://dx.doi.org/10.1016/j.bpj.2016.06.007
© 2016 Biophysical Society.
This is an open access article under the CC BY license (http://creativecommons.org/licenses/by/4.0/).
CrossMark

(18). The $<\alpha>$ and $<\beta>$ isoforms form 12 subunit homo-oligomers of similar size, with one study reporting a slightly smaller $<\beta>$ oligomer (19). Calmodulin binding to the regulatory segment relieves inhibition, and transphosphorylation activates the enzyme at $<\alpha>$T286 (T287 in the other isoforms), which confers autonomous activity to the enzyme.

Binding of $<\beta>$, but not $<\alpha>$, to the actin cytoskeleton has been shown by various approaches, including colocalization, fluorescence photobleaching, and pharmacological manipulations in neuronal and non-neuronal cell cultures (6,20–23). In vitro sedimentation assays and electron microscopy have demonstrated the $<\beta>$-dependent formation of F-actin bundles (12,22–24). Activation of $<\beta>$ by both autophosphorylation and the phosphomimetic T287D mutation (22) abolishes actin bundling activity. Furthermore, an alternative splice variant, $<\beta E'>$, which has a short linker similar to that of $<\alpha>$, does not bind or bundle F-actin in pull-down assays. The differences observed with the mutants in pull-down assays are consistent with colocalization in neuronal cell cultures. Pyrene fluorescence measurements (12) have shown that both $<\alpha>$ and $<\beta>$ isoforms bind globular (G-) actin, and $<\beta>$ binds with 2.4 μM affinity and a stoichiometry of 12 actin monomers per oligomer (24). However, quantitative estimates of the affinity of $<\beta>$ or $<\alpha>$ for F-actin, or of modulation via activation through stimulation or mutation, are not available.

Here, we characterized the association of CaMKII with labeled F-actin in live human umbilical vein endothelial cells (HUVECs) (25) by using total internal reflection fluorescence microscopy (TIRFM) to image and track single molecules (26,27). We previously exploited this approach to study motor proteins, ion channels, and G-protein-coupled receptors (27–30). Here, we extended the method to measure the association of enhanced green fluorescent protein (eGFP)-tagged CaMKII native and mutant proteins with red fluorescent protein (RFP)-tagged actin to mark the cytoskeletal structures. Single-molecule tracking experiments have shown that actin depolymerization increases CaMKII mobility in dendritic spines, and revealed different, heterogeneous mobility distributions for stimulated versus unstimulated states (16). We used HUVECs as a model system because they are ideal for TIRF imaging, have a defined cytoskeletal architecture, and are amenable to transient transfection methods. Our measurements show that both neuronal CaMKII isoforms bind cytoskeletal actin, but with affinities that differ by threefold over the first decade range of a log-normal binding curve. Our results explain why association of $<\alpha>$ may have been overlooked in earlier studies, and have implications for CaMKII transport and cytoskeletal remodeling within neurons.

MATERIALS AND METHODS

All biochemicals were sourced from Sigma-Aldrich (Poole Dorset, UK) unless noted otherwise.

TIRFM

We used a custom-built TIRF microscope workstation based on an inverted microscope (Nikon Eclipse, TE 2000U; Nikon, Kingston-upon-Thames, UK) (Fig. 1). Complete details are provided in Supporting Materials and Methods in the Supporting Material.

In vitro assays

For use as a single fluorophore calibration specimen, we immobilized GFP molecules on the surface of a microscope flow cell with a GFP antibody by first filling the flow cell with a phosphate-buffered saline (PBS) solution (pH 7.4) containing 5 μg/mL (3 nM) polyclonal anti-GFP antibody (Abcam, Cambridge, UK) as described previously (27). This solution was left to incubate in the flow cell for 5 min and then washed with PBS supplemented with 0.5 mg/mL bovine serum albumin to block regions of bare coverglass. The solution was then replaced with PBS containing 10 ng/mL (0.37 nM) GFP (Clontech, Palo Alto, CA) for 5 min, and unbound protein was washed out of the flow cell by several washes with assay buffer (AB^- (20 mM imidazole (pH 7.4), 50 mM KCl, 2 mM EGTA, 4 mM $MgCl_2$)) before it was viewed by TIRFM. The molecules were imaged in degassed and argon-purged AB^- supplemented with an oxygen-scavenger system consisting of 3 mg/mL glucose, 0.5 mg/mL catalase, 0.2 mg/mL glucose oxidase, and 20 mM dithiothreitol. Using the antibody-immobilized GFP molecules as a control sample, we measured the single fluorophore intensity as a function of excitation power. The average value measured over several hundred fluorophores was linear with the laser power. The mean single fluorophore intensity could therefore be used as an independent internal check of excitation power in subsequent experiments.

Cell culture

CaMKII fusion proteins tagged with monomeric eGFP (GFP) or photoactivatable eGFP (PaGFP) carrying the A206K mutation have been described previously (22,31–34). The GFP tag does not interfere with kinase activity

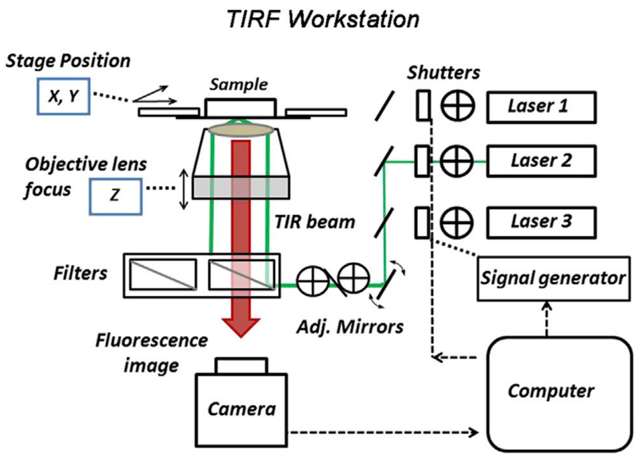

FIGURE 1 TIRF workstation. The choice of the laser excitation wavelength (laser 1 = 561 nm; laser 2 = 488 nm) was computer controlled; excitation (*green line*) and fluorescence emission (*red arrow*) light paths are shown. The TIRF incident angle was adjusted by an external mirror. The microscope stage and objective lens employed piezo-positioners to control specimen position and image focus. Images were acquired with an EMCCD camera. A waveform generator set the duration, delay, and frequency of photoactivation pulses (laser 3 = 405 nm), also in TIRF mode. Separate, exchangeable filter cassettes were used for GFP and tRFP fluorescence.

or holoenzyme assembly (24), and immunoelectron microscopy has shown that native CaMKII sequesters to the PSD of dendritic spines (35) with kinetics similar to those reported by the tagged proteins (22,36). We studied the following tagged actin fusion proteins: mCherry-actin (37), tagRFP-actin (38), and mTurquoise2-actin (39). We chose tagRFP-actin (tRFP-actin) for its brightness, photostability, and expression level (40). The plasmids encoding GFP-CaMKII and tRFP-actin constructs were mixed and cotransfected into primary HUVECs or Cos7 cells at 70–80% confluence, primarily by nucleofection (Nucleofector Model 2b; Lonza, Blackley, UK). Alternatively, Lipofectamine-2000 (Life Technologies, Paisley, UK) transfection was used as previously described (41). With either method, the transfection efficiency was typically >50%. The cells were plated on poly-lysine-coated dishes (Lab-Tek chambered borosilicate, #1 coverglass; Nunc, Rochester, NY) in Dulbecco's modified Eagle's medium with added 10% fetal bovine serum and streptomycin (50 μg/mL). Cell culture dishes were removed from the CO_2 incubator (Galaxy R; Scientific Laboratory Supplies, East Riding of Yorkshire, UK) 24–36 h after transfection. These incubation times were optimal for visualizing single GFP-CaMKII molecules. TIRF imaging was conducted at 25°C within an hour after the samples were removed from the incubator.

The HUVECs we chose as a model system for most of our TIRF imaging experiments attach firmly to the culture dish substrate and have long ventral stress fibers (42) that form oriented arrays. Other cytoskeletal substructures (i.e., arcs (43) and filopodia) are also present. Although HUVECs express a variant CaMKIIδ6 isoform (44), they do not natively express the <α> or <β> isoforms found in neurons. Expression was monitored by epifluorescence, and cell morphology was determined by phase contrast. In addition to morphology, we checked the integrity of the physiological state by noting an absence of CaMKII aggregation caused by high pH or calcium (36).

Single-molecule image analysis

A typical experiment involved a set of cotransfections of the plasmid encoding tRFP-actin with a plasmid encoding a GFP-CaMKII fusion (two dishes per CaMKII construct; up to four constructs per experiment). Control dishes cotransfected with plasmids encoding tRFP-actin and GFPCaMKIIβ were included in each experiment to assess the viability of the primary culture. First, tRFP-actin fluorescence was used to identify transfected cells, and then GFP-fluorescence was recorded. Many thousands of single-particle tracks were obtained for each construct using >12 cells from four different culture dishes and two separate experiments. Details regarding the single-particle tracking algorithm and the analytical measures used are provided in Supporting Materials and Methods.

Multiple analysis of variance (ANOVA) and simultaneous pairwise t-tests were conducted in R (https://www.r-project.org/) as detailed in (45). The variance was the sum of the variation within and between groups normalized by their degrees of freedom. The probability (p-value) that differences between populations were significant was then computed from the F-value (F). Significant differences reported by ANOVA were then tested by means of simultaneous, pairwise t-tests with default Holm correction for multiple testing.

RESULTS

Our experimental study consisted of two parts, First, we used dual-color TIRFM to visualize and track (27) individual GFP-tagged <α> and <β> isoforms in HUVECS, and derived their properties from population statistics and spatial colocalization with F-actin cytoskeletal structures. We then studied different mutants and pharmacological agents to understand the structural basis of CaMKII association with F-actin.

Assay development

Visualization of GFP molecules in control specimens and live cells

We visualized antibody-immobilized GFPs at low surface density (<1 μm^{-2}) using TIRFM to establish the emission intensity of individual GFP molecules under our standard imaging conditions. Individual GFPs were readily identified as discrete fluorescent spots that had a diffraction-limited point spread function (PSF) with a characteristic spot intensity (Fig. 2 *A*). The spots had a mean duration of 2.0 ± 0.4 s and exhibited single-step photobleaching with a unitary intensity of 27.4 ± 2.2 counts/pixel. Brighter spots with twofold greater intensity exhibited two-step photobleaching (Fig. 2 *B*). Next, we obtained TIRFM video recordings of cultured HUVECs and Cos7 cells that were expressing

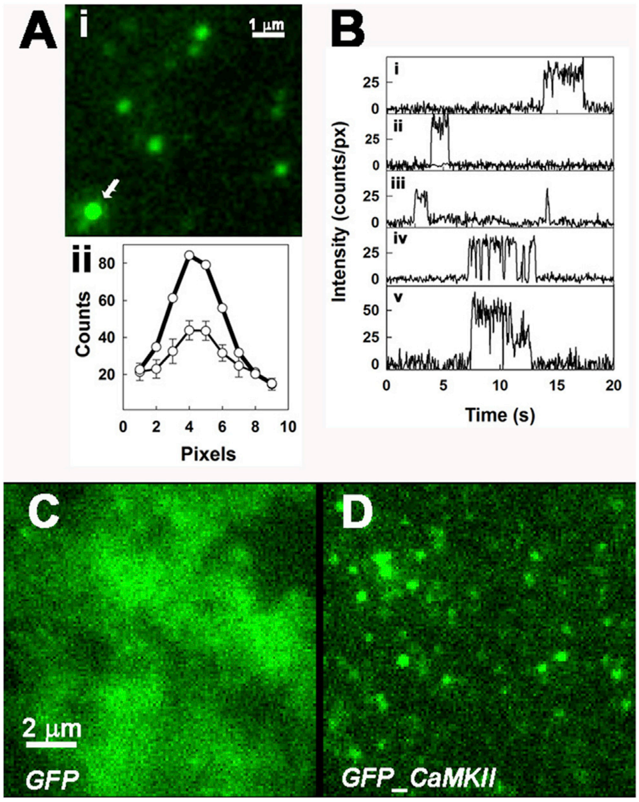

FIGURE 2 TIRFM visualization of GFP in vitro and in living cells versus GFP-CaMKII. (*A*) *i*: Antibody-immobilized GFP molecules (10-frame averaged image)). *ii*: Line intensity profiles of the four spots in field center, top and right (± standard error (SE), *thin line*), and of the brighter spot (*arrow*) show the diffraction-limited size. (*B*) Intensity-versus-time records of spots shown in *A*(*i*), illustrating single-step photobleaching (*i–iii*), blinking behavior (*iv*), and double-step photobleaching of the brighter spot (*v*).The single-step modal value was 27.5 ± 2.5 counts/pixel (doubling and tripling occurs when fluorophore PSFs overlap). (*C*) Single video frame (50 ms exposure) of a HUVEC expressing GFP alone, showing that motion blurring prevents single fluorophore observation. (*D*) Single video frame of a HUVEC expressing GFP-CaMKIIβ, showing that discrete fluorescent spots are now visible.

GFP. In contrast to the video recordings of antibody-immobilized GFP molecules, the GFP fluorophores within cells could not be resolved (Fig. 2 *C*). This was because rapid diffusive motion within the cytosol caused image blurring during the frame acquisition period, as explained below.

Visualization of homomeric GFP-CaMKIIβ complexes in the cellular cortex

In marked contrast to cell cultures expressing GFP molecules alone (see above), single fluorescent particles were visualized by TIRFM in cell cortices when GFP-tagged $<\beta>$ (henceforth termed β) was expressed (Fig. 2 *D*). This discrepancy can be explained by attenuation of the spot intensity by motion blurring during the 50 ms frame acquisition period (δt). The attenuation factor of the computed centroid is given by the ratio of the area covered by the diffusing particle during a single video frame $= \pi(\delta x)^2$ (where $\delta x = (4D.\delta t)^{1/2}$, and D is the diffusion coefficient) and the area that captures 90% of the object's PSF (here a 3 × 3 pixel region on the camera) $= 0.9\ \mu m^2$.

The expected lateral diffusion coefficients, D_{Stokes}, for the relevant species were computed from the diffusion equation:

$$D_{Stokes} = \frac{k_b T}{6\pi\eta a_s}, \tag{1}$$

where Stokes radius $a_s = (3M/4\pi A\sigma)^{1/3}$, M is the molecular mass (kDa), A is Avogadro's number, σ is the protein density (1300 kg/m^3) (46), and η is the cortical viscosity (0.0032 Pa.s) (47,48). This gives an estimated D_{Stokes} for GFP (M = 27 kDa; a_s = ~2 nm) of ~30 $\mu m^2 s^{-1}$ and diffusive motion blurring during a 50 ms video frame of ~20 μm^2. Therefore, the expected reduction in fluorescence intensity (per pixel) is 20/0.9, ~23-fold. This explains why freely diffusing GFP molecules were not resolved at the video imaging rates. Since D_{Stokes} varies inversely as the cube root of the molecular mass, we were also unable to resolve the GFP-$<a>$ (henceforth α) mutant, which is monomeric due to deletion of the association domain ($\alpha\Delta316$) (M = 62 kDa) and tRFP-G-actin (M = 70 kDa), as both exhibit a >15-fold estimated attenuation of spot intensity due to motion blurring. We were able to satisfactorily visualize β molecules because they form dodecameric complexes (M = 87 × 12 = 1044 kDa) (18,49). Thus, the intensity attenuation by motion blurring (~7-fold) is more than compensated for by the 12-fold increase in intensity due to the increased number of GFPs.

Decoration of actin stress fibers with single GFP-CaMKIIβ holoenzymes

We used two-color TIRFM to image β (excited at 488 nm) and tRFP-actin (excited at 561 nm) to characterize β complexes interacting with F-actin cortical structures. TIRFM of HUVECs transfected with t-RFP actin revealed long linear fibers in the actin cortex. The morphology was consistent with ventral stress fibers (42), and these structures will henceforth be referred to as such. Approximately 100 video frames were averaged to enhance the relatively static fibers above the background of rapidly diffusing G-actin monomers. The averaged tRFP-actin image was then overlaid onto TIRFM video recordings of β molecules to reveal their movement within the cytosol and their association/dissociation with the tRFP-tagged F-actin structures (Movie S1).

Individual GFP-fluorescent spots were identified and tracked in the video sequences to yield spatiotemporal trajectories (300–3000 per record) of individual objects. Tracks were generated by linking centroids for successive frames. Apparent diffusion coefficients (D_{lat}) were computed from the centroid frame-to-frame displacements (Δx):

$$D_{lat} = (\Delta x)^2 / (4t^c). \tag{2}$$

For free diffusion, $c = 1$. For confined diffusion, $c = >1$ and the denominator preexponent $= <4$. Individual tracks terminated when the object intensity dropped below the detection threshold due to diffusion from the excitation region (evanescent field), photobleaching, or tracking errors (considered below). Superposition of the image showing all of the particle tracks obtained over one video recording (lasting 25 s) onto the averaged tRFP-actin image provided a measure of colocalization (Pearson's correlation coefficient, P_{pix}), as described in Supporting Materials and Methods. It was clear that β associated with the cortical actin fibers (Fig. 3 *A*).

Dynamics of the interaction between CaMKIIβ and the actin cytoskeleton

Automated single-particle tracking (27) was used to identify and track individual β complexes. The object tracks were characterized with the measures defined in Materials and Methods. Short-lived particle trajectories ($t < 0.58$ s; Fig. 3 *B*, *yellow symbols*) closely approximated Brownian motion. In contrast, the plot for longer-lived trajectories ($t > 2.5$ s; Fig. 3 *B*, *blue symbols*) was nonlinear, with little increase in the mean-square deviation (MSD) beyond $\Delta t >$ 1.2 s. Further analysis showed that the binned subpopulation of short-lived tracks had a unimodal intensity distribution with a lower mean relative to the parent population, whereas the subpopulation of longer-lived tracks had higher intensity relative to the parent population and the intensity distribution was greatly skewed toward higher values (Fig. 3 *C*). The different subpopulation characteristics are consistent with the notion that tracks from weakly bound, more mobile molecules have a short duration and dominate the <0.5 s subpopulation. In contrast, more strongly bound molecules dominate the >2.5 s subpopulation, with lower average D_{lat}. The modal intensities for both subpopulations are lower than expected for the multimeric (10–12 subunits) tagged β holoenzymes. Thus, although at first it may seem

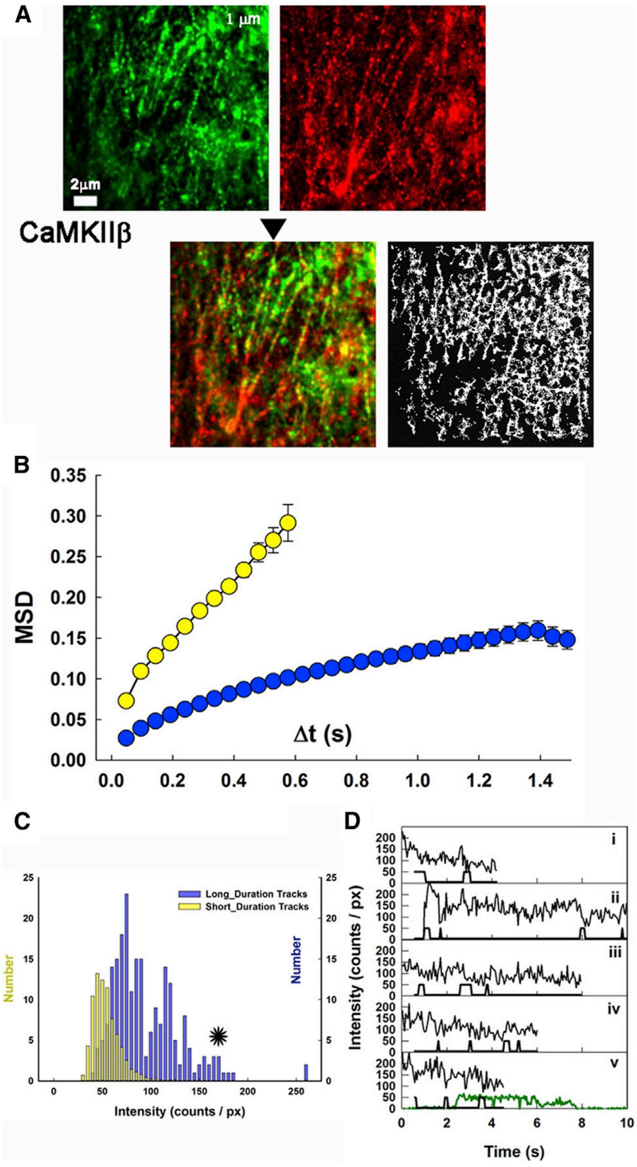

FIGURE 3 CaMKIIβ decoration of the actin cytoskeleton visualized by two-color TIRFM. (*A*) Averaged images of β (*left panel*, *green*, 400 frames) and tRFP-actin (*right panel*, *red*, 100 frames). Mean tRFP intensity = 119 ± 9 counts/pixel. The bottom panels (*arrow*) show the two frames superimposed ($P_{pix} = 0.27$, $P_{rand} = 0.09 \pm 0.07$) (*left*) and the single-particle tracks (*right*) accumulated over 10 s of video (Movie S1). (*B*) MSD-versus-time interval (Δt) for the total population of tracks (*white circles*) and short-lived (*yellow circles*) and long-lived (*blue circles*) track subpopulations (± standard deviation (σ)). The initial gradient of the short-lived track data gives $D_{lat} = 0.28\ \mu m^2/s$, whereas that of the long-lived tracks gives 0.04 $\mu m^2/s$. Total number of tracks, $n = 12{,}723$. (*C*) Intensity histograms for the short-lived track subpopulation (*yellow bars*) and long-lived track subpopulation (*blue bars*). The asterisk (*black*) marks the region of the histogram that was used to analyze photobleaching. (*D*) Sample intensity-versus-time plots for some of the objects from the asterisk-marked region. Stepwise intensity changes as detected by Student's *t*-test (Fig. S1) are marked immediately below each trace to indicate sudden intensity transitions. The starting intensity for each spot was >170 counts/pixel, which is ~8-fold greater than the unitary GFP intensity. The green line (in the *lowest panel*), is the two-step immobilized GFP photobleaching, redrawn from Fig. 2 *B*(*v*), shown for reference.

that the correlation between intensity and mobility differences is simply due to a difference in aggregate size, it is better explained by an intensity attenuation due to motion blurring (see above). To ascertain whether this was the case, we examined single spots and tracks.

To test for multisubunit states, we measured stepwise changes in fluorescence intensity. Spots immobilized on actin stress fibers had the highest intensities, but they exhibited PSF-limited spatial profiles similar to those obtained for single GFP fluorophores (Fig. 2 *A*). A small subset of such spots was analyzed (Fig. 3 *D*). The average initial intensity was ~8-fold greater (171.6 ± 11.5 counts/pixel) than that measured for individual GFP molecules in vitro (~27 counts/pixel). We used a running Student's *t*-test to detect significant jumps in local mean intensity over adjacent sections of data (Fig. S1 in the Supporting Material). The mean intensity drop for each stepwise change in intensity was 22.1 ± 2.0 counts/pixel and the mean step duration was 2.6 ± 0.4 s, which are similar to the values obtained for single GFP molecules immobilized in vitro (27.4 ± 2.2 counts/pixel and 2.0 ± 0.4 s). Many of these spots showed a severalfold greater final intensity drop (e.g., 90–0 counts/pixel (spot *v*)) relative to the 27.4 ± 2.2 counts/pixel drops obtained for single GFP photobleaching. Simultaneous photobleaching of multiple (three for spot *v*) GFP fluorophores is not likely. Instead, the final intensity drops presumably report the dissociation of β holoenzymes from the fibers and diffusion out of the evanescent field before all their fluorophores have bleached.

Sample tracks and their MSD versus Δt plots were analyzed next (Fig. S2). In addition to high intensities, the long-lived tracks had highly nonlinear MSD versus Δt plots, and the MSD and Δt correlation was abolished for intervals greater than a few frames, consistent with immobilization as validated by an examination of the single tracks. Centroid intensity inversely correlated with mobility in the short-lived tracks of diffusing spots, with values consistent with the motion-induced sevenfold attenuation relative to the intensity of immobilized holoenzymes. The slopes (MSD versus Δt) of these short tracks correlated with the fraction of time during which they were mobile. Analyses of the single-spot photobleaching and single tracks show that motion blurring is responsible for the observed mobility-intensity correlations in the subpopulation distributions. We conclude that the rapid decrease in the track population with time is governed predominantly by the diffusion of unbound molecules out of the evanescent field.

Filopodia kymographs support the tracking analysis

Cultured HUVECs exhibit numerous filopodia, which are actin-rich tubular extensions >2 μm long and ~150 nm in diameter. Some of the filopodia protruded close to the coverslip and were visualized in our video recordings by the

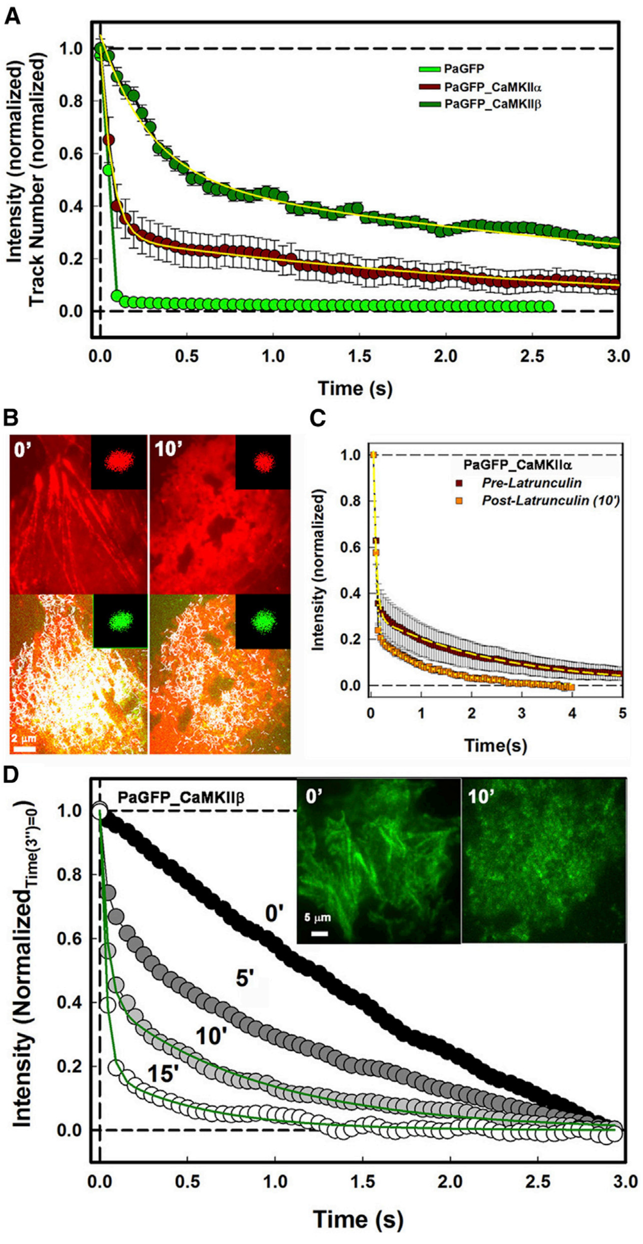

FIGURE 4 Results from photoactivated localization microscopy TIRFM and latrunculin treatment show that both CaMKII isoforms associate with the actin cytoskeleton. (*A*) Normalized fluorescence decay curves of PaGFP and PaGFP-CaMKII fusion proteins after photoactivation by a 405 nm laser (at $t = 0$). Decay was measured as single-molecule track lifetimes. Time 0 is the time required to exceed the five-frame track duration threshold (0.24 s). The data were least-square fitted to two exponentials (*yellow lines*): PaGFP-$\alpha = 0.72 \pm 0.01(e^{(-15.2 \pm 0.5t)}) + 0.28 \pm 0.01(e^{(-0.35 \pm 0.07t)})$, $n = 4306$; PaGFP-$\beta = 0.51 \pm 0.01(e^{(-3.56 \pm 0.08t)}) + 0.49 \pm 0.01(e^{(-0.24 \pm 0.07t)})$, $n = 11{,}160$. In contrast, photoactivated PaGFP fluorescence intensity measured over the image field decayed by >50% within 0.1 s (two frames). (*B*) HUVEC stress fibers after 10 min (10') treatment with latrunculin (5 μM). The top panels (*red*) show the averaged tRFP-actin images: although there is little change in the total fluorescence (97 ± 17 counts/pixel (before latrunculin treatment); 100 ± 23 counts/pixel (after latrunculin treatment)), the fibers disappear after treatment. The bottom panels show PaGFP-CaMKIIα (*green*) and single-particle tracks (*white lines* ($n = 3777$ (0') and 1573 (10')) superimposed on actin (*red*). Insets: FT spectra (tRFP-actin (*red*); GFP (*green*)). (*C*) PaGFP-CaMKIIα fluorescence decay before and after latrunculin treatment. Dual exponential fits: $0.71 \pm 0.01(e^{(-18.1 \pm 0.5t)}) + 0.29 \pm 0.01(e^{(-0.39 \pm 0.01t)})$ (*dashed yellow line*) (0'); $0.8 \pm 0.01(e^{(-19.9 \pm 0.8t)}) + 0.21 \pm 0.01(e^{(-0.92 \pm 0.04t)})$ (*dotted yellow line*) (10'). (*D*) Fluorescence intensity decay curves of PaGFP-β at various times (in minutes) after addition of latrunculin (5 μM) to a Cos7 cell culture. Intensity was normalized to unity at $t = 0$ s (t_0) and zero at $t = 3$ s (t_3); (t_3/t_0) ~50%. Control fit (unnormalized): $(0.26 \pm 0.01) + (0.74 \pm 0.01(e^{(-0.28 \pm 0.01t)})$. Fits after latrunculin treatment (*green lines*): $0.58(e^{(-24.3t)}) + 0.42(e^{(-1.2)})$ (10'); $0.81(e^{(-29.3t)}) + 0.19(e^{(-1.8)})$ (15'). Inset: Filamentous structures visualized when PaGFP-β was photoactivated in the absence of latrunculin (0') were not observed (10') after addition of latrunculin. Correlation coefficient $R^2 > 0.99$ for all fits.

evanescent field excitation. This gave us the opportunity to track GFP-tagged molecules that were essentially constrained to a single dimension independently of the depth, *z*, of the evanescent field. The molecule movements were suitable for kymograph analysis. We straightened the image data by using spline fits to the overall filopodial shape, and then extracted a linear strip of image pixels to form the abscissa in the kymograph time-series image (Fig. S3).

The β complexes produced punctate images on each video frame, and their motion within the filopodium then created a pattern of vertical trajectories (i.e., along the ordinate, time axis). The trajectories consisted of linear, bright segments that were tilted slightly toward the cell body (at ~1.5 μm/min), consistent with complexes binding tightly to actin and reporting the slow rearward flow of the central F-actin bundle of the filopodium (50). These events were interspersed with haphazard, dim trajectories as the particles dissociated from actin and diffused within the body of the filopodium. Both types of trajectories were observed for closely adjacent objects within the same filopodium over the same time window, indicating that dim trajectories result from mobility of the β complexes within the filopodium rather than movement of the filopodium relative to the glass coverslip.

Our initial goal was to achieve a time-resolved characterization of bound and free episodes of β molecules constrained within the evanescent field by the filopodia. However, to our surprise, the kymographs also revealed that both α and β associated with filopodial F-actin.

F-actin dependence of CaMKII α and β lifetime distributions by evanescent field fluorescence photoactivation microscopy

To follow up the finding that both α and β isoforms bind actin within filopodia, we examined the kinetics of fluorescence decay after photoactivation of PaGFP fusion constructs within the cell cortex. A brief flash of TIR laser light at 405 nm was used to activate PaGFP, and continuous illumination at 488 nm allowed the activated fluorescence to be visualized. The fluorescence of PaGFP alone decayed rapidly, reaching half its initial value within a single video frame (<50 ms; Fig. 4 *A*). The decay was two orders of magnitude more rapid than the photobleaching rate

estimated from photobleaching of immobilized GFP molecules or photoactivation of fixed cells (see Materials and Methods). Therefore, the decay must reflect diffusion of the photoactivated PaGFP molecules out of the evanescent field.

The PaGFP-CaMKII fusion constructs (PaGFP-α and PaGFP-β) showed slower and more complex kinetics (Fig. 4 *A*), although it was still rapid relative to photobleaching. Their fluorescence decay could be followed by single-molecule tracking. The decay profiles were approximated by dual-exponential fits with a 0.24 s offset relative to the PaGFP intensity decay due to the five-frame lifetime tracking filter. Direct image field intensity measurements, analogous to those used for PaGFP but corrected for the offset, showed a twofold difference in the fast-component, but not the slow-component, decay. Tracks may terminate for reasons other than fluorescence loss, specifically crossover of tracks of unbound particles and imperfections of the tracking algorithm (Supporting Materials and Methods), that could account for the modest discrepancy.

The slow components for PaGFP-α (1.9 s) and PaGFP-β (3.0 s) were incompatible with free diffusion. Therefore, we used latrunculin B (latrunculin) (51) to test whether disruption of the actin cytoskeleton affected the mobility of PaGFP-CaMKII fusion proteins. The effect of latrunculin on HUVEC stress fibers was evident within a few minutes (Fig. 4 *B*). Before latrunculin treatment, PaGFP-α colocalized weakly, generating an anisotropic pattern that aligned with the stress-fiber arrays as revealed by the elliptical Fourier transform (FT) spectra of the red/green images (red FT ($R_{(maj/min)}$ (major/minor axial ratio)) = 1.35, angle = 16° ± 5°); green FT ($R_{(maj/min)}$ = 1.33, angle = 24° ± 5°). After incubation (10 min) with latrunculin, the pattern had disappeared (FT $R_{(maj/min)}$ = ~1 for both channels; Fig. 4 *B*, *insets*). We measured the photoactivated fluorescence decay kinetics at 0 and 10 min after latrunculin treatment. Dual-exponential fits to the fluorescence decrease showed that the amplitude and rate of the fast-decay component increased with time after treatment, consistent with a reduced F-actin-immobilized fraction (Fig. 4 *C*). We repeated the experiment with PaGFP-β. Photoactivated PaGFP-β formed brightly fluorescent filamentous substructures that disappeared after latrunculin treatment. The kinetics of PaGFP-β fluorescence decay also changed (Fig. 4 *D*) concomitantly with the observed structural changes. The fluorescence decay after photoactivation revealed a substantial fast-decay component for pulses applied 5 min after latrunculin treatment. The fast component increased with incubation time, so for photoactivation pulses 15 min after latrunculin treatment, the decay was similar to that seen for photoactivated PaGFP-α 10 min after latrunculin treatment. The fast-component decay was consistent with the formation of a PaGFP-like inert species.

Structural determinants of the CaMKII F-actin interaction

Having established single-molecule imaging techniques using native α and β isoforms, we next examined the GFP fusions of a panel of functionally significant CaMKII mutants. The mutations are mapped onto the CaMKII structure in Fig. 5 *A* (residue positions are incremented by one in the corresponding β sequence). The primary phosphorylation site, αT286, is important for long-term depression (LTD) as well as LTP since these functions are impaired in <αT286A> mutant mice (52,53) and are affected or abolished, respectively, by overexpression of a constitutively active<αT286D> (54). To explore its role in single-molecule binding to cytoskeletal actin, we studied the homologous βT287A and βT287D mutants (1). Phosphorylation of the secondary sites αT305 and αT306 is known to inhibit kinase activity (33). We compared differences among the αT286D/T305/T306 triple mutants with both secondary sites mutated to either aspartate or alanine. Other mutations/lesions of interest were αK42M, which blocks ATP binding necessary for CaMKII activation, LTP, and spine enlargement (55); αA302R, which disrupts calmodulin binding and translocation to the PSD (9); and the βE′ splice variant, which lacks linker sequences encoded by exons I and IV (56). Finally, we used the tatCN21 inhibitor, which competes with the NR2B NMDA receptor subunit for binding to the T-site (57), to see whether CaMKII binding targets elicit structural changes (58) that affect F-actin association.

The primary phosphorylation site mutants have dramatically different effects on F-actin binding

Averaged images show that the phosphorylation-incompetent βT287A mutant decorates cortical actin structures (Figs. 5 *B*(*i*) and S4). In contrast, the βT287D videos (Movies S2 and S3) show an isotropic distribution of fast-moving spots in the cell cortex that did not map onto the stress fibers (Figs. 5 *B*(*ii*) and S4). As for β, the tracks of immobilized βT287A spots have initial intensities that are several multiples of individual GFP fluorophores and show multistep photobleaching time courses. A rare example of a long-lived track reveals 10 steps (Fig. 5 *C*), consistent with the intensity ratio of the immobilized βT287A spot relative to single GFP fluorophores. In contrast, averaged images of the phosphomimetic βT287D mutant show no evidence of actin colocalization.

The difference between the two mutant proteins was emphasized by an analysis of MSD versus Δt plots (Fig. 5 *D*). For βT287A, the initial slope and subsequent behavior were superimposable with results obtained using native CaMKIIβ. The addition of tatCN21 (1 μM) had no effect on the association of βT287A with F-actin (two different cultures, >10,000 tracks). In contrast, the initial (MSD versus Δt) slope for βT287D was much greater than that for β and βT287A, with virtually no (<7%) tracks of

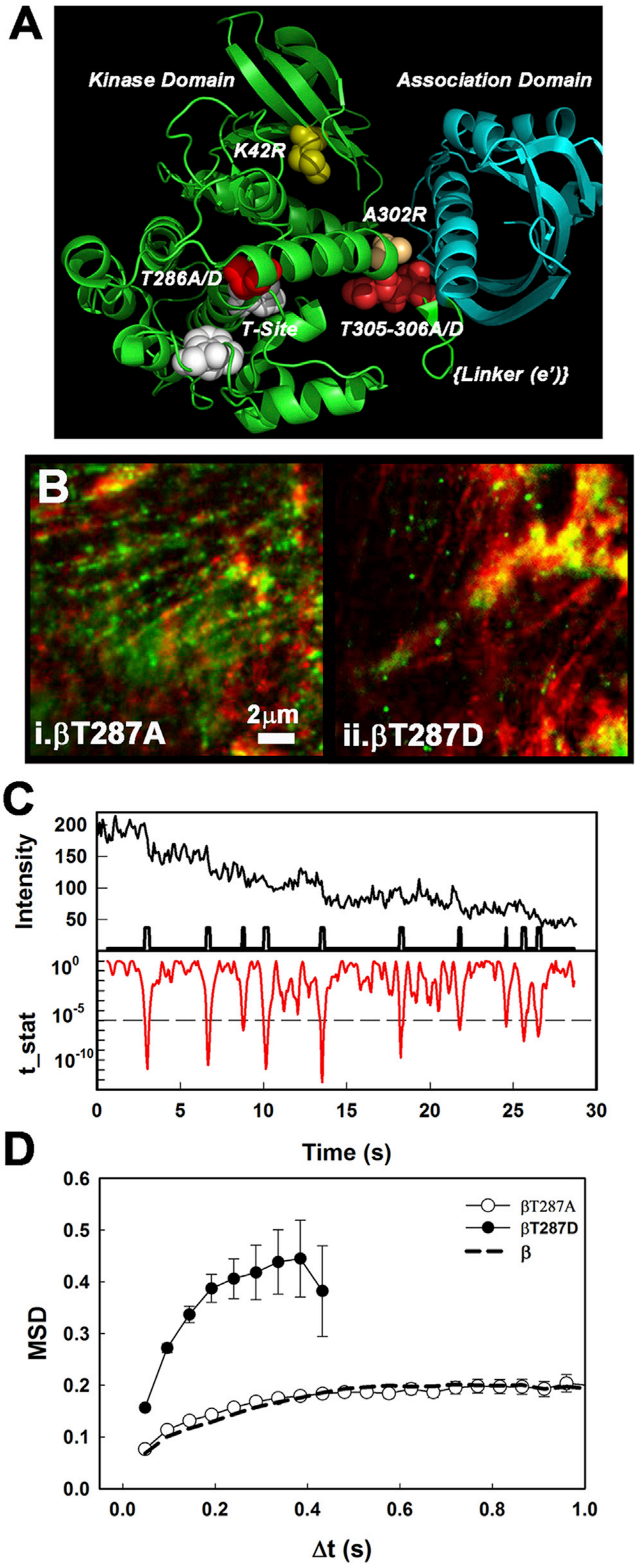

FIGURE 5 The T287D point mutation downregulates association with the actin cytoskeleton. (*A*) Atomic structure (PDB: 3SOA) of rat CaMKII (18). The positions of the mutated sites (K42 (*yellow*), T286 (*red*), A302 (*peach*), and T305/T306 (*magenta*)) studied; the junction of kinase (*green*) and association (*blue*) domains where the splice E′ linker segment would be located; and the substrate-binding T-site (*white*) are shown in relation to the secondary-structure elements (cartoon representation). (*B*) Superimposed averaged images, processed as in Fig. 3 *A*, show localization of single molecules (Fig. S4; Movies S2 and S3) of (*i*) the dephosphorylated mimic, β287A (550 frames, $P_{pix} = 0.29$, $P_{rand} = 0.16 \pm 0.07$, $n = 6511$) and (*ii*) the phosphomimic, β287D (475 frames ($P_{pix} = 0.08$, $P_{rand} = 0.0 \pm 0.08$, $n = 1020$) with tRFP-actin (*red*, 100 frames). The mean tRFP intensities were 105 ± 6 (βT287A) and 213 ± 75 (βT287D) counts/pixel. (*C*) Photobleaching profile of a long-lived β287A-GFP track (*upper black line plot*) with the corresponding *t*-test statistic (*t*-stat) based on a rolling, nonoverlapping 12-frame window (*lower red line plot*). The *t*-stat axis denotes the probability that successive 12-frame segments have the same mean. The probability threshold was set to 10^{-5} for detection of a step change. The bars on the time axis of the upper plot mark the 10 steps identified by the *t*-test. The mean lifetime and intensity decrease per step were 2.6 ± 0.4 s and 15.5 counts/pixel, respectively. (*D*) Average track MSD-versus-Δ*t* plots for the β287D and β287A populations. The dashed line is the plot for the native β population redrawn from Fig. 3 *C*.

duration longer than 0.4s (+0.24 s offset), consistent with fast-moving objects that diffused rapidly out of the evanescent field.

All of the mutant isoforms showed similar single-object intensities but formed two distinct mobility groups

ANOVA was used to test for significant differences in D_{lat} values for the panel of CaMKII mutants (Fig. 6 *A*) based on estimates of variance within and between data sets (Table S1). Variances were normalized for different degrees of freedom, and the probability, *p*, that differences between populations were significant ($p < 0.05$) was computed. Consistent with a visual inspection of the data (Fig. 6 *A*), the results showed two distinct groups: a low-mobility group comprised of the β proteins (native β, βK43R, βA303R, and βT287A) and a high-mobility group comprised of all the α isoform mutants together with βT287D and βE′. A similar pattern was obtained when instantaneous velocities were compared (Fig. S5). The modal, single-spot intensity values (Fig. 6 *B*) obtained across all proteins are similar and vary between 50 and 80 counts/pixel, which is four- to sixfold lower than the anticipated value for the CaMKII holoenzyme and twofold greater than for single GFP fluorophores. The similar values rule out oligomer aggregation as a possible cause of the mobility differences between species. The brighter spots seen decorating stress fibers in some video frames are due to PSF overlap of closely opposed spots; however, their tracks can be separated provided the spots are not stationary (27). Spot intensity measurements suggest that the expression level affects only the holoenzyme number and not the subunit stoichiometry (Fig. S5 *B*). Disassembly is also not the cause of the interspecies mobility differences, since the distributions lack peaks for the single GFP intensity and monomeric α could not be tracked (see "Visualization of homomeric GFP-CaMKIIβ complexes in the cellular cortex" above). The intensity histograms of β, βT287D, and α (Fig. S5 *C*) are differentiated by their skewness rather than their modes. The skewness reflects long-lived track lifetimes and results from oligomer

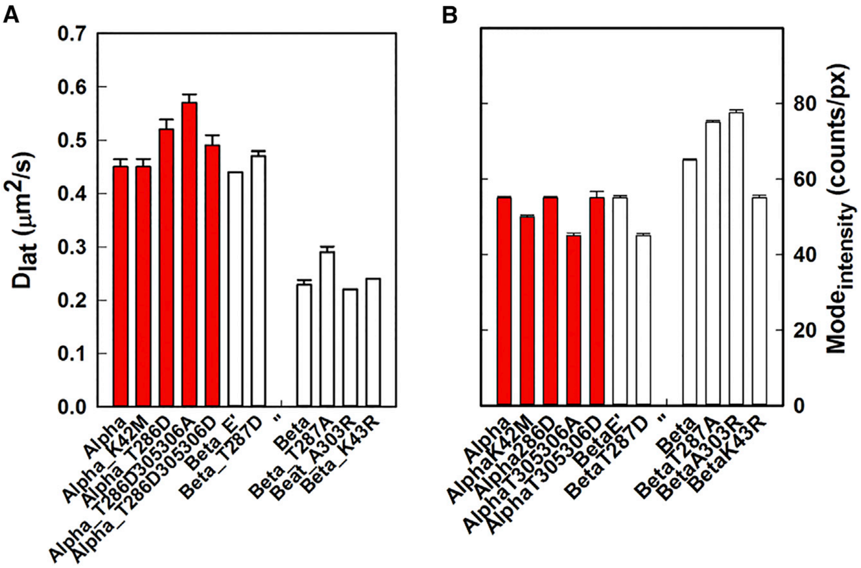

FIGURE 6 Characterization of the mutant proteins. (*A*) Mobility (D_{lat} (mean ± SE)) values for the protein populations. Red bars indicate α-isoforms; white bars indicate β-isoforms. βT287D and βE′ have mobility similar to that of the α proteins. (*B*) Mode (± SE) intensities for the native and mutant GFP-CaMKII fusion protein populations. The bar colors indicate isoforms as in (*A*).

immobilization on actin stress fibers (as shown in Fig. 3, *A* and *B*).

CaMKII dissociation from cytoskeletal actin

Thus far, our analysis indicates that the track lifetimes for both β and α are biphasic, with MSD versus Δt plots of the short-lived population being consistent with diffusion out of the evanescent field (e.g., Figs. 3 *B* and 5 *D*). The photoactivation experiments in the presence and absence of latrunculin demonstrate that the population track lifetime is dramatically reduced coincidently with stress-fiber disassembly. The reduction is mainly due to loss of the long-lived population, implying that these population lifetimes are limited by dissociation from the actin cytoskeleton (Fig. 4). With this in mind, we used track lifetime histograms to estimate the bound fraction and the F-actin dissociation rate for different CaMKII mutants.

The lifetime of the phosphomimic βT287D was taken as representative of unbound molecules, based on βT287D's failure to decorate cytoskeletal structures (Fig. 5 *B*; Movie S3) and its high mobility (Fig. 6 *A*). Consistent with this idea, the βT287D track lifetime data were also fairly monotonic with single exponential decay (rate constant = 6.85 s^{-1} (1–0.05, $R^2 = 0.99$); Fig. S6). We then fitted all of the other track distributions over this range to a function that assumed there was a nonbinding fraction (i.e., like βT287D) and another longer-lived fraction (A_o) that represented actin-binding complexes with an unknown but slower dissociation rate (k):

$$A_t = A_0\left(e^{-kt}\right) + (1 - A_0)\left(e^{-6.85t}\right). \quad (3)$$

The cytoskeletal actin content was assumed to be the same for all experiments, consistent with the modest variation (127 ± 49 counts/pixel) in the mean tRFP-actin intensities in the images (Figs. 3, 4, and 5). The additional information obtained from Eq. 3 is the estimate of the bound (A_0) to freely diffusing pools ($1 - A_0$) of molecules and of the dissociation rate, k, of molecules from the actin cytoskeleton. The bound fraction, A_o, was 0.22 ± 0.02 for all strong-binding β fusion proteins (minus βE′). A_0 was ~2-fold lower for (α) proteins. The overall group pattern was similar to the pattern observed in the D_{lat} analysis. The k-values were 2.9 s^{-1} and 1.3 s^{-1}, respectively, for native (α) and (β) (Fig. S6).

Equation 3 would be valid over the complete (1–0) range only for homogeneous populations that follow single-parameter Poisson probability time distributions. This is not the case for the two populations. For the unbound population, as represented by the βT287D proteins, the D_{lat} value for the most mobile among them (~0.5 μm^2/s; Fig. 6 *A*) was ~18-fold lower than the D_{Stokes} value calculated for free-diffusing (β) holoenzymes (~10 μm^2/s; Eq. 1). This discrepancy, as well as the deviation of the βT287D distribution from the single exponential fit (Fig. S4), indicates hindered diffusion, although bias introduced by exclusion of rapidly diffusing objects by the five-frame (0.24 s) track filter would also contribute. Power-law distributions due to hindered diffusion have been characterized for F-actin gels in vitro (59), as well as in vivo for membrane proteins confined by the actin cortex (60,61). The tRFP-actin labeling does not resolve F-actin single filaments in the dense cortex or F-actin spacing in stress fibers, but limits on physical entrapment may be estimated (Supporting Materials and Methods) to rule out this scenario for stress-fiber decoration. For the bound population, a single k will obtain only if the dissociation of CaMKII from F-actin subunits does not depend on neighboring subunits. This is not the case, since the detachment probability of a subunit will be lower if neighboring subunits participate in binding together the CaMKII holoenzymes and F-actin.

Therefore, we replotted all of our data on log-log axes. We found that they deviated markedly from a dual-exponential process once the population fraction was <5% (Fig. 7 *A*). All distributions showed the same convex log-log relation, consistent with a multiexponential, log-normal distribution of dissociation times. The initial phase of the log plots over which Eq. 3 is valid provides important estimates of the major binding modes. Nevertheless, it was clear that at longer times the data deviated from a single-parameter, two-population model, and this observation was consistent across all data sets.

We compared the times required to reach 10% of the initial amplitude ($t_{1/10}$) between data sets (Fig. 7 *B*) to better represent the log relations. We found the same grouping of different mutants as observed in the D_{lat} analysis. ANOVA (Table S2.I) did not reveal significant differences between the grouped (α) proteins, but did so when these were grouped with βT287D, βE$'$.

As expected, $t_{1/10}$ was lowest for βT287D ($1.14 + 0.02$ s), the reference unbound state, and highest for native β (3.1 ± 0.08 s). We further analyzed differences between the data sets by conducting pairwise *t*-tests against the βT287D reference (Table S2.II) to parse out differences between group members that were not revealed by the ANOVA. The *t*-tests revealed α and αK2M as outliers within the weak-binding group, whereas the $t_{1/10}$ values measured for βE$'$ and the αT286D proteins with and without secondary phosphorylation site mutations were not significantly different from those obtained for βT287D (Fig. 7 *B*).

We used the photoactivation data to estimate the dissociation constants $(K_D)^{app}$ of CaMKII for actin. These data provide a more valid estimate of the actin dissociation rate, k_{off}, since locally activated PaGFP-tagged molecules essentially only leave the evanescent field, whereas GFP-tagged molecules can both exit and enter from the bulk cytoplasm (Supporting Materials and Methods), resulting in a sevenfold difference in the observed decay (Fig. 7). Our $t_{1/10}$ decay rates for PaGFP-α (3.4 ± 0.4 s) and PaGFP-β ($9.4 + 0.2$ s) give k_{off} ($= k_{10}$ ((log (10))/$t_{1/10}$) estimates of 0.68 s^{-1} and 0.23 s^{-1}, respectively. If we assume that the rate of actin association (k_{on}) is in the middle (5×10^5 $M^{-1}s^{-1}$) of the narrow (10^5–10^6 $M^{-1}s^{-1}$) diffusion-controlled range (62) applicable to high-ionic strength media such as cell cytoplasm (63), $(K_D)^{app}$ ($= k_{off}/k_{on}$) is 0.5 μM for β and 1.4 μM for α. The estimate for β is comparable to its measured 2.4 μM affinity for G-actin (24). It is consistent with the simplest scenario of a common binding surface for both G- and F-actin, although more complex scenarios are possible (64,65).

DISCUSSION

In this work, we used TIRFM-based single-molecule imaging experiments, based on dual-color and photoactivation techniques, to study the dynamics of the interactions of CaMKII isotypes with F-actin within live cultured cells. Our ability to detect micromolar-affinity, weak-binding interactions at subsecond resolution provides information that complements classical sedimentation and gel chromatography assays, and leads to important new, to our knowledge, insights.

CaMKII binding to cytoskeletal actin

We conducted mutant analyses to characterize CaMKII binding to cytoskeletal actin. Substitution of the primary phosphorylated threonine residue by aspartate (βT287D and αT286D) abolished F-actin association for both isoforms, whereas substitution with alanine had no effect. The αT286D mutation abrogated affinity for actin and this effect was independent of mutations at the secondary phosphorylation sites. The βT287A and βT287D data are

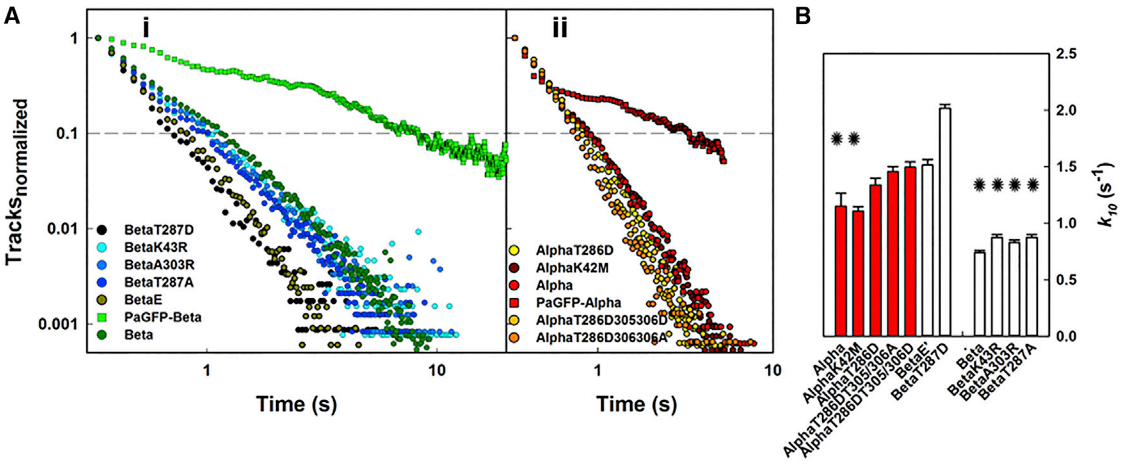

FIGURE 7 Track lifetimes show log-normal decay. (*A*) Log-log plots of the CaMKII track lifetime distributions deviate from dual exponential fits and show a downward curvature that is most evident at longer times (*i*, β proteins; *ii*, α proteins). (*B*) Histogram of rates (k_{10}) computed from decay times to 10% amplitude. Asterisks mark species that associate with F-actin.

consistent with the idea that primary-site phosphorylation acts as a single-stage toggle switch, in line with some activation scenarios (66), to control F-actin binding affinity. Consistent with this idea, the βK43R and αK42M mutations that abolish ATP binding had no effect on F-actin association. Both β and α isoforms should bind ATP in HUVECs, since the CaMKII Michaelis constant for ATP is ~40 μM (58) and the cytoplasmic ATP concentration is typically 2–5 mM (67). In addition, the βK43R/αK42M data show that, in contrast to association with the receptor subunit GluN2B (68), the association with CaMKII F-actin is insensitive to ATP binding and subsequent hydrolysis per se. Elimination of calmodulin binding by the βA303R mutation or use of the peptide inhibitor tatCN21 had no effect on F-actin association. These observations are most simply consistent with low basal Ca^{2+} and CaMKII activity within HUVECs. Finally, the splice segment that is absent in $\beta E'$ is essential for actin binding by the β isoform, consistent with sedimentation assays (22).

Using single-molecule live-cell imaging, we built upon the initial report of stress-fiber decoration in fixed cells (20), which established the CaMKII-F-actin interaction. Our direct observation of actin stress-fiber decoration by immobilized GFP-CaMKII holoenzymes in the presence of a mobile background fraction is consistent with specific binding to F-actin and is incompatible with nonspecific entrapment based on the known stress-fiber architecture. This is also the case for mobility distributions in other actin-rich regions of the cell, based on the known cortical F-actin density and calculated filament mesh size (Supporting Materials and Methods). We found no evidence for higher-order clustering of holoenzymes into larger aggregates that could become either entrapped within or excluded from the actin cortical network or stress fibers. The multistep photobleaching behavior of static spots, along with our histogram analysis of single-object fluorescence intensities, establishes that holoenzymes of CaMKII were the predominant species analyzed in our assays. The presence of larger aggregates is further ruled out by the fact that mutations that abolish Ca^{2+}/CaM binding (A303R) or nucleotide binding (K42M/K43R) required for aggregate formation (36,69) did not alter the native α/β mobility and lifetime distributions. Thus, the mobility differences between the β287D/αT286D proteins and other CaMKII species, as well as the differences between weak- and strong-binding groups analyzed in this study, can only be explained by differences in F-actin binding affinity.

Although the possibility of CaMKII association with other stress-fiber actin-binding proteins (ABPs) cannot be eliminated, three considerations argue for direct association with F-actin. First, the ABP would need to be abundantly and uniformly distributed along the fibers to be consistent with our images (Figs. 3 *A* and 4 *A*; Movies S1 and S2). Other stress-fiber structural ABPs (α-actinin and nonmuscle myosin II) display periodic banding (42). CaMKII binds to α-actinin (70), but this binding is not affected by primary-site phosphorylation (71) and thus may be ruled out. Second, although activated CaMKII has multiple binding targets, there are few binding partners for inactive CaMKII (2), which, as argued above, may be the dominant form in our HUVEC cultures. Third, the relative binding strengths of the CaMKIIβ mutant proteins in our measurements correlate well with results obtained with synthetic F-actin filaments in bundling assays (22).

In neuronal cultures, differences in dendritic arborization (31) and mobility (32) between native and mutant (A303R and K43R) β GFP fusion proteins have been reported, but these differences are probably due to spontaneous neuronal activity that triggers Ca^{2+}.CAM binding for CaMKII activation. Hence, although we do not think that multiple binding partners play a role in our assays, they likely do so in dendritic spines. The reported multiple CaMKII kinetic spine subpopulations (16) may also be due, in part, to multimodal interactions with the F-actin network documented in this study.

Mechanisms for the log-normal bound lifetime distribution

The estimated dissociation constants, $(K_D)^{app}$, for actin are on the order of micromolar for both isoforms. The weak (micromolar) binding of the major β mode is in the ballpark of its reported G-actin affinity. The log-normal distribution of track lifetimes indicates that stronger binding modes exist in addition to the dominant initial mode. These modes may arise from engagement of a variable number of CaMKII subunits with one or more actin filaments (Fig. S7). The fact that the log-normal relation holds for both isoforms and their mutant variants is consistent with the notion that both have a common F-actin binding determinant that is more accessible in β due to its longer, more flexible linker. The alternatively spliced linker region encoded by exons I and IV may increase flexibility between subunits comprising the multimeric complex, thereby ameliorating the geometrical mismatch between CaMKII subunits (72) and binding sites on actin (73). The increased flexibility would optimize contact at the CaMKII and F-actin binding interface and facilitate simultaneous binding at two or more sites, thereby increasing binding avidity. This flexibility could also contribute to heterogeneous binding kinetics, as single-molecule studies indicate that proteins may exist as fluctuating conformational ensembles that lead to power-law distributions in enzyme-turnover experiments over the 10^{-3} to 10 s timescale (74). Phosphorylation or substitution of serine/threonine residues within the linker may also attenuate flexibility, based on differences in residue size and charge, to regulate persistent CaMKII association with F-actin (13). The possibility that the two isoforms have distinct

binding determinants for F-actin cannot be ruled out, but we favor the idea of a common determinant as an explanation based on linker length, which would also account for the difference seen between β and $\beta E'$.

Physiological implications of the log-normal binding curve

The log-normal binding curve extends the concentration range for interaction with the actin cytoskeleton. It has two consequences, as described below:

First, it explains why the temporal resolution of the assay determines the detection sensitivity (Fig. S7). Our ability to detect weak-binding species will decrease as the time resolution of the assay increases. The likely explanation for the inability of classical assays to resolve the weak F-actin binding of $<\alpha>$ is that they only detect long-lived, tight-binding states. The binding of $<\alpha>$ to F-actin, thus established, must have a physiological rationale. Variations in isoform expression ratios occur as neurons develop. The binding ensures that CaMKII holoenzymes, predominantly composed of α subunits, can also target the actin cytoskeleton. Although the noted functional effects of αT286D mutation are thought to be mediated by α kinase activity, F-actin association may also play a role. $<\alpha T286D>$ has reduced synaptic localization (75,76) even though it binds GluN2B in vitro, and loss of F-actin association could account for this effect.

Second, the curve has implications for the transport of $<\beta>$ down neuronal processes. in contrast to $<\alpha>$, $<\beta>$ is expressed only in neuronal cell bodies. Increased avidity due to binding of multiple subunits to F-actin would depend on both the concentration and geometry of F-actin, as well as the multimeric state and flexibility of CaMKII. We estimate expression levels of 0.2–0.4 μM in our assays, based on the density of fluorescent spots in the videos (2000/20 μm^2 image field area) and the 100 nm effective depth of the evanescent field. CaMKII concentrations in neurons are severalfold higher: above ~10 μM holoenzyme in dendritic spines and ~2–5 μM holoenzyme in dendritic processes (5). In regions of the cell where the actin cytoskeleton is sparse (i.e., dendritic and axonal branches (77)), the low (micromolar) affinity of monovalent $<\beta>$ binding would minimize its association with cytoskeletal actin during transport along the long neuronal processes (78). However, in regions where the actin cytoskeleton forms a dense three-dimensional meshwork (i.e., dendritic spines), binding via multiple subunits would be favored and $<\beta>$ would be immobilized. Thus, the extended binding range would facilitate unhindered transport of CaMKII along neuronal processes and sequestration at dendritic spines.

Within dendritic spines, the avidity difference between the two isoforms and between $<\beta>$ and $<\beta E'>$ would increase, with qualitatively different effects on the spine actin cytoskeleton. High avidity mediated by a few β subunits in the $\alpha\beta$ hetero-oligomers might be sufficient to stabilize the dynamic actin cytoskeleton. The $<\beta E'>$ splice variant is expressed in immature neurons (21,31) when affinity for F-actin, which is not required for structural remodeling of synaptic sites, would only hinder the transport needed for targeted kinase activity. Our results provide a quantitative rationale for the fact that the expression of $<\beta E'>$ has different physiological effects compared with that of $<\beta>$.

In conclusion, using single-molecule assays, we were able to resolve CaMKII F-actin binding events on the subsecond-to-second timescale in live mammalian cells. We documented the binding of both neuronal CaMKII isoforms and measured the effect of mutations that act at different points in the CaMKII activation cycle. The behavior of the mutants establishes that binding of CaMKII to actin only occurs when CaMKII is inactive (specifically, when it is not phosphorylated at the primary phosphorylation site). This is in contrast to binding of CaMKII to GluNB, which is triggered only when the kinase is active. This new, to our knowledge, information should be valuable for modeling the role of the actin cytoskeleton in CaMKII transport and synaptic localization.

SUPPORTING MATERIAL

Supporting Materials and Methods, seven figures, two tables, and three movies are available at http://www.biophysj.org/biophysj/supplemental/S0006-3495(16)30440-4.

AUTHOR CONTRIBUTIONS

S.K. designed and performed research, analyzed data, and wrote the manuscript. I.C. performed research. T.C. contributed reagents. K.U.B. designed research, contributed reagents, and wrote the manuscript. J.E.M. designed research, analyzed data, and wrote the manuscript.

ACKNOWLEDGMENTS

We thank Dr. Margaret Stratton for comments on the manuscript, and Dr. Gregory Mashanov for help with computer interfacing and data processing.

This work was supported by grants from the Royal Society Collaborative Exchange (grant U1175), National Institutes of Health (grant R01-NS081248 to K.U.B.), Molecular Biology Consortium (S.K.), Francis Crick Institute which receives its core funding from Cancer Research UK, the UK Medical Research Council, and the Wellcome Trust (J.E.M.). The University of Colorado holds the patent rights for tatCN21, its derivatives, and its uses (PCT/US08/077934, Compositions and Methods for Improved CaMKII Inhibitors and Uses Thereof). K.U.B. is the owner of Neurexus Therapeutics, LLC.

SUPPORTING CITATIONS

References (79–85) appear in the Supporting Material.

REFERENCES

1. Coultrap, S. J., and K. U. Bayer. 2012. CaMKII regulation in information processing and storage. *Trends Neurosci.* 35:607–618.
2. Hell, J. W. 2014. CaMKII: claiming center stage in postsynaptic function and organization. *Neuron.* 81:249–265.
3. Hudmon, A., and H. Schulman. 2002. Neuronal CA2+/calmodulin-dependent protein kinase II: the role of structure and autoregulation in cellular function. *Annu. Rev. Biochem.* 71:473–510.
4. Bayer, K. U., J. Löhler, …, K. Harbers. 1999. Developmental expression of the CaM kinase II isoforms: ubiquitous gamma- and delta-CaM kinase II are the early isoforms and most abundant in the developing nervous system. *Brain Res. Mol. Brain Res.* 70:147–154.
5. Otmakhov, N., and J. Lisman. 2012. Measuring CaMKII concentration in dendritic spines. *J. Neurosci. Methods.* 203:106–114.
6. Borgesius, N. Z., G. M. van Woerden, …, Y. Elgersma. 2011. βCaMKII plays a nonenzymatic role in hippocampal synaptic plasticity and learning by targeting αCaMKII to synapses. *J. Neurosci.* 31:10141–10148.
7. Dosemeci, A., J. H. Tao-Cheng, …, T. S. Reese. 2001. Glutamate-induced transient modification of the postsynaptic density. *Proc. Natl. Acad. Sci. USA.* 98:10428–10432.
8. Shen, K., M. N. Teruel, …, T. Meyer. 2000. Molecular memory by reversible translocation of calcium/calmodulin-dependent protein kinase II. *Nat. Neurosci.* 3:881–886.
9. Shen, K., and T. Meyer. 1999. Dynamic control of CaMKII translocation and localization in hippocampal neurons by NMDA receptor stimulation. *Science.* 284:162–166.
10. Bayer, K. U., and H. Schulman. 2001. Regulation of signal transduction by protein targeting: the case for CaMKII. *Biochem. Biophys. Res. Commun.* 289:917–923.
11. Okamoto, K., T. Nagai, …, Y. Hayashi. 2004. Rapid and persistent modulation of actin dynamics regulates postsynaptic reorganization underlying bidirectional plasticity. *Nat. Neurosci.* 7:1104–1112.
12. Hoffman, L., M. M. Farley, and M. N. Waxham. 2013. Calcium-calmodulin-dependent protein kinase II isoforms differentially impact the dynamics and structure of the actin cytoskeleton. *Biochemistry.* 52:1198–1207.
13. Kim, K., G. Lakhanpal, …, K. Okamoto. 2015. A temporary gating of actin remodeling during synaptic plasticity consists of the interplay between the kinase and structural functions of CaMKII. *Neuron.* 87:813–826.
14. Murakoshi, H., and R. Yasuda. 2012. Postsynaptic signaling during plasticity of dendritic spines. *Trends Neurosci.* 35:135–143.
15. Bayer, K. U., P. De Koninck, …, H. Schulman. 2001. Interaction with the NMDA receptor locks CaMKII in an active conformation. *Nature.* 411:801–805.
16. Lu, H. E., H. D. MacGillavry, …, T. A. Blanpied. 2014. Multiple spatial and kinetic subpopulations of CaMKII in spines and dendrites as resolved by single-molecule tracking PALM. *J. Neurosci.* 34:7600–7610.
17. Tang, F., and K. Kalil. 2005. Netrin-1 induces axon branching in developing cortical neurons by frequency-dependent calcium signaling pathways. *J. Neurosci.* 25:6702–6715.
18. Chao, L. H., M. M. Stratton, …, J. Kuriyan. 2011. A mechanism for tunable autoinhibition in the structure of a human Ca2+/calmodulin-dependent kinase II holoenzyme. *Cell.* 146:732–745.
19. Kanaseki, T., Y. Ikeuchi, …, T. Yamauchi. 1991. Structural features of Ca2+/calmodulin-dependent protein kinase II revealed by electron microscopy. *J. Cell Biol.* 115:1049–1060.
20. Shen, K., M. N. Teruel, …, T. Meyer. 1998. CaMKIIbeta functions as an F-actin targeting module that localizes CaMKIIalpha/beta heterooligomers to dendritic spines. *Neuron.* 21:593–606.
21. Lin, Y. C., and L. Redmond. 2008. CaMKIIbeta binding to stable F-actin in vivo regulates F-actin filament stability. *Proc. Natl. Acad. Sci. USA.* 105:15791–15796.
22. O'Leary, H., E. Lasda, and K. U. Bayer. 2006. CaMKIIbeta association with the actin cytoskeleton is regulated by alternative splicing. *Mol. Biol. Cell.* 17:4656–4665.
23. Okamoto, K., R. Narayanan, …, Y. Hayashi. 2007. The role of CaMKII as an F-actin-bundling protein crucial for maintenance of dendritic spine structure. *Proc. Natl. Acad. Sci. USA.* 104:6418–6423.
24. Sanabria, H., M. T. Swulius, …, M. N. Waxham. 2009. betaCaMKII regulates actin assembly and structure. *J. Biol. Chem.* 284:9770–9780.
25. Wong, A. J., T. D. Pollard, and I. M. Herman. 1983. Actin filament stress fibers in vascular endothelial cells in vivo. *Science.* 219:867–869.
26. Mashanov, G. I., D. Tacon, …, J. E. Molloy. 2003. Visualizing single molecules inside living cells using total internal reflection fluorescence microscopy. *Methods.* 29:142–152.
27. Mashanov, G. I., and J. E. Molloy. 2007. Automatic detection of single fluorophores in live cells. *Biophys. J.* 92:2199–2211.
28. Hern, J. A., A. H. Baig, …, N. J. Birdsall. 2010. Formation and dissociation of M1 muscarinic receptor dimers seen by total internal reflection fluorescence imaging of single molecules. *Proc. Natl. Acad. Sci. USA.* 107:2693–2698.
29. Nenasheva, T. A., G. I. Mashanov, …, J. E. Molloy. 2011. Imaging individual myosin molecules within living cells. *Methods Mol. Biol.* 778:123–142.
30. Nenasheva, T. A., M. Neary, …, J. E. Molloy. 2013. Abundance, distribution, mobility and oligomeric state of M_2 muscarinic acetylcholine receptors in live cardiac muscle. *J. Mol. Cell. Cardiol.* 57:129–136.
31. Fink, C. C., K. U. Bayer, …, T. Meyer. 2003. Selective regulation of neurite extension and synapse formation by the beta but not the alpha isoform of CaMKII. *Neuron.* 39:283–297.
32. Khan, S., T. S. Reese, …, A. Shabbir. 2012. Spatiotemporal maps of CaMKII in dendritic spines. *J. Comput. Neurosci.* 33:123–139.
33. Pi, H. J., N. Otmakhov, …, J. Lisman. 2010. Autonomous CaMKII can promote either long-term potentiation or long-term depression, depending on the state of T305/T306 phosphorylation. *J. Neurosci.* 30:8704–8709.
34. Bayer, K. U., E. LeBel, …, P. De Koninck. 2006. Transition from reversible to persistent binding of CaMKII to postsynaptic sites and NR2B. *J. Neurosci.* 26:1164–1174.
35. Otmakhov, N., J. H. Tao-Cheng, …, J. Lisman. 2004. Persistent accumulation of calcium/calmodulin-dependent protein kinase II in dendritic spines after induction of NMDA receptor-dependent chemical long-term potentiation. *J. Neurosci.* 24:9324–9331.
36. Hudmon, A., E. Lebel, …, P. De Koninck. 2005. A mechanism for Ca2+/calmodulin-dependent protein kinase II clustering at synaptic and nonsynaptic sites based on self-association. *J. Neurosci.* 25:6971–6983.
37. Subach, F. V., G. H. Patterson, …, V. V. Verkhusha. 2009. Photoactivatable mCherry for high-resolution two-color fluorescence microscopy. *Nat. Methods.* 6:153–159.
38. Merzlyak, E. M., J. Goedhart, …, D. M. Chudakov. 2007. Bright monomeric red fluorescent protein with an extended fluorescence lifetime. *Nat. Methods.* 4:555–557.
39. Goedhart, J., D. von Stetten, …, A. Royant. 2012. Structure-guided evolution of cyan fluorescent proteins towards a quantum yield of 93%. *Nat. Commun.* 3:751.
40. Conte, I. L., N. Hellen, …, T. Carter. 2016. Interaction between MyRIP and the actin cytoskeleton regulates Weibel-Palade body trafficking and exocytosis. *J. Cell Sci.* 129:592–603.
41. Khan, S., Y. Zou, …, T. S. Reese. 2011. Sequestration of CaMKII in dendritic spines in silico. *J. Comput. Neurosci.* 31:581–594.
42. Pellegrin, S., and H. Mellor. 2007. Actin stress fibres. *J. Cell Sci.* 120:3491–3499.
43. Burnette, D. T., S. Manley, …, J. Lippincott-Schwartz. 2011. A role for actin arcs in the leading-edge advance of migrating cells. *Nat. Cell Biol.* 13:371–381.

44. Wang, Z., R. Ginnan, …, H. A. Singer. 2010. Calcium/calmodulin-dependent protein kinase II delta 6 (CaMKIIdelta6) and RhoA involvement in thrombin-induced endothelial barrier dysfunction. *J. Biol. Chem.* 285:21303–21312.
45. Dalgaard, P. 2008. Introductory Statistics with R, 2nd ed. Springer, New York.
46. Fischer, H., I. Polikarpov, and A. F. Craievich. 2004. Average protein density is a molecular-weight-dependent function. *Protein Sci.* 13:2825–2828.
47. Swaminathan, R., C. P. Hoang, and A. S. Verkman. 1997. Photobleaching recovery and anisotropy decay of green fluorescent protein GFP-S65T in solution and cells: cytoplasmic viscosity probed by green fluorescent protein translational and rotational diffusion. *Biophys. J.* 72:1900–1907.
48. Swaminathan, R., S. Bicknese, …, A. S. Verkman. 1996. Cytoplasmic viscosity near the cell plasma membrane: translational diffusion of a small fluorescent solute measured by total internal reflection-fluorescence photobleaching recovery. *Biophys. J.* 71:1140–1151.
49. Rellos, P., A. C. Pike, …, S. Knapp. 2010. Structure of the CaMKII-delta/calmodulin complex reveals the molecular mechanism of CaMKII kinase activation. *PLoS Biol.* 8:e1000426.
50. Kerber, M. L., D. T. Jacobs, …, R. E. Cheney. 2009. A novel form of motility in filopodia revealed by imaging myosin-X at the single-molecule level. *Curr. Biol.* 19:967–973.
51. Coué, M., S. L. Brenner, …, E. D. Korn. 1987. Inhibition of actin polymerization by latrunculin A. *FEBS Lett.* 213:316–318.
52. Giese, K. P., N. B. Fedorov, …, A. J. Silva. 1998. Autophosphorylation at Thr286 of the alpha calcium-calmodulin kinase II in LTP and learning. *Science.* 279:870–873.
53. Coultrap, S. J., R. K. Freund, …, K. U. Bayer. 2014. Autonomous CaMKII mediates both LTP and LTD using a mechanism for differential substrate site selection. *Cell Reports.* 6:431–437.
54. Mayford, M., M. E. Bach, …, E. R. Kandel. 1996. Control of memory formation through regulated expression of a CaMKII transgene. *Science.* 274:1678–1683.
55. Yamagata, Y., S. Kobayashi, …, S. Okabe. 2009. Kinase-dead knock-in mouse reveals an essential role of kinase activity of Ca2+/calmodulin-dependent protein kinase IIalpha in dendritic spine enlargement, long-term potentiation, and learning. *J. Neurosci.* 29:7607–7618.
56. Brocke, L., M. Srinivasan, and H. Schulman. 1995. Developmental and regional expression of multifunctional Ca2+/calmodulin-dependent protein kinase isoforms in rat brain. *J. Neurosci.* 15:6797–6808.
57. Vest, R. S., K. D. Davies, …, K. U. Bayer. 2007. Dual mechanism of a natural CaMKII inhibitor. *Mol. Biol. Cell.* 18:5024–5033.
58. Raveendran, R., S. Devi Suma Priya, …, R. V. Omkumar. 2009. Phosphorylation status of the NR2B subunit of NMDA receptor regulates its interaction with calcium/calmodulin-dependent protein kinase II. *J. Neurochem.* 110:92–105.
59. Schmidt, C. F., M. Barmann, …, E. Sackmann. 1989. Chain dynamics, mesh size and diffusive transport in networks of polymerized actin. A quasielastic light scattering and microfluorescence study. *Macromolecules.* 22:3638–3649.
60. Heinemann, F., S. K. Vogel, and P. Schwille. 2013. Lateral membrane diffusion modulated by a minimal actin cortex. *Biophys. J.* 104:1465–1475.
61. Umemura, Y. M., M. Vrljic, …, A. Kusumi. 2008. Both MHC class II and its GPI-anchored form undergo hop diffusion as observed by single-molecule tracking. *Biophys. J.* 95:435–450.
62. Schlosshauer, M., and D. Baker. 2004. Realistic protein-protein association rates from a simple diffusional model neglecting long-range interactions, free energy barriers, and landscape ruggedness. *Protein Sci.* 13:1660–1669.
63. Fersht, A. 1999. Structure and Mechanism in Protein Science. W.H. Freeman & Co., New York.
64. Dominguez, R. 2004. Actin-binding proteins—a unifying hypothesis. *Trends Biochem. Sci.* 29:572–578.
65. dos Remedios, C. G., D. Chhabra, …, N. J. Nosworthy. 2003. Actin binding proteins: regulation of cytoskeletal microfilaments. *Physiol. Rev.* 83:433–473.
66. Thaler, C., S. V. Koushik, …, S. S. Vogel. 2009. Structural rearrangement of CaMKIIalpha catalytic domains encodes activation. *Proc. Natl. Acad. Sci. USA.* 106:6369–6374.
67. Sachs, J. R. 1981. Internal potassium stimulates the sodium-potassium pump by increasing cell ATP concentration. *J. Physiol.* 319:515–528.
68. O'Leary, H., W. H. Liu, …, K. U. Bayer. 2011. Nucleotides and phosphorylation bi-directionally modulate Ca2+/calmodulin-dependent protein kinase II (CaMKII) binding to the N-methyl-D-aspartate (NMDA) receptor subunit GluN2B. *J. Biol. Chem.* 286:31272–31281.
69. Vest, R. S., H. O'Leary, and K. U. Bayer. 2009. Differential regulation by ATP versus ADP further links CaMKII aggregation to ischemic conditions. *FEBS Lett.* 583:3577–3581.
70. Walikonis, R. S., A. Oguni, …, M. B. Kennedy. 2001. Densin-180 forms a ternary complex with the (alpha)-subunit of Ca2+/calmodulin-dependent protein kinase II and (alpha)-actinin. *J. Neurosci.* 21:423–433.
71. Robison, A. J., R. K. Bartlett, …, R. J. Colbran. 2005. Differential modulation of Ca2+/calmodulin-dependent protein kinase II activity by regulated interactions with N-methyl-D-aspartate receptor NR2B subunits and alpha-actinin. *J. Biol. Chem.* 280:39316–39323.
72. Rosenberg, O. S., S. Deindl, …, J. Kuriyan. 2006. Oligomerization states of the association domain and the holoenyzme of Ca2+/CaM kinase II. *FEBS J.* 273:682–694.
73. Wu, Y., and J. Ma. 2004. Refinement of F-actin model against fiber diffraction data by long-range normal modes. *Biophys. J.* 86:116–124.
74. Min, W., and X. S. Xie. 2006. Kramers model with a power-law friction kernel: dispersed kinetics and dynamic disorder of biochemical reactions. *Phys. Rev. E Stat. Nonlin. Soft Matter Phys.* 73:010902.
75. Marsden, K. C., A. Shemesh, …, R. C. Carroll. 2010. Selective translocation of Ca2+/calmodulin protein kinase IIalpha (CaMKIIalpha) to inhibitory synapses. *Proc. Natl. Acad. Sci. USA.* 107:20559–20564.
76. Barcomb, K., I. Buard, …, K. U. Bayer. 2014. Autonomous CaMKII requires further stimulation by Ca2+/calmodulin for enhancing synaptic strength. *FASEB J.* 28:3810–3819.
77. Xu, K., G. Zhong, and X. Zhuang. 2013. Actin, spectrin, and associated proteins form a periodic cytoskeletal structure in axons. *Science.* 339:452–456.
78. Adamec, R., M. Hebert, …, R. F. Mervis. 2012. Dendritic morphology of amygdala and hippocampal neurons in more and less predator stress responsive rats and more and less spontaneously anxious handled controls. *Behav. Brain Res.* 226:133–146.
79. Patterson, G. H. 2007. Photobleaching and photoactivation of fluorescent proteins for studies in cell biology. *Microsc. Microanal.* 13:294–295.
80. Schneider, C. A., W. S. Rasband, and K. W. Eliceiri. 2012. NIH Image to ImageJ: 25 years of image analysis. *Nat. Methods.* 9:671–675.
81. Manders, E. M., F. J. Verbeek, and J. A. Aten. 1993. Measurement of co-localization of objects in dual-colour confocal images. *J. Microsc.* 169:375–382.
82. Costes, S. V., D. Daelemans, …, S. Lockett. 2004. Automatic and quantitative measurement of protein-protein colocalization in live cells. *Biophys. J.* 86:3993–4003.
83. Qian, H., M. P. Sheetz, and E. L. Elson. 1991. Single particle tracking. Analysis of diffusion and flow in two-dimensional systems. *Biophys. J.* 60:910–921.
84. Meyer, R. K., and U. Aebi. 1990. Bundling of actin filaments by alpha-actinin depends on its molecular length. *J. Cell Biol.* 110:2013–2024.
85. Koestler, S. A., K. Rottner, …, J. V. Small. 2009. F- and G-actin concentrations in lamellipodia of moving cells. *PLoS One.* 4:e4810.

Biophysical Journal, Volume *111*

Supplemental Information

Multiple CaMKII Binding Modes to the Actin Cytoskeleton Revealed by Single-Molecule Imaging

Shahid Khan, Ianina Conte, Tom Carter, K. Ulrich Bayer, and Justin E. Molloy

SUPPORTING MATERIAL

A. Assay Development

(1) Total Internal Reflection Fluorescence Microscopy (TIRFM) – The microscope workstation was mounted on a vibration isolation table (*Isostation*, Newport Spectra-Physics Ltd., Didcot, UK). Laboratory and instrument temperature was maintained at 23.5^{o}C.

Stage movement in x,y planes and objective focus in the z-direction was effected with nanometer precision by piezoelectric positioners (*MCS-3D*, SmarAct GmbH, Oldenburg, Germany and *PIfoc P-721.10*, Physik Instrumente Ltd, Bedford, UK, respectively). The workstation was equipped with four lasers (*Crystal Laser CL-2000* (50mW @ 405nm) Reno NV, USA; *Protera A488-15*, (30mW @ 488nm) Laser2000, Huntingdon, Cambridge, UK; *DPGL-2200*, (200mW @ 532nm) Suwtech, Shanghai, China; *MGL-FN-561*, (200mW @ 561nm) Changchun New Industries Co. Ltd., Changchun, China). The laser beams were co-aligned and bought into total internal reflection (TIR) mode by a system of mirrors and lenses mounted on a combination of translation mounts and kinematic mounts (Thorlabs Inc., Newton NJ, USA). Laser intensities were modulated by variable neutral density filters (*NDC50C2M*, Thorlabs Inc.) or by varying the laser supply current. Power output was measured and calibrated using a laser power meter (*LaserMate-Q*, Coherent, Santa Clara, CA, USA). Laser powers were measured at the point where the laser beam exited the final objective lens. Lasers were blocked either by TTL modulation of the laser power supplies or by electronic shutter (Uniblitz D122, Vincent Associates, Rochester NY, USA).

Evanescent field excitation was achieved by focusing the laser beams at the extreme edge of the back aperture of a high-numerical aperture objective lens (*PlanApo 100x, 1.49NA*, Olympus, Southend-on-Sea, UK) to produce a collimated beam of light that emerged from the objective front face at an angle that exceeded the critical angle (>63°). Index-matched immersion oil (*Immersol 518*, Zeiss, Cambridge, UK) coupled the objective lens to the glass-bottomed culture dish (Lab-Tek, Nunc, Rochester, NY, USA). Filter cubes, mounted in the microscope's epifluorescence filter wheel, separated the eGFP fluorescence (Excite 488nm Laser, dichroic: Di02-R488; Emission filter: FF02-520/28, Semrock, Rochester, NY, USA) from the tagRFP fluorescence (Excite 561nm laser, dichroic: Di02-R561; Emission filter: FF01-609/54, Semrock).

For photo-activation studies, cells were co-transfected with tRFP-actin and a PaGFP-CAMKII fusion protein. Control cells were transfected with PaGFP. Transfected cells were first located by visualization based on their tRFP-actin fluorescence. Photo-activation occurred within one video frame and then increased during the duration of the flash, determined by a balance between the rate at which fluorophores were generated by photo-activation and lost by diffusion from the evanescent field and by photo-bleaching. Fluorophore excitation in the z-axis is determined by the evanescent field decay distance (1/e ~ 100 nm). Consistent with this interpretation, termination of the photo-activation flashes resulted in a rapid decay of fluorescence signal back to the starting level. Fluorescence changes were averaged over several cycles to obtain the rise and decay kinetics. For each genetic construct the mean decay profile was obtained by pooling video-records from different experiments (n >= 2). Photo-bleaching rate determined from photo-activation of a cell culture fixed with 1% paraformaldehyde was < 10% in 10 seconds. PaGFP and GFP photo-bleaching rates are similar (1).

The sample fluorescence was imaged by an electron-multiplying, EMCCD, camera (iXon-897bv, Andor, Belfast, UK) camera at 96 nm/pixel magnification and 20 fps readout of the full image field (512x512 pixels). Two-fold higher frame rates were achieved when desired by reducing the pixel readout area. Input to the laser shutters and image acquisition were controlled by a personal computer with custom software. Video data was stored on computer hard disc. Images were subsequently analyzed using either GMimPro (2) or ImageJ (3).

Live cells are more challenging than fixed cells for co-localization analysis. In fixed cells movements of cellular sub-structures or loosely-attached membrane relative to the glass coverslip

are not issues. Loss of soluble contents during fixation eliminates the high background in the red channel due to G-actin and labile F-actin microfilaments. In the TIRFM images, spatial differences due to cell body versus periphery, inhomogeneous cell attachment to the cover-glass and inexact coincidence of the red / green laser beam profiles will additionally be aggravated by the high camera gain required for single-molecule tracking. In addition, stress fiber labeling by RFP-actin (red channel) is punctate due to its low expression level compared to endogenous actin, while single molecule imaging (green channel) is inherently stochastic. Co-localization of red / green channel pixel intensities from selected quadrants, reported as the Pearson's correlation coefficient (P_{pix}), was computed using the Image J Manders coefficient plugin. The images were background subtracted and quadrants chosen to maximize the signal from the stress fiber array and minimize noise due to spatial inhomogeneity. Scatter plots gave an objective sense of co-localized pixel frequencies relative to non-overlapping pixels, as described (4). To assess significance the green (GFP) image was scrambled by shuffling pixel blocks to generate 25 randomized images. P_{pix} was then compared against the mean ($\pm\sigma$) correlation (P_{rand}) of the randomized images with the red (tRFP-actin) image (5).

(2) Single Molecule Image Analysis - The point spread function (PSF) of a single fluorophore in the object plane (here termed the x,y-plane), has a full-width at half-maximum (FWHM) of ~ 0.3 µm. At the 0.1 µm / pixel magnification of our imaging system, 95% of the PSF intensity is encompassed by a 3x3 square pixel region. An automatic single particle tracking (ASPT) algorithm, described previously (2) was used to detect fluorescent spots in successive video images based on convolution of a moving kernel of an idealized 2-dimensional PSF. Fluorescent spots, identified on the basis of their match to the idealized PSF are reinforced relative to other regions of the image. Following spot detection, the intensity of the spot was computed by taking the mean intensity of the pixels within the 3x3 pixel region surrounding the spot centroid less the intensity of the adjacent ring of pixels. The latter operation gave a local back-ground subtraction. The detected spot x-y coordinates were then determined with sub-pixel resolution by fitting the PSF to a 2-D Gaussian function. Spot locations were then tracked in successive video frames by linking object coordinates using a nearest-neighbor algorithm, to give object "tracks" consisting of a series of x-y coordinates, time and intensity. An intensity cut-off eliminated spots whose brightness was 20X that expected for single GFP fluorophores. A one-frame look up function minimized fragmentation of a trajectory of a fluorescent spot into multiple tracks. Fragmentation could be due to frame-by-frame displacements larger than allowed by the search area chosen to assign centroids in adjacent frames or to electronic camera noise. The object tracks were then further analyzed using either custom software, Excel (Microsoft Corp, Redmond, WA), IgorPro (Wavemetrics, Lake Oswego, OR, USA) or MatLab (Mathworks, Cambridge, Cambs, UK). Least squares fits based on the Kolmogorov-Smirnov normality test were performed in Sigmaplot 12.0 (Systat software, San Jose, CA, USA).

Our estimation of the lateral diffusion coefficient, D_{lat}, is derived from the initial gradient of mean squared displacement (MSD) versus time interval (Δt) plots. This assumes MSD increases linearly with Δt (i.e. the objects exhibit "free-diffusion", characterized by a Brownian walk). Although this assumption is not strictly valid, the estimated D_{lat} has comparative value as long as any anomalous behavior scales linearly, which seems a fair approximation. Although mobility at the plasma membrane is essentially constrained in two-dimensions, diffusion within the cytosol is three-dimensional and can be estimated from the two-dimensional projection of the object's motion, applying a linear, $2/3^{rds}$ scaling factor. The averaged particle speeds, V_{avg}, are normally distributed (as expected, from the central limit theorem).

(3) In situ photo-bleaching of immobilized tracks – The ability to record bound lifetimes of single GFP-CaMKII holoenzymes rests on the fact that photo-bleaching of multiple fluorophores takes many seconds. T-tests (**Fig. S1**) show that GFP-CaMKII intensity jumps (Figure 3D) have similar frequency

but smaller fractional intensity drops per step (Results Section A4) in comparison to tracks of immobilized GFP molecules (Figure 2B),.

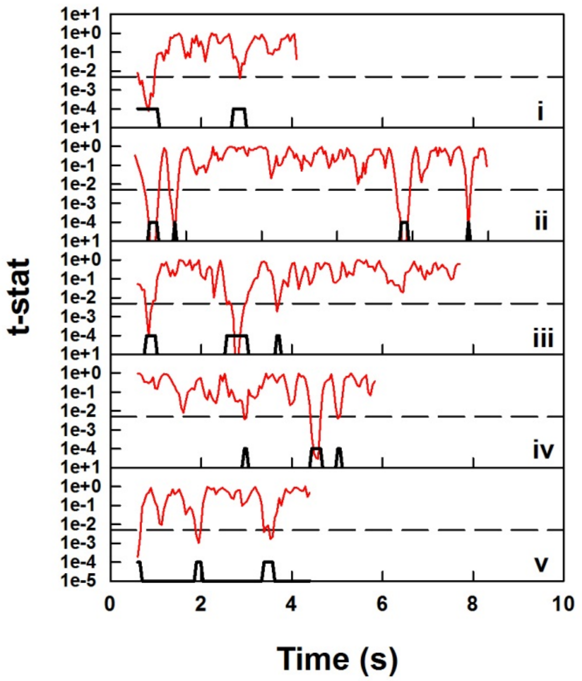

Fig. S1: Step photo-bleaching *- T-test statistics (red lines) for the track intensity profiles in Figure 3D. The t-test statistic records the probability that the mean intensities of adjacent twelve frame segments were the same. Intensity jumps (black markers) were identified when the probability was <* $10^{-2.5}$ *(dashed lines).*

(4) The relation between intensity, D_{lat} ***and track lifetimes*** – The intensity, duration and mobility of six single tracks, selected from the population distribution studied in Figure 3, were analysed (**Fig. S2A**). The frame centroid intensity determined from the tracking algorithm, is computed by local background subtraction. It varies inversely with the frame-to-frame displacement being lower when molecules are unbound and their fluorescence is distributed over a larger area due to diffusion. The form of the population-averaged MSD-Δt plot reconstructed from plots of the individual tracks is governed by differences in track lifetimes. At low Δt values it is governed by a mean D_{lat} representative of the mobility of all spots. At high Δt values, its form is governed by the decrease in population size as the more mobile spots exit the evanescent field and the immobile, long-lived spots dominate as illustrated by the six-track population plot.

The complete, population distribution is shown (**Fig. S2B**) to identify how the sub-populations (Figures 3B, C) were selected based on their lifetimes. There will be a continuum between the mobility of unbound versus tightly bound particles if population size is sufficiently large (> 10^3). The population curves (Figures 3B, 5D) for the short lived tracks (Fig. S2B yellow bar) and the complete population may be reconstructed based on simple equations, to a good approximation, to sharpen intuition.

For short tracks,

$$\overline{MSD_{t=1}^{n}} = 4\{\sum_{t=1}^{n}(Tracks.D_{lat})/\sum_{t=1}^{n}Tracks\}.t \qquad \mathbf{A}$$

For the complete population

$$\overline{MSD_{t=1}^{n}} = 4\{(\sum Tracks).\overline{D_{lat}} - \sum_{t=0}^{n-1}(Tracks.D_{lat})\}/\{(\sum Tracks) - \sum_{t=0}^{n-1} Tracks\}. \quad \mathbf{B}$$

The population curve for the short tracks generated by equation-A mirrors the curve shown in Figure 3B well as the plots of the individual tracks are close to the linear MSD versus Δt relation assumed in the equations. This is not the case for the long-lived tracks as seen from tracks 2 and 4 (Fig. S2A). The deviation from the assumption results in a discrepancy in the rise and decay amplitudes between the real curve and the curves generated by equation-B. Nevertheless the basic characteristics of the initial rise, peak and subsequent decay are reproduced.

Kymographs of molecules confined in filopodia (**Fig. S3**) provide visual illustration of the relation between intensity and D_{lat} for single tracks constrained to move in the (x, y) plane. The mean intensity of the kymograph tracks Q =Σq/N where N is the number of frames.

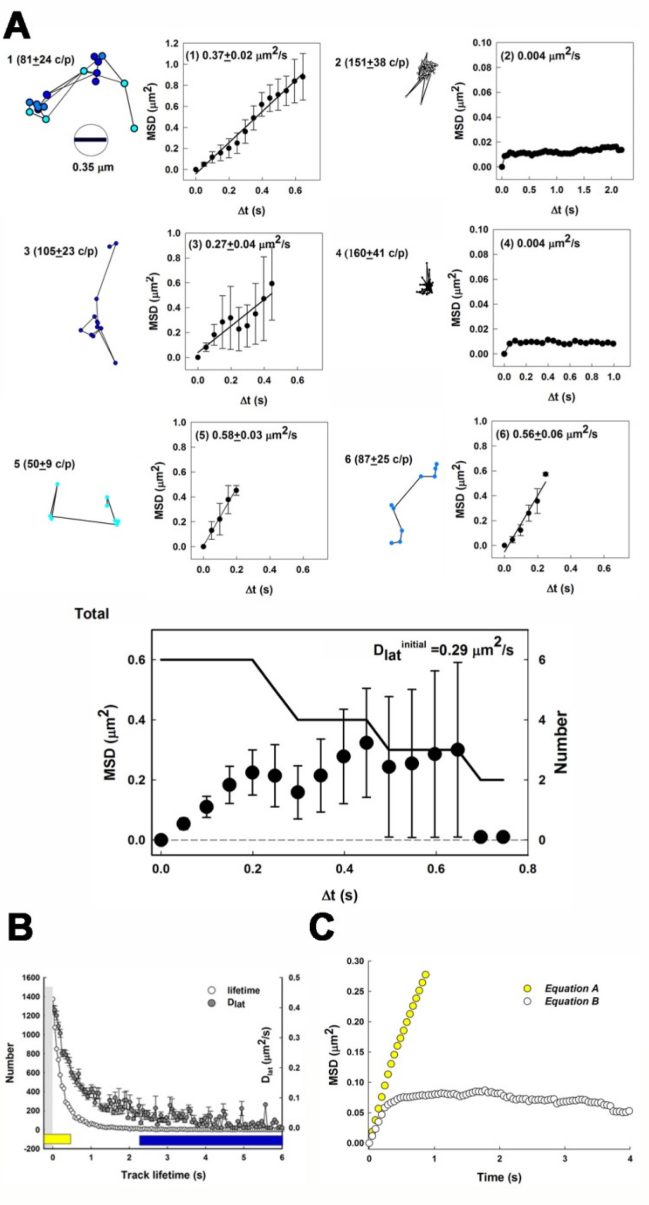

***Fig. S2: Properties of single tracks and their population MSD-Δt plot –A.** Panels show single tracks linking centroids in successive frames (left) and the resulting MSD plots (right). Centroid colour reflects intensity (cyan (40-65 counts / pixel), blue (70 – 95 counts / pixel), dark-blue (100-125 counts / pixel), black (140-165 counts / pixel)). Mean track intensities and derived D_{lat} values are listed. Error*

bars for single track MSD plots were computed following (6). Error bars for the population plot (black symbols) reflect the variation in population size. Dips in the plot mirror the decreases in the number of tracks (solid line). At Δt = 0.7 s, MSD drops to the value (0.004 μm^2) for the 2 immobilized tracks. The value reflects the positional uncertainty in centroid determination. ***B.*** *The decrease in the size of the complete population (Figure 3) with time. Associated changes in D_{lat} ($\pm$ σ) values are shown as a histogram, binned according to lifetime. Grey vertical bar is 5-frame lifetime track filter The Figure 3B plots for the selected sub-populations (yellow (short tracks); blue (long-tracks)) illustrate the D_{lat} changes.* ***C.*** *MSD-Δt plots generated from the population histogram (B) by equation A (short-lived tracks (yellow)) and equation B (complete population (white))*

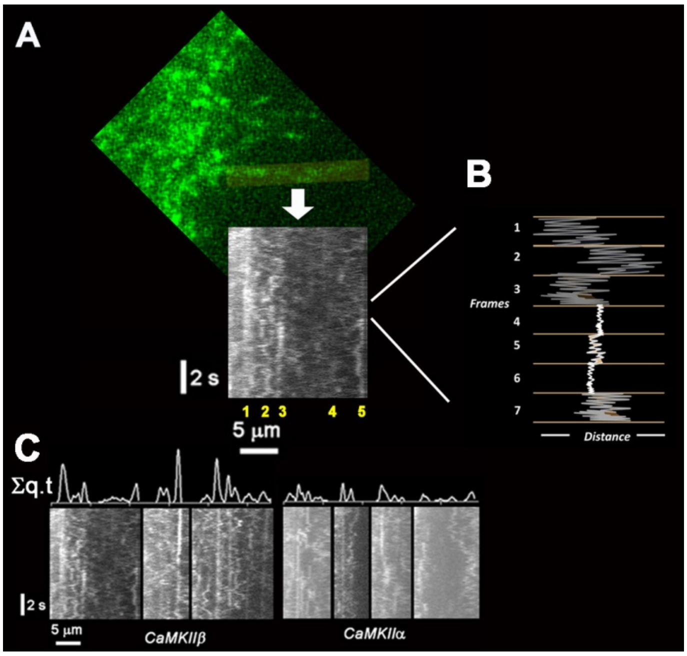

Fig.S3: CaMKII molecules in filopodia -A*. Creation of a kymograph. A single frame image of a filopodium (highlighted) was skeletonized, the skeletonized outline padded (5 pixels), then computationally straightened. The intensity along the length of the filopodium was measured with ImageJ. The stack of intensity profiles obtained from several frames formed a kymograph (length (abscissa), time (ordinate)). Bound or weakly bound molecules persist at one position along the filopodium generating a track in the stack. 5 tracks (yellow numerals) are seen in the kymograph. Tracks 3 and 5 have bright linear segments indicative of bound states and dim wavy segments indicative of unbound, diffusive states. Track 1 is predominantly bound. Track 4 is predominantly unbound.* ***B.*** *Schematic over seven frames of a segment from track 5 shows displacements within frames (δt = 50ms). The fluorescence image of a freely diffusing unbound molecule will be spread over an area greater than that occupied by its PSF when bound and immobilized. Colour (white = high, black = low) denotes intensity, q, of the computed centroid for each frame.* ***C.*** *Kymographs of native GFP-α and GFP-β homomeric complexes visualised within filopodia of HUVECs. The intensity profiles were summed over frames (white lines above the kymographs). The summed pixel intensities $(\Sigma qt)_x$ were fitted to Gaussian curves of form $(\Sigma qt)_x = a(exp(0.5((x-x_0)/b)^2))$, where "a" is the peak amplitude (counts/pixel), "x_0" the position of the peak along the filopodium, and "b" the distance x - x_0 (pixels) where the intensity is 1/e of the peak value. β and α have average (a / b) values of 23.5 / 4.4 and 10.7 / 3.8 respectively.*

B. Mutant Analyses

(1) The GFP-CaMKII fusion proteins form 2 distinct groups based on mobility – Co-localization of the proteins βT287A, βK43R and βA303R with stress fibres is comparable to β (β_{strong} group). In contrast, βT287D does not co-localize with the fibres (**Fig. S4**). ANOVA shows that α and β_{strong} form two distinct groups (**Table S1**). Mobility, as measured by V_{avg} has similar trend as measured from D_{lat} values. The intensity distributions are distinguished by their skewness. The skewness is due to long-lived tracks (Fig. S2). Control β populations have comparable mobility, independent of tRFP-actin (D_{lat} = 0.23±0.02 μm^2/s (Figure 6A) (+), 0.21 μm^2/s (-)) (**Fig. S5**).

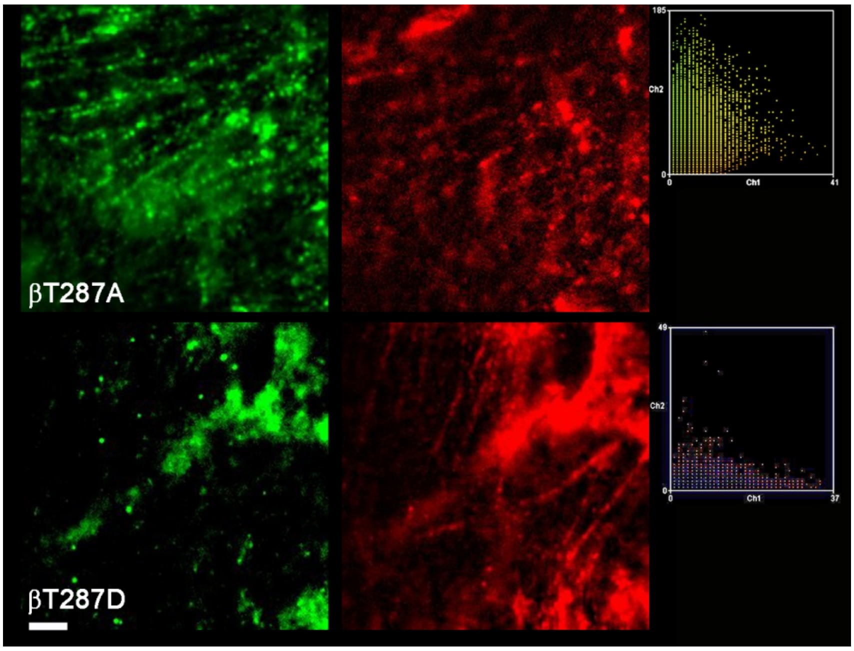

Fig. S4: Co-localization of βT286 point mutant proteins – *Left: Selected quadrants from the full image fields seen in Movies S2 (βT287A) and S3 (βT287D). The red and green images were merged for composite images in Figure 5B. Right: Scatter plots display pixel intensities with values along the diagonal indicating overlap, and non-diagonal values adjacent to the ordinate (ch 1: red) or abscissa (ch2: green) non-overlap. The plots give a visual representation of the overlap measured by the P_{pix} coefficients (Figure 5B).*

Data-Set	*Df_B | Df_T*	*F-Value*	*p-Value*
D_{lat}(Total)	10|101	21.24	2.2xe^{-16}
D_{lat}(α)	3|27	1.98	0.125
D_{lat}(β)	5|74	17.28	2.7xe^{-11}
D_{lat}(β_{strong})	3|78	1.31	0.276

***Table S1: Mobility statistics.** Datasets drawn from different cultures for each GFP-CaMKII fusion were grouped (Total = all GFP-CaMKII proteins, α = GFP-CaMKIIα proteins, β = GFP-CaMKIIβ proteins, β_{strong} = β – βT287D, βE'). F = σ^2_B/σ^2_T, where σ^2_B is the normalized variance between groups and σ^2_T is the normalized pooled variance. Df_B and Df_T are the degrees of freedom for σ^2_B and σ^2_T respectively. F = 1 in absence of any difference (null hypothesis). The p value is the probability that the null hypothesis is correct.*

(2) The mobility difference is not due to physical size - The magnitude of hindered diffusion has been measured for F-actin gels (7). Diffusion of 70 nm particles, twice the Stokes diameter, d of CaMKII holoenzymes, was not affected at 2 mg/ml F-actin. Mean mesh size, ε, scales with $c^{(-3/4)}$, where c is F-actin concentration, with the proportionality constant (=0.9) obtained from the diffusion of latex spheres (7). The relation between these parameters in cross-linked gels, derived by de Gennes as cited in (7), is

$$D_{lat} = D'_{Stokes}\left(exp\left(-\beta\left({}^{d}/_{\varepsilon}\right)^{2.5}\right)\right), \qquad \textbf{C}$$

where D'_{Stokes} is the Stokes diffusion coefficient in water times a correction factor for non-spherical shape. The dimensionless coefficient β is close to one. For stress fibres, the F-actin spacing determined by the α-actinin crosslinks (8) is 30 nm (9). Physical entrapment of the 25 nm CaMKII holoenzymes within fibres would decrease D'_{Stokes} 2.7 fold to 3.7 μm^2/ s. The predicted D_{lat} would have the decorating particles diffuse 10 μm^2 in 4 seconds in contrast to observation (**Movie S1**). Given an actin mesh size equivalent to an F-actin concentration of 1 mM (42 mg/ml), twice the value measured within the actin-rich lamellipodium (10); the diffusion coefficient of the particles would still be 80% of the value measured in pure water. The D_{lat} after retardation by the cortical actin gel is predicted to be at least 15 fold and 7.5 fold greater than the D_{lat} values for the strong and weak binding groups (**Figure 6A**) respectively. The low group D_{lat}s can only be due to binding interactions.

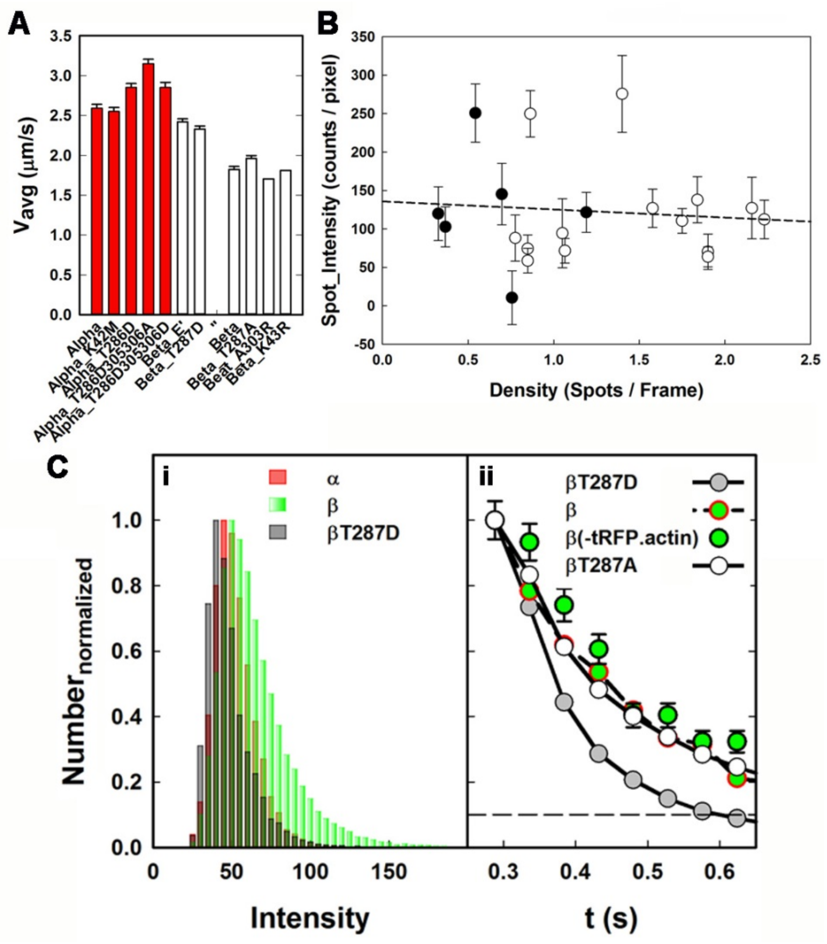

Fig. S5: Mobility differences are not due to aggregation / disassembly. A. *V_{avg} (mean $\pm$ se) values for the protein populations Red and open bars indicate different isoforms as in Figure 6.* ***B.*** *Spot intensity of αT287DT305306A variation with spot density (per 10 μm^2 evanescent field). Spot intensities were measured with the single fluorophore detection algorithm (SFDA) (2) that separates spots with PSF overlap, provided peak separation exceeds 1σ. Same culture imaged 24 hrs (closed symbols) and 38 hrs (open symbols) after transfection. Since αT287DT305306A does not bind F-actin, density in the evanescent field is an indicator of intracellular concentration. The concentrations estimated from the spot densities are in the nano-molar range but probably 10-fold higher since spots must be immobilized over 2 frames for detection. Linear regression (dashed line).* ***C. i.*** *Track population intensity histograms, with each normalized by the corresponding mode values.* ***ii.*** *βT287A and βT287D population track lifetime distributions. Control β populations with (green symbols, red edges) and without co-expressed tRFP-actin (green symbols, black edges. The distributions are normalized by the number of tracks in the first bin after the 5 frame (0.24 s) lifetime threshold. Horizontal dashed line indicates 10% of the initial population fraction.*

(3) Estimation of bound fraction and bound lifetimes – Track populations were divided into unbound and bound fractions based on a two-exponential best-fit (**Fig. S6**). The dwell-time in the evanescent field for the unbound population was set to the value obtained from the fit to the βT287D distribution. The dwell-time for the bound fraction was taken as the time for dissociation from F-actin. One bound species accounts for > 95% of the α and β populations. Bound fractions computed based on these majority species fall into two groups. Deviation from the two-exponential fits in the residual 0.05 population fraction reveals heterogeneity. A more refined statistical analysis (**Table S2**) based on a decade population decrease was not successful in establishing differences between βT287D and βE' or between βT287D and the three αT286D species.

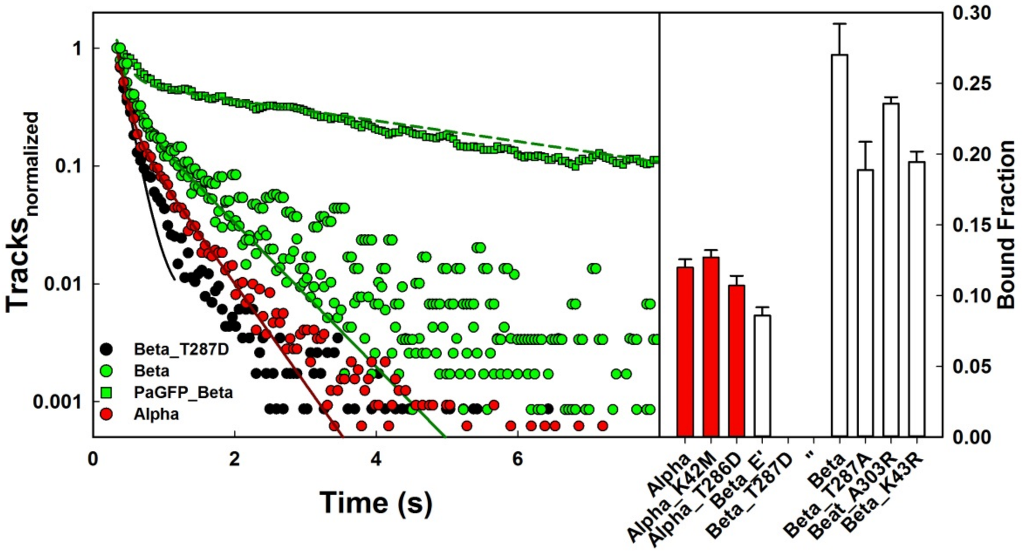

Fig. S6: Characterization of major binding modes. A. *The βT287D distribution was fit with a single exponential (black line). Two exponential best fits for the (β), (α) and PaGFP-β track distributions (coloured lines).* ***B.*** *Bound fractions ($\pm$ standard error) for the major bound CaMKII protein species estimated from two exponential fits with one exponent term fixed to the value determined from the βT287D fit (Equation 3) (k = 2.9 s^{-1} (α); 1.3 s^{-1} (β)). Cytoskeletal actin content was assumed to be the same.*

I. ANOVA

Data-Set	*Df|Residuals*	*F-Value*	*p-Value*
Plot$_{log}$(α)	3|27	2.08	0.125
Plot$_{log}$(β)	5|29	18.46	3.14xe^{-8}
Plot$_{log}$(β_{Strong})	5|17	8.24	0.001
Plot$_{log}$(α+β_{weak})	5|38	3.85	0.004

II. t-test (*wβT287D*)

CaMKII	*p-Value*	*CaMKII*	*p-Value.*
β	1.70E-07	α	0.0206
βA303R	1.20E-05	αK42M	0.0046
βK43R	1.80E-07	αT286D	0.2405
βT287A	0.00075	αT286D$_{305306D}$	0.5471
βE'	1	αT286D$_{305306A}$	1

Table S2: Track lifetime statistics. *The video-records used for the analysis of mobility were re-analysed for track lifetime differences. Df_B, Df_T, F-value, p-value and groups (α, β and β_{strong}) are as in Table S1. ($\alpha + \beta_{weak}$) = α + (βT287D, βE').* ***I.*** *ANOVA demonstrates that the GFP-CaMKII species partition into two groups based on bound life-times as well as mobility.* ***II.*** *Simultaneous pairwise t-tests conducted against βT287D, the reference for unbound species, dissect the basis for the group*

differences. The p-values indicate that the (α) and (β) isoforms as well as mutant species (βA303R), (βK43R), (βT287A) and (αK42M) bind; while absence of the spliced linker segment (βE') reduces binding to undetectable levels. T-D substitution at the primary phosphorylation site reduces binding in the β-isoform independent of the modification state of the secondary phosphorylation sites.

(4) Decay kinetics from PaGFP-fusion and GFP-fusion experiments - The difference in the track lifetime distributions measured from PALM experiments (PaGFP-fusions) and the GFP-fusion experiments (**Fig. S6**) is due to the different number of free (X) and bound (Y) fluorescent molecules imaged per unit area of the evanescent field in the two types of experiments. The number of fluorescent molecules N = (X+Y). At equilibrium; $\left[\frac{Y}{X}\right] = e^{-\Delta G/kT}$, where ΔG is is the free energy change upon binding. ka^+ and ka^- are the rates, for the X -> Y, Y -> X transitions respectively.

In PALM experiments, the number of photo-activated, fluorescent molecules *N* will have the equilibrium $\left[\frac{Y}{X}\right]$ ratio. The fluorescence decay will be proportional to the loss dN/dt over time, *t*. It will have a fast component due to diffusion (D = diffusion coefficient) of unbound molecules from the evanescent field (depth *l*) and a slow component due to dissociation of bound molecules.

$$dN/dt = Xe^{-4Dt/l^2} + Ye^{-ka^- t},$$

In the absence of tracking errors, *N* tracks whose duration is determined by their lifetimes in the evanescent field will be generated from the *N* molecules. The comparison between the fluorescence intensity decay and the track lifetime distributions (Results A6) shows that tracking errors, like photo-bleaching, have a small (~10%) effect.

For GFP-fusion experiments, track lifetime distributions will, in addition, to GFP-CaMKII molecules present at the start of the video record contain contributions from molecules (N_F) entering the evanescent field during the video record. N_F is negligible in PALM experiments since the incoming molecules will predominantly be non-fluorescent PaGFP-CaMKII. The N_F molecules effectively available to bind F-actin will be:

$$\frac{ka^+}{\left(\left(4D/l^2\right) + ka^+\right)}$$

Therefore:

$$\frac{Y_N}{X_N} < \frac{Y}{X} \text{ given } \left(4D/l^2\right)$$

is comparable or greater than ka^+. Hence, the fast component of the decay rate will be over-estimated, and the bound fraction under-estimated.

$$dN/dt = (X + X_N)e^{-4Dt/l^2} + (Y + Y_N)e^{-ka^- t},$$

The difference would increase if additional binding states are generated from the initial binding state, consistent with the log-normal lifetime distributions, as the apparent ka^+ will decrease. Tracking errors due to the greater density of fluorescent spots will also increase for the GFP-fusion experiments.

C. The Log-Normal Relation

Simulated log-normal plots (**Figure S5**) illustrate that the discrimination between the (β) and (α) isoforms depends on the time window accessed by the assay. The plots have the form “Log(F) = C_1(Log(1+C_2t))”, where F = fraction of the initial bound population, t = time, C_1 is slope. The scaling factor C_2 = 10. The log-normal relation is a consequence of the fact that both binding partners are multi-subunit assemblies that permit multiple binding reactions. Conformational flexibility of both CaMKII and F-actin will facilitate multi-subunit engagement.

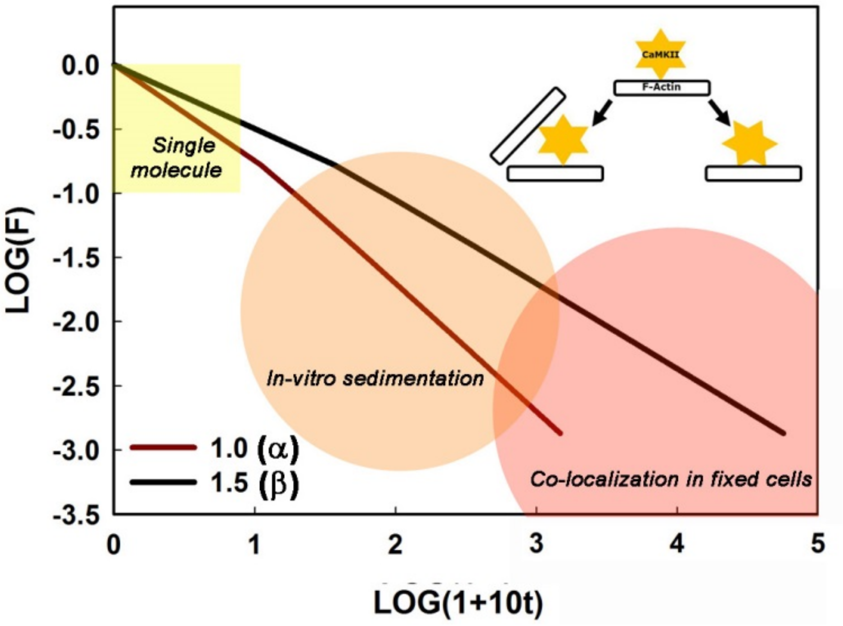

Figure S7: Simulated log-normal plots. *C_1 was taken as 1 for α and 1.5 for β to match the experimentally observed slope difference in the track lifetime relations between the two isoforms (Figure 7A). Single molecule assays monitor dissociation kinetics over a few seconds with sub-second resolution. The time for the first ten-fold population decrease (F (Log (F)) = 1(0) to 0.1(-1)) complete within 1 ((Log (1+10t)) ~ 1) second, differs by a factor of 4.7 fold for the two isoforms. In vitro bundling assays, limited by sedimentation times, capture interactions that are stable for one to many minutes. They monitor the decrease of the bound population over the second F decade (0.1 to 0.01). The β isoform takes 15.7 times as long as the α isoform over this range (1-100 seconds) for a ten-fold population decrease. Fixation times, typically minutes, limit co-localization assays in fixed cells. These assays visualize stably-bound complexes. For t>100 ((Log(1+10t)) ~ 3) seconds, < 0.1% of the bound α isoform population would remain, but probably would not be detected, as opposed to > 5% of the β population.* ***Inset:*** *Complex multi-subunit interactions between CaMKII (stars) and F-actin (rods) (schematized) can form utilizing combinations of reactions involving 2 F-actin or 2 CaMKII subunits.*

D. Supporting Movies

Movie SR1: Video-record for Figure 3A. The first section of the video shows tRFP-actin (red) and gfp-CaMKII-beta (green), played back in real time; the middle section shows the averaged intensity for both channels overlaid as a static image; the final section shows a section of the separate averaged images after local background subtraction). Co-localisation of β and F-actin is seen at actin stress fibres (long white arrows) as well as regions (membrane ruffles (short yellow arrows) expected to be rich in F-actin. CaMKII / stress fibre co-localization was quantified.

Movie S2: Video-record for Figure 5Bi. The first section of the video shows tRFP-actin (red) and βT287A (green), played back in real time; the middle section shows the averaged intensity data for both channels overlaid as a static image; then the single particle tracks are overlaid and finally the averaged tRFP-actin image is shown.

Movie S3: Video-record for Figure 5Bii. The first section of the video shows tRFP-actin (red) and βT287D (green), played back in real time; the middle section shows the averaged intensity data for both channels overlaid as a static image; then the single particle tracks are overlaid and finally the averaged tRFP-actin image is shown.

SUPPORTING REFERENCES

1. **Patterson, G. H. 2007. Photobleaching and photoactivation of fluorescent proteins for studies in cell biology. Micros Microanal 13.**
2. **Mashanov, G. I., and J. E. Molloy. 2007. Automatic detection of single fluorophores in live cells. Biophys. J. 92:2199-2211.**
3. **Schneider, C. A., W. S. Rasband, and K. W. Eliceiri. 2012. NIH Image to ImageJ: 25 years of image analysis. Nature methods 9:671-675.**
4. **Manders, E. M., Verbeek, F.J and Aten, J.A. 1993. Measurement of co-localization of objects in dual-colour confocal images. J. Microscopy 169:375-382.**
5. **Costes, S. V., D. Daelemans, E. H. Cho, Z. Dobbin, G. Pavlakis, and S. Lockett. 2004. Automatic and quantitative measurement of protein-protein colocalization in live cells. Biophys. J. 86:3993-4003.**
6. **Qian, H., M. P. Sheetz, and E. L. Elson. 1991. Single particle tracking. Analysis of diffusion and flow in two-dimensional systems. Biophys. J. 60:910-921.**
7. **Schmidt, C. F., Barmann, M., Isenberg, G., and Sackmann, E. 1989. Chain dynamics, mesh size and diffusive transport in networks of polymerized actin. A quasielastic light scattering and microfluorescence study. Macromolecules 22:3638-3649.**
8. **Pellegrin, S., and H. Mellor. 2007. Actin stress fibres. J. Cell Sci. 120:3491-3499.**
9. **Meyer, R. K., and U. Aebi. 1990. Bundling of actin filaments by alpha-actinin depends on its molecular length. J. Cell Biol. 110:2013-2024.**
10. **Koestler, S. A., K. Rottner, F. Lai, J. Block, M. Vinzenz, and J. V. Small. 2009. F- and G-actin concentrations in lamellipodia of moving cells. PloS one 4:e4810.**
